# In depth search of the Sequence Read Archive database reveals global distribution of the emerging pathogenic fungus *Scedosporium aurantiacum*

**DOI:** 10.1093/mmy/myac019

**Published:** 2022-03-04

**Authors:** Laszlo Irinyi, Michael Roper, Wieland Meyer

**Affiliations:** Molecular Mycology Research Laboratory, Centre for Infectious Diseases and Microbiology, Faculty of Medicine and Health, Sydney Medical School, Westmead Clinical School, The University of Sydney, Westmead, NSW 2145, Australia; Sydney Institute for Infectious Diseases, The University of Sydney, Westmead, NSW 2145, Australia; Westmead Institute for Medical Research, Westmead, NSW 2145, Australia; Division of Biomedical Science and Biochemistry, Australian National University, Canberra, ACT 2601, Australia; Molecular Mycology Research Laboratory, Centre for Infectious Diseases and Microbiology, Faculty of Medicine and Health, Sydney Medical School, Westmead Clinical School, The University of Sydney, Westmead, NSW 2145, Australia; Sydney Institute for Infectious Diseases, The University of Sydney, Westmead, NSW 2145, Australia; Westmead Institute for Medical Research, Westmead, NSW 2145, Australia; Westmead Hospital (Research and Education Network), Westmead, NSW 2145, Australia

**Keywords:** *Scedosporium aurantiacum*, DNA metabarcoding, SRA database, environment, ITS sequence

## Abstract

*Scedosporium* species are emerging opportunistic fungal pathogens causing various infections mainly in immunocompromised patients, but also in immunocompetent individuals, following traumatic injuries. Clinical manifestations range from local infections, such as subcutaneous mycetoma or bone and joint infections, to pulmonary colonization and severe disseminated diseases. They are commonly found in soil and other environmental sources. To date *S. aurantiacum* has been reported only from a handful of countries. To identify the worldwide distribution of this species we screened publicly available sequencing data from fungal metabarcoding studies in the Sequence Read Archive (SRA) of The National Centre for Biotechnology Information (NCBI) by multiple BLAST searches. *S. aurantiacum* was found in 26 countries and two islands, throughout every climatic region. This distribution is like that of other *Scedosporium* species. Several new environmental sources of *S. aurantiacum* including human and bovine milk, chicken and canine gut, freshwater, and feces of the giant white-tailed rat (*Uromys caudimaculatus*) were identified. This study demonstrated that raw sequence data stored in the SRA database can be repurposed using a big data analysis approach to answer biological questions of interest.

**Lay summary:**

To understand the distribution and natural habitat of *S. aurantiacum*, species-specific DNA sequences were searched in the SRA database. Our large-scale data analysis illustrates that *S. aurantiacum* is more widely distributed than previously thought and new environmental sources were identified.

## Introduction

*Scedosporium* is a genus of fungi in the *Microascaceae* family of the *Ascomycota* and compromises saprotrophic mold species, mainly living on decaying organic matter and are found in soil, sewage, and contaminated water.^1^^,^^2^ The genus currently includes ten species (*S. aurantiacum, S. minutisporum, S. desertorum, S. cereisporum, S. dehoogii, S. angustum, S. apiospermum, S. boydii, S. ellipsoideum*, and *S. fusarium*). Five of them have been found to be clinically relevant: *Scedosporium apiospermum, S. boydii, S. aurantiacum, S. dehoogii and S. minutisporum*.^3^*Scedosporium* species can cause localized and severe disseminated infections depending on the immune status of the host.^4^ They are responsible for 25% of non-*Aspergillus* mold infections in organ transplant recipients in the USA and are associated with the occurrence of major trauma.^5–7^ They have also been reported from patients with pulmonary conditions, such as cystic fibrosis, but their significance in these conditions is uncertain.^8–10^ Among them, *S. boydii* and *S. apiospermum* are the most frequently isolated species, but in some regions *S. aurantiacum* is more common.^11^

*S. aurantiacum* is an opportunistic pathogen capable of causing a wide variety of localized and superficial infections, such as malignant otitis externa, osteomyelitis, invasive sinusitis, keratitis, and pneumonia.^7^^,^^12^*S. aurantiacum*, separated from other *Scedosporium* species by molecular markers, such as *β-tubulin*, *calmodulin*, and the internal transcribed spacer (ITS) region, was first proposed as a new species in 2005.^13^ Several studies have been undertaken to describe the ecology and environmental distribution of different *Scedosporium* species mainly in Europe, such as in France,^14^ Austria, and The Netherlands,^1^ as well as in Australia,^11^ Thailand,^15^^,^^16^ Mexico^17^ and Morocco.^18^

The distribution of the *Scedosporium* spp. indicated geographical differences,^1^^,^^14^ with *S. aurantiacum* to be mainly abundant in Australia^11^ and in agricultural areas in the west of France,^14^ with additional reports from The Netherlands, Morocco, Thailand and Mexico.^1,15–18^ In Australia, more than 50% of all environmental *Scedosporium* isolates were *S. aurantiacum*, which coincides with the relative high prevalence of scedosporiosis and their presence as colonizers in CF patients in Australia.^12^^,^^19^ In total, clinical isolates of *S. aurantiacum* have been reported from ten countries, including Australia, Austria, France, Germany, Italy, Japan, Netherlands, South Korea, Spain, and United States of America. In comparison, environmental isolates of *S. aurantiacum* have been reported from 14 countries: Australia, Austria, France, Germany, Italy, Latvia, Mexico, Morocco, Nepal, Netherlands, Russia, Spain, Thailand, and UK, where it was mainly reported from soil, compost and sewage water.^1,11,14–18,20–23^

To expand the knowledge of the environmental distribution of microorganisms, metabarcoding has become the main tool used to characterize complex microbial and other communities from microbial ecology studies to infectious disease surveillance.^24–26^ DNA metabarcoding is the simultaneous identification of a large set of taxa present in a single complex sample.^27^ The approach combines the concept of DNA barcoding^28^ with the application of next generation sequencing (NGS). It uses short DNA sequences (barcodes) to standardize the identification of organisms from all kingdoms down to species level by comparison to a reference sequence collection of well identified species.^28^^,^^29^ Developments in NGS sequencing has made it possible to generate and analyze millions of targeted amplicons (barcodes) amplified by polymerase chain reaction (PCR) from thousands of mixed DNA templates within the same sample simultaneously to determine the species composition of the sample.^29^ Metabarcoding is currently the standard tool and the most efficient method for culture-independent assessment of microbiomes.^30^

In fungi, the internal transcribed spacer (ITS) region was established as the primary fungal DNA barcode in 2012.^31^ This is due to its multicopy nature and its easy amplification with universal primers that are compatible with most fungal species.^31^^,^^32^ It has been extensively used in both molecular systematics and ecological studies in fungi over three decades.^2^^,^^33^^,^^34^ The ITS region consists of the ITS1 and ITS2 regions separated by the 5.8S gene and is located between the 18S (SSU) and 28S (LSU) genes in the nrDNA repeat unit.^33^ With traditional Sanger sequencing the entire ITS region, which ranges between 280 and 800 bp, has been targeted for molecular identification purposes.^35^ However, in metabarcoding studies, either the ITS1 or ITS2 region has been amplified and sequenced by NGS technologies, due to the fact that the entire ITS region is too long for commonly used sequencing platforms, such as Illumina, Ion Torrent or the phased out 454 sequencing from Roche.^2^^,^^36^

As molecular identification of various microbial samples has become an essential part of different studies worldwide it has provided new insights into the diversity and ecology of many different fungal communities (mycobiome).^37^^,^^38^ As a result, large amounts of partial ITS sequences have been generated by NGS and deposited in public sequence databases, such as the Sequence Read Archive (SRA) of the National Institutes of Health (NIH), which is the primary international public archive of high-throughput sequencing data established under the guidance of the International Nucleotide Sequence Database Collaboration (INSDC).^39^ SRA stores raw sequence data from different NGS technologies, including Roche 454, Illumina, Ion Torrent, Pacific Biosciences and Oxford Nanopore Technologies. SRA has the largest, most diverse collection of NGS data from human, non-human and microbial sources.

The current study screened the publicly available metabarcoding data of NIH's SRA database containing fungal sequence data to identify the geographical distribution, and potential ecological sources and reservoirs of the emerging human pathogenic fungus *S. aurantiacum*, serving as pilot study to highlight the potential of repurposing of publicly available raw sequence datasets to answer major biological and public heath questions.

## Methods

All data used in this study are publicly available in the SRA database (https://www.ncbi.nlm.nih.gov/sra). In this study, a subset of SRA datasets containing the ITS1 or ITS2 sequences from fungal metabarcoding studies were identified (192 117) as of June 2020 by using the following keywords: ‘fungi’, ‘fungal diversity’ and ‘ITS region’ on the web interface of SRA database. The query outputs were combined, and duplicate datasets were removed based on their unique identification number.

The SRA toolkit version 2.10.7^40^ and the basic local alignment search tool (BLAST) implemented in the toolkit^41^ were used to screen and identify the datasets containing *S. aurantiacum* ITS sequences. The query sequence contained the full ITS region (ITS1 + 5.8S + ITS2) and partial SSU and LSU sequences (totalling 661 bp), which was extracted from the contig of the whole-genome assembly of the *S. aurantiacum* strain WM 09.24 (GenBank Accession number: JUDQ01000713.1). The herein used similarity identity threshold for the BLAST analysis was 99% and the E-value was set to less than 1E-80 to minimize the false positive hits. The identified sequence data from positive matches containing either the ITS1 or ITS2 region of *S. aurantiacum* were then manually checked.

All the metadata associated and available for the *S. aurantiacum* positive SRA datasets (Supplementary Table 1), including information about their geographical locations and isolation sources, were downloaded from the SRA database. In some cases, the metadata was incomplete in the SRA database, which prompted screening the relevant publications associated with the SRA data to extract the metadata.

The following databases PubMed, Scopus, Web of Science, and Google Scholar as of 31 of July 2020 were screened to obtain published data about the occurrence and ecological distribution of *S. aurantiacum* in clinical and environment samples using the keyword *S. aurantiacum*. In addition, the Nucleotide database of NCBI, Westmead Mycology Culture Collection and the Culture collection of fungi and yeasts of Westerdijk Fungal Biodiversity Institute was screened for additional clinical and environmental isolates of *S. aurantiacum.*

Individual geographical locations obtained from the *S. aurantiacum* positive SRA datasets, together with the published unique locations of clinical and environmental occurrence of *S. aurantiacum* were plotted on the world map using the QGIS, geographic information system (version 3.10.9-A Coruña with Grass 7.8.3).^42^

## Results

**Figure 1.**
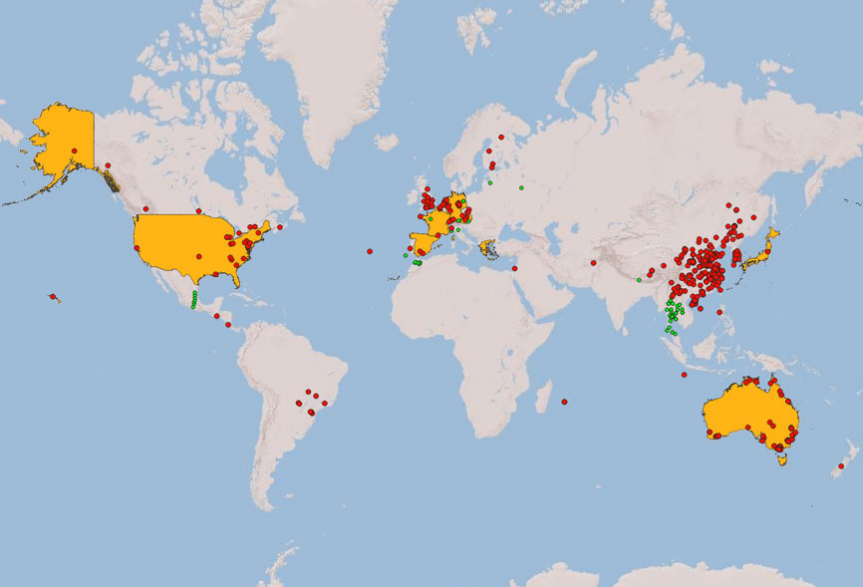
Geographical distribution of *Scedosporium aurantiacum*. Countries in yellow indicate the location of previously published clinical isolates. Green dots represent the location of environmental isolates previously reported. Red dots represent the location of SRA datasets identified in the current study containing either the ITS1 or ITS2 sequences of *S. aurantiacum*.

The described database search identified 1706 SRA sequence data sets that contained either the ITS1 or ITS2 region of *S. aurantiacum* (Supplementary Table 1). After assessing the associated metadata together with the published unique locations of clinical and environmental occurrence of *S. aurantiacum* (Table 1) they were plotted on the world map using the QGIS software (Figure 1). The obtained results from screening the SRA database indicate that *S. aurantiacum* has a wide geographic distribution (Figure 1). All in all, *S. aurantiacum* was identified in 26 countries and two islands (Reunion and Christmas Island) (Table 1). Among them, *S. aurantiacum* has not been reported before in: Afghanistan, Belgium, Brazil, Canada, China, Christmas Island, Costa Rica, Czech Republic, El Salvador, Finland, Israel, New Zealand, Philippines, Portugal, Reunion, Singapore, Switzerland, and United Kingdom. The highest number of *S. aurantiacum* positive SRA data were from China (965), followed by the United Kingdom (241) and Australia (135).

**Table 1.** Geographical distribution of *Scedosporium aurantiacum* based on metabarcoding datasets in the Sequence Read Archive database. Countries in bold indicates locations where *S. aurantiacum* has not been previously reported.

Location of SRA data with ITS1/ITS2 sequences of *S. aurantiacum*	Number of SRA datasets with ITS1/ITS2 sequences of *S. aurantiacum*
**Afghanistan**	1
Australia	135
Austria	9
**Belgium**	34
**Brazil**	21
**Canada**	79
**China**	965
**Christmas Island**	1
**Costa Rica**	1
**Czech Republic**	4
**El Salvador**	2
**Finland**	8
France	3
Germany	26
**Israel**	1
**Italy**	1
Japan	1
Netherlands	15
**New Zealand**	1
**Philippines**	1
**Portugal**	2
**Reunion**	2
**Singapore**	1
South Korea	22
Spain	6
**Switzerland**	14
**United Kingdom**	241
United States of America	109

The environmental sources of the *S. aurantiacum* positive SRA data included mainly various soils, sludge, and sediment samples (88% of the samples) (Table 2). The herein reported study also identified several new sources from which *S. aurantiacum* had not yet been reported, such as human and bovine milk, chicken and canine gut, freshwater, and feces of the giant white-tailed rat (*Uromys caudimaculatus*) (Table 2).

**Table 2.** Environmental sources of *Scedosporium aurantiacum* based on metabarcoding datasets in the Sequence Read Archive database. Source of sequence in bold indicates locations where *S. aurantiacum* has not been previously reported.

Origin of SRA data with ITS1/ITS2 sequences of *S. aurantiacum*	Number of SRA datasets with ITS1/ITS2 sequences of *S. aurantiacum*
**Air samples**	3
**Anaerobic reactor**	1
**Bovine milk**	2
**Canine gut**	2
**Chicken gut**	5
Compost	11
**Dust**	2
**Early phase of fermentation**	1
**Feces of giant white-tailed rat**	1
Freshwater	3
Human lung	3
**Human milk**	3
**Mangrove**	4
**Rhizosphere**	125
**Rumen**	30
Sediment	17
Sewage sludge	61
Soil	1405
**Spent growing medium**	18
**Straw residue**	1
**Tree hollow**	7
**Wood**	1

## Discussion

So far, *S. aurantiacum* has been reported from only a few countries, with limited studies being done to assess its global distribution. Environmental isolates of *S. aurantiacum* have only been reported previously from Australia,^11^ France,^3^^,^^14^ The Netherlands,^1^ Morocco,^18^ Thailand^15^^,^^16^ and Mexico.^17^ Clinical reports of *S. aurantiacum* have previously not demonstrated any association with environmental isolates of the same species. Till now both clinical and environmental isolates have been reported only from Australia,^11^^,^^12^ Austria,^1^ France,^3^^,^^14^ and The Netherlands.^1^^,^^43^ Clinical cases of *S. aurantiacum* have been reported from Japan,^44^ South Korea,^45^ and Spain,^46^ while environmental isolates have been reported from Italy,^20^ Mexico,^17^ Morocco^18^ and Thailand.^15^^,^^16^ The present study searched the publicly available raw sequence data of the NCBI SRA database to assess the geographical distribution and environmental niches and reservoirs of the emerging fungal pathogen *S. aurantiacum*. It identified the occurrence of *S. aurantiacum* in 16 additional countries and two islands from where it had not been reported previously (Table 1). The highest number of locations was found in datasets from China, the United Kingdom and Australia (Table 1). However, it is important to note that this high numbers are very likely due to extensive number of metabarcoding studies carried out in these countries. As metabarcoding studies are still relatively expensive (∼$100 US per sample) they are still infeasible in many countries.

The obtained results suggest that *S. aurantiacum* has a wide distribution rather than being limited to certain countries. One of the reasons *S. aurantiacum* has not been reported more often could be possible misidentification since this species cannot be morphologically distinguished from the closely related species *S*. *apiospermum*, as it was only recently described on the basis of sequence analysis of a number of genetic loci.^13^ As such, it can be assumed that many routine clinical laboratories, in which molecular identification methods are not available or too expensive, will misidentify this species. Another reason could be that many countries have not reported *S. aurantiacum* infections in scientific papers despite correctly identifying them. For example, a recent study about the identification and susceptibility of clinically relevant *Scedosporium* spp. in China has not reported any *S. aurantiacum* isolates,^47^ which is in sharp contrast with the herein obtained metabarcoding based findings.

The screening of the SRA database also showed that the distribution of *S. aurantiacum* does not show any clear relationship with climate conditions, as the obtained results show that *S. aurantiacum* specific sequences have been found in metabarcoding datasets obtained in samples from temperate, arid, and tropical zones, as well as in the Mediterranean and tundra regions.

The environmental sources of *S. aurantiacum* as identified in the current study remain predominantly various soils, sewage and sediments as has been reported previously.^1,3,16–18^ The current study also identified additional sources, such as human and bovine milk, chicken and canine gut, freshwater, and feces of the giant white-tailed rat (*Uromys caudimaculatus*).

Having shown that *S. aurantiacum* has a wide distribution it is important to see the current study in the light of its biases and limits. To discuss these biases in detail is out of the scope of this paper. However, a non-exhaustive list includes statistical sampling error, sequencing error, and the BLAST algorithm itself.^48–50^ From the technological side of metabarcoding, there are many well documented technical artifacts, including DNA extraction and amplification as well as PCR biases, which can result in the non-detection of certain species even if they are present in the samples.^bib51–56^ Another potential source of bias and error are the bioinformatic tools used, e.g., the BLAST algorithm and the SRA database search function. Despite being the most widely used alignment based sequence similarity search algorithm^57^ it comes with major disadvantages, being generally memory and time consuming, limiting its use for large-scale sequence data. The selection of relevant subset data from the complete SRA database (∼18 petabytes) is not without any challenge. Although, many scientific journals require submitting raw sequence data to the SRA database prior publication, there are few standards about how much associated metadata should be submitted together with the raw sequence data. In a number of cases, this practice resulted in insufficient or incomplete metadata sets associated with the raw sequence data, which makes the subsequent filtering process challenging and incomplete. Sometimes, there is not even any information submitted whether the dataset contains fungal ITS sequence or not. In other cases, the metadata is only available in the publication but not in the SRA database.

Overall, the current study identified 192 117 publicly available datasets containing either ITS1 or ITS2 sequences. With a rough estimation of about $100 US sequencing cost per sample, the herein presented study screened ∼$19.21 million US worth of sequence data from many countries to assess the global ecological distribution of an emerging opportunistic fungal pathogens. This study about the emerging human pathogen, *S. aurantiacum* massively expanded our knowledge of its natural reservoir as the potential for being the source of human infection. The herein described wider environmental presence if this human pathogen alerts public health authorities to pay attention to these potential infection sources, when accessing the risk for vulnerable individuals. It highlights the potential application of the SRA database to search for the geographical and environmental distribution of fungal species or in fact any microorganism to answer questions about disease reservoirs, potentially enabling the prediction of outbreaks and to increase the preparedness of public health authorities. It should be viewed as a pilot study using the vast hidden treasure of the SRA database to answer certain biological questions.

## Supplementary Material

myac019_Supplemental_File

## References

[1] Kaltseis J , Rainer J, De Hoog GS. Ecology of *P**seudallescheria* and *S**cedosporium* species in human-dominated and natural environments and their distribution in clinical samples. Med Mycol. 2009; 47: 398–405. 19085459 10.1080/13693780802585317

[2] Buee M , Reich M, Murat C et al. 454 Pyrosequencing analyzes of forest soils reveal an unexpectedly high fungal diversity. New Phytol. 2009; 184: 449–456. 19703112 10.1111/j.1469-8137.2009.03003.x

[3] Rougeron A , Giraud S, Alastruey-Izquierdo A et al. Ecology of *S**cedosporium* species: present knowledge and future research. Mycopathologia. 2018; 183: 185–200. 28929280 10.1007/s11046-017-0200-2

[4] Ramirez-Garcia A , Pellon A, Rementeria A et al. *Scedosporium* and *L**omentospora*: an updated overview of underrated opportunists. Med Mycol. 2018; 56: S102–S125. 10.1093/mmy/myx113 29538735

[5] Husain S , Muñoz P, Forrest G et al. Infections due to *S**cedosporium**apiospermum* and *S**cedosporium**prolificans* in transplant recipients: clinical characteristics and impact of antifungal agent therapy on outcome. Clin Infect Dis. 2005; 40: 89–99. 15614697 10.1086/426445

[6] Husain S , Alexander BD, Munoz P et al. Opportunistic mycelial fungal infections in organ transplant recipients: emerging importance of non-*Aspergillus* mycelial fungi. Clin Infect Dis. 2003; 37: 221–229. 12856215 10.1086/375822

[7] Cortez KJ , Roilides E, Quiroz-Telles F et al. Infections caused by *S**cedosporium* spp. Clin Microbiol Rev. 2008; 21: 157–197. 18202441 10.1128/CMR.00039-07 PMC2223844

[8] Cimon B , Carrère J, Vinatier JF et al. Clinical significance of *S**cedosporium**apiospermum* in patients with cystic fibrosis. Eur J Clin Microbiol Infect Dis. 2000; 19: 53–56. 10706182 10.1007/s100960050011

[9] Guarro J , Kantarcioglu AS, Horré R et al. *Scedosporium apiospermum*: changing clinical spectrum of a therapy-refractory opportunist. Med Mycol. 2006; 44: 295–327. 16772225 10.1080/13693780600752507

[10] Paugam A , Baixench MT, Demazes-Dufeu N et al. Characteristics and consequences of airway colonization by filamentous fungi in 201 adult patients with cystic fibrosis in France. Med Mycol. 2010; 48: S32–S36. 21067327 10.3109/13693786.2010.503665

[11] Harun A , Gilgado F, Chen SC. Abundance of *P**seudallescheria**/**S**cedosporium* species in the Australian urban environment suggests a possible source for scedosporiosis including the colonization of airways in cystic fibrosis. Med Mycol. 2010; 48: S70–S76. 21067333 10.3109/13693786.2010.515254

[12] Heath CH , Slavin MA, Sorrell TC et al. Population-based surveillance for scedosporiosis in Australia: epidemiology, disease manifestations and emergence of *S**cedosporium**aurantiacum* infection. Clin Microbiol Infect. 2009; 15: 689–693. 19549223 10.1111/j.1469-0691.2009.02802.x

[13] Gilgado F , Cano J, Gené J et al. Molecular phylogeny of the *P**seudallescheria**boydii* species complex: proposal of two new species. J Clin Microbiol. 2005; 43: 4930–4942. 16207945 10.1128/JCM.43.10.4930-4942.2005 PMC1248451

[14] Rougeron A , Schuliar G, Leto J et al. Human-impacted areas of France are environmental reservoirs of the *P**seudallescheria**boydii/Scedosporium apiospermum* species complex. Environ Microbiol. 2015; 17: 1039–1048. 24684308 10.1111/1462-2920.12472

[15] Luplertlop N , Pumeesat P, Muangkaew W et al. Environmental screening for the *S**cedosporium**apiospermum* species complex in public parks in Bangkok, Thailand. PLoS ONE. 2016; 11: e0159869. 27467209 10.1371/journal.pone.0159869 PMC4965192

[16] Luplertlop N , Muangkaew W, Pumeesat P et al. Distribution of *S**cedosporium* species in soil from areas with high human population density and tourist popularity in six geographic regions in Thailand. PLoS ONE. 2019; 14: e0210942. 30673761 10.1371/journal.pone.0210942 PMC6343921

[17] Elizondo-Zertuche M , de JT-RR, Robledo-Leal E et al. Molecular identification and in vitro antifungal susceptibility of *S**cedosporium* complex isolates from high-human-activity sites in Mexico. Mycologia 2017; 109: 874–881. 29494271 10.1080/00275514.2017.1416260

[18] Mouhajir A , Poirier W, Angebault C et al. *Scedosporium* species in soils from various biomes in Northwestern Morocco. PLoS ONE. 2020; 15: e0228897. 32092070 10.1371/journal.pone.0228897 PMC7039527

[19] Laurence D , Azian H, Sharon CAC et al. Molecular typing of Australian *S**cedosporium* isolates showing genetic variability and numerous *S**. aurantiacum*. Emerg Infect Dis. 2008; 14: 282. 18258122 10.3201/eid1402.070920 PMC2600218

[20] Di Piazza S , Houbraken J, Meijer M et al. Thermotolerant and thermophilic mycobiota in different steps of compost maturation. Microorganisms. 2020; 8: 880. 10.3390/microorganisms8060880 PMC7355412 32545162

[21] Grantina-Ievina L , Andersone U, Berkolde-Pīre D et al. Critical tests for determination of microbiological quality and biological activity in commercial vermicompost samples of different origins. Appl Microbiol Biotechnol. 2013; 97: 10541–10554. 23504062 10.1007/s00253-013-4825-x

[22] Marfenina OE , Danilogorskaya AA. Effect of elevated temperatures on composition and diversity of microfungal communities in natural and urban boreal soils, with emphasis on potentially pathogenic species. Pedobiologia. 2017; 60: 11–19.

[23] Robledo-Mahón T , Calvo C, Aranda E. Enzymatic potential of bacteria and fungi isolates from the sewage sludge composting process. Appl Sci. 2020; 10: 7763.

[24] Tedersoo L , Bahram M, Polme S et al. Fungal biogeography. Global diversity and geography of soil fungi. Science. 2014; 346: 1256688. 25430773 10.1126/science.1256688

[25] Tonge DP , Pashley CH, Gant TW. Amplicon –based metagenomic analysis of mixed fungal samples using proton release amplicon sequencing. PLoS ONE. 2014; 9: e93849. 24728005 10.1371/journal.pone.0093849 PMC3984086

[26] Nguyen LDN , Viscogliosi E, Delhaes L. The lung mycobiome: an emerging field of the human respiratory microbiome. Front Microbiol. 2015; 6: 89. 25762987 10.3389/fmicb.2015.00089 PMC4327734

[27] Taberlet P , Prud'Homme SM, Campione E et al. Soil sampling and isolation of extracellular DNA from large amount of starting material suitable for metabarcoding studies. Mol Ecol. 2012; 21: 1816–1820. 22300434 10.1111/j.1365-294X.2011.05317.x

[28] Hebert PD , Cywinska A, Ball SL et al. Biological identifications through DNA barcodes. Philos Trans R Soc Lond B Biol Sci. 2003; 270: 313–321. 10.1098/rspb.2002.2218 PMC1691236 12614582

[29] Taberlet P , Coissac E, Pompanon F et al. Towards next-generation biodiversity assessment using DNA metabarcoding. Mol Ecol. 2012; 21: 2045–2050. 22486824 10.1111/j.1365-294X.2012.05470.x

[30] Tang J , Iliev ID, Brown J et al. Mycobiome: approaches to analysis of intestinal fungi. J Immunol Methods. 2015; 421: 112–121. 25891793 10.1016/j.jim.2015.04.004 PMC4451377

[31] Schoch C , Seifert K, Huhndorf S et al. Nuclear ribosomal internal transcribed spacer (ITS) region as a universal DNA barcode marker for fungi. Proc Natl Acad Sci USA. 2012; 109: 6241–6246. 22454494 10.1073/pnas.1117018109 PMC3341068

[32] Vilgalys R , Gonzalez D. Organization of ribosomal DNA in the basidiomycete *T**hanatephorus**praticola*. Curr Genet. 1990; 18: 277–280. 2249259 10.1007/BF00318394

[33] White TJ , Bruns T, Lee S et al. Amplification and direct sequencing of fungal ribosomal RNA genes for phylogenetics, p 315–322. *In* Innis, MA, Gelfandm, DH, Sninsky, JJ, White, TJ (ed), PCR Protocols: a Guide to Methods and Applications, 1st ed. Academic Press, New York, 1990.

[34] Anslan S , Nilsson RH, Wurzbacher C et al. Great differences in performance and outcome of high-throughput sequencing data analysis platforms for fungal metabarcoding. Myco Keys. 2018. doi:10.3897/mycokeys.39.28109:29-40. 10.3897/mycokeys.39.28109 PMC6160831 30271256

[35] Irinyi L , Serena C, Garcia-Hermoso D et al. International Society of Human and Animal Mycology (ISHAM)-ITS reference DNA barcoding database-the quality controlled standard tool for routine identification of human and animal pathogenic fungi. Med Mycol. 2015; 53: 313–337. 25802363 10.1093/mmy/myv008

[36] Aguayo J , Fourrier-Jeandel C, Husson C et al. Assessment of passive traps combined with high-throughput sequencing to study airborne fungal communities. Appl Environ Microbiol. 2018; 84: e02637–17. 29572213 10.1128/AEM.02637-17 PMC5960964

[37] Wu B , Hussain M, Zhang W et al. Current insights into fungal species diversity and perspective on naming the environmental DNA sequences of fungi. Mycology. 2019; 10: 127–140. 31448147 10.1080/21501203.2019.1614106 PMC6691916

[38] Brien HE , Parrent JL, Jackson JA et al. Fungal community analysis by large-scale sequencing of environmental samples. Appl Environ Microbiol. 2005; 71: 5544. 16151147 10.1128/AEM.71.9.5544-5550.2005 PMC1214672

[39] Cochrane G , Karsch-Mizrachi I, Nakamura Y. The International Nucleotide Sequence Database Collaboration. Nucleic Acids Res. 2011; 39: D15–D18. 21106499 10.1093/nar/gkq1150 PMC3013722

[40] Team STD. SRA Toolkit, Nation of National Center for Biotechnology, 2020. http://ncbi.github.io/sra-tools/.

[41] Altschul SF , Gish W, Miller W et al. Basic local alignment search tool. J Mol Biol. 1990; 215: 403–410. 2231712 10.1016/S0022-2836(05)80360-2

[42] QGIS T . QGIS Geographic Information System, v3.10.9-A Coruña with Grass 7.8.3. Open Source Geospatial Foundation Project, 2020. http://qgis.org.

[43] Kooijman CM , Kampinga GA, de Hoog GS et al. Successful treatment of *S**cedosporium**aurantiacum* osteomyelitis in an immunocompetent patient. Surg Infect (Larchmt). 2007; 8: 605–610. 18171120 10.1089/sur.2006.038

[44] Nakamura Y , Suzuki N, Nakajima Y et al. *Scedosporium aurantiacum* brain abscess after near-drowning in a survivor of a tsunami in Japan. Respir Invest. 2013; 51: 207–211. 10.1016/j.resinv.2013.07.001 24238227

[45] Kim H , Ahn J-Y, Chung I-Y et al. A case report of infectious scleritis with corneal ulcer caused by *S**cedosporium**aurantiacum*. Medicine. 2019; 98: e16063–e16063. 31277100 10.1097/MD.0000000000016063 PMC6635299

[46] Alastruey-Izquierdo A , Cuenca-Estrella M, Monzón A et al. Prevalence and susceptibility testing of new species of *P**seudallescheria* and *S**cedosporium* in a collection of clinical mold isolates. Antimicrob Agents Chemother. 2007; 51: 748–751. 17101671 10.1128/AAC.01177-06 PMC1797781

[47] Wang H , Wan Z, Li R et al. Molecular identification and susceptibility of clinically relevant *S**cedosporium* spp. in China. Biomed Res Int. 2015; 2015: 109656–109656. 26550562 10.1155/2015/109656 PMC4615859

[48] Simossis V , Kleinjung J, Heringa J. An overview of multiple sequence alignment. Curr Protoc Bioinformatics Chapter. 2003; 3: Unit 3.7. 10.1002/0471250953.bi0307s03 18428699

[49] Chan CX , Ragan MA. Next-generation phylogenomics. Biol Direct. 2013; 8: 3–3. 23339707 10.1186/1745-6150-8-3 PMC3564786

[50] Zielezinski A , Girgis HZ, Bernard G et al. Benchmarking of alignment-free sequence comparison methods. Genome Biol. 2019; 20: 144. 31345254 10.1186/s13059-019-1755-7 PMC6659240

[51] Reeder J , Knight R. The ‘rare biosphere’: a reality check. Nat Methods. 2009; 6: 636–637. 19718016 10.1038/nmeth0909-636

[52] Medinger R , Nolte V, Pandey RV et al. Diversity in a hidden world: potential and limitation of next-generation sequencing for surveys of molecular diversity of eukaryotic microorganisms. Mol Ecol. 2010; 19: 32–40. 20331768 10.1111/j.1365-294X.2009.04478.x PMC2953707

[53] Tedersoo L , Nilsson RH, Abarenkov K et al. 454 Pyrosequencing and Sanger sequencing of tropical mycorrhizal fungi provide similar results but reveal substantial methodological biases. New Phytol. 2010; 188: 291–301. 20636324 10.1111/j.1469-8137.2010.03373.x

[54] Tedersoo L , Anslan S, Bahram M et al. Shotgun metagenomes and multiple primer pair-barcode combinations of amplicons reveal biases in metabarcoding analyses of fungi. MycoKeys. 2015; 10: 1–43.

[55] Frau A , Kenny JG, Lenzi L et al. DNA extraction and amplicon production strategies deeply inf luence the outcome of gut mycobiome studies. Sci Rep. 2019; 9: 9328. 31249384 10.1038/s41598-019-44974-x PMC6597572

[56] Makiola A , Dickie IA, Holdaway RJ et al. Biases in the metabarcoding of plant pathogens using rust fungi as a model system. Microbiology Open. 2019; 8: e00780. 10.1002/mbo3.780 PMC6612544 30585441

[57] Boratyn GM , Camacho C, Cooper PS et al. BLAST: a more efficient report with usability improvements. Nucleic Acids Res. 2013; 41: W29–W33. 23609542 10.1093/nar/gkt282 PMC3692093

