# Supplementary material for: In depth search of the Sequence Read Archive database reveals global distribution of the emerging pathogenic fungus Scedosporium aurantiacum
Source: Med Mycol. 2022 Mar 4;60(4):myac019. doi: 10.1093/mmy/myac019 (PMC8994208; doi:10.1093/mmy/myac019)
Supplement: myac019_Supplemental_File [file myac019_supplemental_file.docx]

**Supplementary Table 1:** Sequence data and their associated metadata in the NCBI Sequence Read Archive containing the partial internal transcribed regions (ITS), either ITS1 or ITS2, of *Scedosporium aurantiacum* identified. The Identity (%) means the percent identity for the best match provided by the BLAST analysis.

| Run | Experiment | BioProject | SRA Study | Identity (%) | Isolation Source | Country |
| --- | --- | --- | --- | --- | --- | --- |
| ERR2610423 | ERX2627079 | PRJEB27065 | ERP109105 | 99.27 | Soil | Afghanistan |
| ERR3077844 | ERX3138732 | PRJEB30857 | ERP113345 | 99.3 | Soil | Australia |
| ERR3077845 | ERX3138733 | PRJEB30857 | ERP113345 | 99.66 | Soil | Australia |
| ERR3077848 | ERX3138736 | PRJEB30857 | ERP113345 | 99.66 | Soil | Australia |
| ERR3077852 | ERX3138740 | PRJEB30857 | ERP113345 | 100 | Soil | Australia |
| ERR3077859 | ERX3138747 | PRJEB30857 | ERP113345 | 99.66 | Soil | Australia |
| SRR3453375 | SRX1729221 | PRJNA317932 | SRP074055 | 99.23 | Soil | Australia |
| SRR3453382 | SRX1729228 | PRJNA317932 | SRP074055 | 99.24 | Soil | Australia |
| SRR3453387 | SRX1729233 | PRJNA317932 | SRP074055 | 100 | Soil | Australia |
| SRR3453389 | SRX1729235 | PRJNA317932 | SRP074055 | 99.23 | Soil | Australia |
| SRR3453391 | SRX1729237 | PRJNA317932 | SRP074055 | 99.24 | Soil | Australia |
| SRR3453398 | SRX1729244 | PRJNA317932 | SRP074055 | 100 | Soil | Australia |
| SRR3453401 | SRX1729247 | PRJNA317932 | SRP074055 | 99.23 | Soil | Australia |
| SRR3453407 | SRX1729253 | PRJNA317932 | SRP074055 | 100 | Soil | Australia |
| SRR3453409 | SRX1729255 | PRJNA317932 | SRP074055 | 99.23 | Soil | Australia |
| SRR3453856 | SRX1729702 | PRJNA317932 | SRP074055 | 100 | Soil | Australia |
| SRR3454006 | SRX1729852 | PRJNA317932 | SRP074055 | 99.23 | Soil | Australia |
| SRR3454045 | SRX1729891 | PRJNA317932 | SRP074055 | 99.23 | Soil | Australia |
| SRR3454051 | SRX1729897 | PRJNA317932 | SRP074055 | 100 | Soil | Australia |
| SRR3454055 | SRX1729901 | PRJNA317932 | SRP074055 | 100 | Soil | Australia |
| SRR3454214 | SRX1730060 | PRJNA317932 | SRP074055 | 99.23 | Soil | Australia |
| SRR3455093 | SRX1730939 | PRJNA317932 | SRP074055 | 100 | Soil | Australia |
| SRR3455102 | SRX1730948 | PRJNA317932 | SRP074055 | 99.23 | Soil | Australia |
| SRR3455700 | SRX1731546 | PRJNA317932 | SRP074055 | 99.23 | Soil | Australia |
| SRR3455704 | SRX1731550 | PRJNA317932 | SRP074055 | 100 | Soil | Australia |
| SRR3455722 | SRX1731568 | PRJNA317932 | SRP074055 | 100 | Soil | Australia |
| SRR3455726 | SRX1731572 | PRJNA317932 | SRP074055 | 100 | Soil | Australia |
| SRR3455772 | SRX1731618 | PRJNA317932 | SRP074055 | 99.23 | Soil | Australia |
| SRR3455779 | SRX1731625 | PRJNA317932 | SRP074055 | 100 | Soil | Australia |
| SRR3455785 | SRX1731631 | PRJNA317932 | SRP074055 | 100 | Soil | Australia |
| SRR3456236 | SRX1732082 | PRJNA317932 | SRP074055 | 99.23 | Soil | Australia |
| SRR3456248 | SRX1732094 | PRJNA317932 | SRP074055 | 99.23 | Soil | Australia |
| SRR3456252 | SRX1732098 | PRJNA317932 | SRP074055 | 99.23 | Soil | Australia |
| SRR3456281 | SRX1732127 | PRJNA317932 | SRP074055 | 99.23 | Soil | Australia |
| SRR5439206 | SRX2729110 | PRJNA382697 | SRP103693 | 100 | Soil | Australia |
| SRR5712814 | SRX2933271 | PRJNA390436 | SRP109688 | 99.29 | Rhizosphere | Australia |
| SRR5712813 | SRX2933272 | PRJNA390436 | SRP109688 | 100 | Soil | Australia |
| SRR5712810 | SRX2933275 | PRJNA390436 | SRP109688 | 100 | Soil | Australia |
| SRR5713849 | SRX2933949 | PRJNA390436 | SRP109688 | 99.64 | Soil | Australia |
| SRR5713842 | SRX2933956 | PRJNA390436 | SRP109688 | 99.22 | Soil | Australia |
| SRR5713760 | SRX2934038 | PRJNA390436 | SRP109688 | 99.23 | Soil | Australia |
| SRR5713660 | SRX2934138 | PRJNA390436 | SRP109688 | 99.23 | Rhizosphere | Australia |
| SRR5713875 | SRX2934249 | PRJNA390436 | SRP109688 | 99.61 | Rhizosphere | Australia |
| SRR5895828 | SRX3061205 | PRJNA317932 | SRP074055 | 99.67 | Soil | Australia |
| SRR5895676 | SRX3061357 | PRJNA317932 | SRP074055 | 100 | Soil | Australia |
| SRR5916398 | SRX3077033 | PRJNA317932 | SRP074055 | 99.23 | Soil | Australia |
| SRR5922951 | SRX3083753 | PRJNA317932 | SRP074055 | 100 | Soil | Australia |
| SRR7365012 | SRX4237868 | PRJNA476765 | SRP150847 | 99.66 | Faeces | Australia |
| SRR8088554 | SRX4915426 | PRJNA497337 | SRP166387 | 99.66 | Tree hollow | Australia |
| SRR8088552 | SRX4915428 | PRJNA497337 | SRP166387 | 100 | Tree hollow | Australia |
| SRR8088551 | SRX4915429 | PRJNA497337 | SRP166387 | 100 | Tree hollow | Australia |
| SRR8088546 | SRX4915434 | PRJNA497337 | SRP166387 | 99.31 | Tree hollow | Australia |
| SRR8088541 | SRX4915439 | PRJNA497337 | SRP166387 | 99.32 | Tree hollow | Australia |
| SRR8088536 | SRX4915444 | PRJNA497337 | SRP166387 | 99.66 | Tree hollow | Australia |
| SRR8088534 | SRX4915446 | PRJNA497337 | SRP166387 | 99.66 | Tree hollow | Australia |
| SRR8821165 | SRX5609583 | PRJNA317932 | SRP074055 | 100 | Soil | Australia |
| SRR8821583 | SRX5609981 | PRJNA317932 | SRP074055 | 100 | Soil | Australia |
| SRR8821807 | SRX5610069 | PRJNA317932 | SRP074055 | 99.23 | Soil | Australia |
| SRR8821603 | SRX5610273 | PRJNA317932 | SRP074055 | 100 | Soil | Australia |
| SRR8821601 | SRX5610275 | PRJNA317932 | SRP074055 | 99.23 | Soil | Australia |
| SRR8822085 | SRX5610319 | PRJNA317932 | SRP074055 | 99.23 | Soil | Australia |
| SRR8822078 | SRX5610326 | PRJNA317932 | SRP074055 | 99.23 | Soil | Australia |
| SRR8822071 | SRX5610333 | PRJNA317932 | SRP074055 | 100 | Soil | Australia |
| SRR8821992 | SRX5610412 | PRJNA317932 | SRP074055 | 99.23 | Soil | Australia |
| SRR8821922 | SRX5610482 | PRJNA317932 | SRP074055 | 100 | Soil | Australia |
| SRR8821915 | SRX5610489 | PRJNA317932 | SRP074055 | 99.23 | Soil | Australia |
| SRR8821888 | SRX5610516 | PRJNA317932 | SRP074055 | 100 | Soil | Australia |
| SRR8822291 | SRX5610612 | PRJNA317932 | SRP074055 | 100 | Soil | Australia |
| SRR8822283 | SRX5610620 | PRJNA317932 | SRP074055 | 100 | Soil | Australia |
| SRR8822219 | SRX5610684 | PRJNA317932 | SRP074055 | 100 | Soil | Australia |
| SRR8822211 | SRX5610692 | PRJNA317932 | SRP074055 | 100 | Soil | Australia |
| SRR8822202 | SRX5610701 | PRJNA317932 | SRP074055 | 99.23 | Soil | Australia |
| SRR8822198 | SRX5610705 | PRJNA317932 | SRP074055 | 100 | Soil | Australia |
| SRR8822159 | SRX5610744 | PRJNA317932 | SRP074055 | 100 | Soil | Australia |
| SRR8822146 | SRX5610757 | PRJNA317932 | SRP074055 | 100 | Soil | Australia |
| SRR8822137 | SRX5610766 | PRJNA317932 | SRP074055 | 100 | Soil | Australia |
| SRR1502395 | SRX642350 | PRJNA252425 | SRP043706 | 99.23 | Soil | Australia |
| SRR10613809 | SRX7292775 | PRJNA594469 | SRP235327 | 100 | Soil | Australia |
| SRR10613775 | SRX7292810 | PRJNA594469 | SRP235327 | 100 | Soil | Australia |
| SRR10613774 | SRX7292811 | PRJNA594469 | SRP235327 | 100 | Soil | Australia |
| SRR10613773 | SRX7292812 | PRJNA594469 | SRP235327 | 100 | Soil | Australia |
| SRR10613771 | SRX7292814 | PRJNA594469 | SRP235327 | 99.66 | Soil | Australia |
| SRR10613770 | SRX7292815 | PRJNA594469 | SRP235327 | 99.66 | Soil | Australia |
| SRR10613767 | SRX7292818 | PRJNA594469 | SRP235327 | 100 | Soil | Australia |
| SRR10613765 | SRX7292820 | PRJNA594469 | SRP235327 | 99.66 | Soil | Australia |
| SRR10613764 | SRX7292821 | PRJNA594469 | SRP235327 | 99.66 | Soil | Australia |
| SRR10613763 | SRX7292822 | PRJNA594469 | SRP235327 | 100 | Soil | Australia |
| SRR10613762 | SRX7292823 | PRJNA594469 | SRP235327 | 100 | Soil | Australia |
| SRR10613761 | SRX7292824 | PRJNA594469 | SRP235327 | 99.66 | Soil | Australia |
| SRR10613760 | SRX7292825 | PRJNA594469 | SRP235327 | 100 | Soil | Australia |
| SRR10613758 | SRX7292827 | PRJNA594469 | SRP235327 | 99.66 | Soil | Australia |
| SRR10613757 | SRX7292828 | PRJNA594469 | SRP235327 | 100 | Soil | Australia |
| SRR10613739 | SRX7292846 | PRJNA594469 | SRP235327 | 100 | Soil | Australia |
| SRR10613738 | SRX7292847 | PRJNA594469 | SRP235327 | 100 | Soil | Australia |
| SRR10613736 | SRX7292849 | PRJNA594469 | SRP235327 | 99.66 | Soil | Australia |
| SRR10613734 | SRX7292851 | PRJNA594469 | SRP235327 | 99.66 | Soil | Australia |
| SRR10613731 | SRX7292854 | PRJNA594469 | SRP235327 | 99.66 | Soil | Australia |
| SRR10613730 | SRX7292855 | PRJNA594469 | SRP235327 | 100 | Soil | Australia |
| SRR10613718 | SRX7292867 | PRJNA594469 | SRP235327 | 99.66 | Soil | Australia |
| SRR10613714 | SRX7292871 | PRJNA594469 | SRP235327 | 99.66 | Soil | Australia |
| SRR10613713 | SRX7292872 | PRJNA594469 | SRP235327 | 99.66 | Soil | Australia |
| SRR10613707 | SRX7292878 | PRJNA594469 | SRP235327 | 99.31 | Soil | Australia |
| SRR10613706 | SRX7292879 | PRJNA594469 | SRP235327 | 99.66 | Soil | Australia |
| SRR10613651 | SRX7292933 | PRJNA594469 | SRP235327 | 100 | Soil | Australia |
| SRR10613640 | SRX7292944 | PRJNA594469 | SRP235327 | 99.66 | Soil | Australia |
| SRR10613622 | SRX7292962 | PRJNA594469 | SRP235327 | 100 | Soil | Australia |
| SRR11575064 | SRX8143027 | PRJNA626608 | SRP257528 | 100 | Soil | Australia |
| SRR11575052 | SRX8143039 | PRJNA626608 | SRP257528 | 100 | Soil | Australia |
| SRR11575037 | SRX8143054 | PRJNA626608 | SRP257528 | 100 | Soil | Australia |
| SRR11575035 | SRX8143056 | PRJNA626608 | SRP257528 | 100 | Soil | Australia |
| SRR11575033 | SRX8143058 | PRJNA626608 | SRP257528 | 100 | Soil | Australia |
| SRR11575031 | SRX8143060 | PRJNA626608 | SRP257528 | 100 | Soil | Australia |
| SRR11575028 | SRX8143063 | PRJNA626608 | SRP257528 | 99.66 | Soil | Australia |
| SRR11575025 | SRX8143066 | PRJNA626608 | SRP257528 | 100 | Soil | Australia |
| SRR11575023 | SRX8143068 | PRJNA626608 | SRP257528 | 99.32 | Soil | Australia |
| SRR11575022 | SRX8143069 | PRJNA626608 | SRP257528 | 100 | Soil | Australia |
| SRR11575021 | SRX8143070 | PRJNA626608 | SRP257528 | 99.66 | Soil | Australia |
| SRR11575020 | SRX8143071 | PRJNA626608 | SRP257528 | 99.32 | Soil | Australia |
| SRR11575018 | SRX8143073 | PRJNA626608 | SRP257528 | 99.66 | Soil | Australia |
| SRR11575017 | SRX8143074 | PRJNA626608 | SRP257528 | 99.66 | Soil | Australia |
| SRR11575016 | SRX8143075 | PRJNA626608 | SRP257528 | 99.66 | Soil | Australia |
| SRR11575015 | SRX8143076 | PRJNA626608 | SRP257528 | 99.66 | Soil | Australia |
| SRR11575013 | SRX8143078 | PRJNA626608 | SRP257528 | 99.66 | Soil | Australia |
| SRR11575012 | SRX8143079 | PRJNA626608 | SRP257528 | 99.66 | Soil | Australia |
| SRR11575009 | SRX8143082 | PRJNA626608 | SRP257528 | 99.66 | Soil | Australia |
| SRR11575007 | SRX8143084 | PRJNA626608 | SRP257528 | 100 | Soil | Australia |
| SRR11575006 | SRX8143085 | PRJNA626608 | SRP257528 | 100 | Soil | Australia |
| SRR11575004 | SRX8143087 | PRJNA626608 | SRP257528 | 99.66 | Soil | Australia |
| SRR11575002 | SRX8143089 | PRJNA626608 | SRP257528 | 99.66 | Soil | Australia |
| SRR11574999 | SRX8143092 | PRJNA626608 | SRP257528 | 99.66 | Soil | Australia |
| SRR11574998 | SRX8143093 | PRJNA626608 | SRP257528 | 100 | Soil | Australia |
| SRR11574987 | SRX8143104 | PRJNA626608 | SRP257528 | 100 | Soil | Australia |
| SRR11574982 | SRX8143109 | PRJNA626608 | SRP257528 | 100 | Soil | Australia |
| SRR11574960 | SRX8143131 | PRJNA626608 | SRP257528 | 99.66 | Soil | Australia |
| SRR11574927 | SRX8143164 | PRJNA626608 | SRP257528 | 100 | Soil | Australia |
| SRR11574916 | SRX8143175 | PRJNA626608 | SRP257528 | 100 | Soil | Australia |
| ERR2818242 | ERX2825049 | PRJEB28861 | ERP111119 | 99.32 | Rhizosphere | Austria |
| ERR2818245 | ERX2825052 | PRJEB28861 | ERP111119 | 99.32 | Rhizosphere | Austria |
| SRR9701021 | SRX6458857 | PRJNA551019 | SRP215159 | 99.61 | Soil | Austria |
| SRR9700993 | SRX6458885 | PRJNA551019 | SRP215159 | 99.26 | Soil | Austria |
| SRR9700943 | SRX6458935 | PRJNA551019 | SRP215159 | 99.61 | Soil | Austria |
| SRR9700942 | SRX6458936 | PRJNA551019 | SRP215159 | 99.62 | Soil | Austria |
| SRR9700880 | SRX6458998 | PRJNA551019 | SRP215159 | 99.61 | Soil | Austria |
| SRR9700879 | SRX6458999 | PRJNA551019 | SRP215159 | 99.61 | Soil | Austria |
| SRR9700854 | SRX6459024 | PRJNA551019 | SRP215159 | 99.62 | Soil | Austria |
| ERR2849759 | ERX2856411 | PRJEB29246 | ERP111535 | 99 | Soil | Belgium |
| ERR2849772 | ERX2856424 | PRJEB29246 | ERP111535 | 99 | Soil | Belgium |
| ERR2849774 | ERX2856426 | PRJEB29246 | ERP111535 | 100 | Soil | Belgium |
| SRR10242770 | SRX6961230 | PRJNA576339 | SRP224703 | 100 | Soil | Belgium |
| SRR10242766 | SRX6961234 | PRJNA576339 | SRP224703 | 100 | Soil | Belgium |
| SRR10242744 | SRX6961256 | PRJNA576339 | SRP224703 | 99.67 | Soil | Belgium |
| SRR10242731 | SRX6961269 | PRJNA576339 | SRP224703 | 99.67 | Soil | Belgium |
| SRR10242682 | SRX6961318 | PRJNA576339 | SRP224703 | 100 | Rhizosphere | Belgium |
| SRR10958975 | SRX7625077 | PRJNA602824 | SRP244431 | 100 | Soil | Belgium |
| SRR11522842 | SRX8093908 | PRJNA624053 | SRP255941 | 99.67 | Compost | Belgium |
| SRR11522836 | SRX8093914 | PRJNA624053 | SRP255941 | 99.67 | Compost | Belgium |
| SRR11522828 | SRX8093922 | PRJNA624053 | SRP255941 | 100 | Compost | Belgium |
| SRR11522802 | SRX8093948 | PRJNA624053 | SRP255941 | 99.67 | Compost | Belgium |
| SRR11522795 | SRX8093955 | PRJNA624053 | SRP255941 | 100 | Compost | Belgium |
| SRR11522791 | SRX8093959 | PRJNA624053 | SRP255941 | 100 | Compost | Belgium |
| SRR11522783 | SRX8093967 | PRJNA624053 | SRP255941 | 99 | Compost | Belgium |
| SRR11522775 | SRX8093975 | PRJNA624053 | SRP255941 | 100 | Spent growing medium | Belgium |
| SRR11522772 | SRX8093978 | PRJNA624053 | SRP255941 | 100 | Spent growing medium | Belgium |
| SRR11522771 | SRX8093979 | PRJNA624053 | SRP255941 | 99 | Spent growing medium | Belgium |
| SRR11522749 | SRX8094001 | PRJNA624053 | SRP255941 | 100 | Spent growing medium | Belgium |
| SRR11522748 | SRX8094002 | PRJNA624053 | SRP255941 | 100 | Spent growing medium | Belgium |
| SRR11522747 | SRX8094003 | PRJNA624053 | SRP255941 | 100 | Spent growing medium | Belgium |
| SRR11522745 | SRX8094005 | PRJNA624053 | SRP255941 | 100 | Spent growing medium | Belgium |
| SRR11522740 | SRX8094010 | PRJNA624053 | SRP255941 | 99.67 | Spent growing medium | Belgium |
| SRR11522736 | SRX8094014 | PRJNA624053 | SRP255941 | 99.67 | Spent growing medium | Belgium |
| SRR11522729 | SRX8094021 | PRJNA624053 | SRP255941 | 100 | Spent growing medium | Belgium |
| SRR11522728 | SRX8094022 | PRJNA624053 | SRP255941 | 100 | Spent growing medium | Belgium |
| SRR11522727 | SRX8094023 | PRJNA624053 | SRP255941 | 100 | Spent growing medium | Belgium |
| SRR11522726 | SRX8094024 | PRJNA624053 | SRP255941 | 100 | Spent growing medium | Belgium |
| SRR11522725 | SRX8094025 | PRJNA624053 | SRP255941 | 100 | Spent growing medium | Belgium |
| SRR11522724 | SRX8094026 | PRJNA624053 | SRP255941 | 100 | Spent growing medium | Belgium |
| SRR11522719 | SRX8094031 | PRJNA624053 | SRP255941 | 99.67 | Spent growing medium | Belgium |
| SRR11522701 | SRX8094049 | PRJNA624053 | SRP255941 | 100 | Spent growing medium | Belgium |
| SRR11522681 | SRX8094069 | PRJNA624053 | SRP255941 | 100 | Spent growing medium | Belgium |
| SRR8665400 | SRX5461465 | PRJNA524365 | SRP187451 | 100 | Soil | Brazil |
| SRR8665261 | SRX5461604 | PRJNA524365 | SRP187451 | 100 | Soil | Brazil |
| SRR8665732 | SRX5461736 | PRJNA524365 | SRP187451 | 100 | Soil | Brazil |
| SRR8665731 | SRX5461737 | PRJNA524365 | SRP187451 | 99.2 | Soil | Brazil |
| SRR8665726 | SRX5461742 | PRJNA524365 | SRP187451 | 99.6 | Soil | Brazil |
| SRR8665532 | SRX5461936 | PRJNA524365 | SRP187451 | 99.2 | Soil | Brazil |
| SRR8665498 | SRX5461970 | PRJNA524365 | SRP187451 | 99.2 | Soil | Brazil |
| SRR8665487 | SRX5461981 | PRJNA524365 | SRP187451 | 99.2 | Soil | Brazil |
| SRR8665959 | SRX5462036 | PRJNA524365 | SRP187451 | 100 | Soil | Brazil |
| SRR8665942 | SRX5462053 | PRJNA524365 | SRP187451 | 100 | Soil | Brazil |
| SRR8665816 | SRX5462179 | PRJNA524365 | SRP187451 | 99.2 | Soil | Brazil |
| SRR8665815 | SRX5462180 | PRJNA524365 | SRP187451 | 99.2 | Soil | Brazil |
| SRR11529087 | SRX8100133 | PRJNA624777 | SRP256066 | 99.2 | Soil | Brazil |
| SRR11529086 | SRX8100134 | PRJNA624777 | SRP256066 | 100 | Soil | Brazil |
| SRR11529085 | SRX8100135 | PRJNA624777 | SRP256066 | 100 | Soil | Brazil |
| SRR11529082 | SRX8100138 | PRJNA624777 | SRP256066 | 100 | Soil | Brazil |
| SRR11529081 | SRX8100139 | PRJNA624777 | SRP256066 | 99.6 | Soil | Brazil |
| SRR11529080 | SRX8100140 | PRJNA624777 | SRP256066 | 99.6 | Soil | Brazil |
| SRR11529079 | SRX8100141 | PRJNA624777 | SRP256066 | 99.6 | Soil | Brazil |
| SRR11529078 | SRX8100142 | PRJNA624777 | SRP256066 | 100 | Soil | Brazil |
| SRR11529077 | SRX8100143 | PRJNA624777 | SRP256066 | 100 | Soil | Brazil |
| SRR5597622 | SRX2851762 | PRJNA386328 | SRP107966 | 99.64 | Rumen | Canada |
| SRR5597621 | SRX2851763 | PRJNA386328 | SRP107966 | 99.64 | Rumen | Canada |
| SRR5597619 | SRX2851765 | PRJNA386328 | SRP107966 | 99.64 | Rumen | Canada |
| SRR5597618 | SRX2851766 | PRJNA386328 | SRP107966 | 99.64 | Rumen | Canada |
| SRR5597617 | SRX2851767 | PRJNA386328 | SRP107966 | 99.64 | Rumen | Canada |
| SRR5597616 | SRX2851768 | PRJNA386328 | SRP107966 | 99.64 | Rumen | Canada |
| SRR5597615 | SRX2851769 | PRJNA386328 | SRP107966 | 99.64 | Rumen | Canada |
| SRR5597614 | SRX2851770 | PRJNA386328 | SRP107966 | 99.64 | Rumen | Canada |
| SRR5597613 | SRX2851771 | PRJNA386328 | SRP107966 | 99.64 | Rumen | Canada |
| SRR5597562 | SRX2851822 | PRJNA386328 | SRP107966 | 99.64 | Rumen | Canada |
| SRR5597528 | SRX2851856 | PRJNA386328 | SRP107966 | 100 | Rumen | Canada |
| SRR5597526 | SRX2851858 | PRJNA386328 | SRP107966 | 100 | Rumen | Canada |
| SRR5597523 | SRX2851861 | PRJNA386328 | SRP107966 | 100 | Rumen | Canada |
| SRR5597518 | SRX2851866 | PRJNA386328 | SRP107966 | 99.64 | Rumen | Canada |
| SRR5597501 | SRX2851883 | PRJNA386328 | SRP107966 | 99.64 | Rumen | Canada |
| SRR5597500 | SRX2851884 | PRJNA386328 | SRP107966 | 99.64 | Rumen | Canada |
| SRR5597489 | SRX2851895 | PRJNA386328 | SRP107966 | 99.64 | Rumen | Canada |
| SRR5597488 | SRX2851896 | PRJNA386328 | SRP107966 | 99.64 | Rumen | Canada |
| SRR5597484 | SRX2851900 | PRJNA386328 | SRP107966 | 99.64 | Rumen | Canada |
| SRR5597483 | SRX2851901 | PRJNA386328 | SRP107966 | 99.64 | Rumen | Canada |
| SRR5597482 | SRX2851902 | PRJNA386328 | SRP107966 | 99.64 | Rumen | Canada |
| SRR5597476 | SRX2851908 | PRJNA386328 | SRP107966 | 99.64 | Rumen | Canada |
| SRR5597472 | SRX2851912 | PRJNA386328 | SRP107966 | 99.28 | Rumen | Canada |
| SRR5597450 | SRX2851934 | PRJNA386328 | SRP107966 | 99.64 | Rumen | Canada |
| SRR5597446 | SRX2851938 | PRJNA386328 | SRP107966 | 99.64 | Rumen | Canada |
| SRR5597444 | SRX2851940 | PRJNA386328 | SRP107966 | 99.64 | Rumen | Canada |
| SRR5597443 | SRX2851941 | PRJNA386328 | SRP107966 | 100 | Rumen | Canada |
| SRR5597442 | SRX2851942 | PRJNA386328 | SRP107966 | 99.64 | Rumen | Canada |
| SRR5597441 | SRX2851943 | PRJNA386328 | SRP107966 | 99.64 | Rumen | Canada |
| SRR5597439 | SRX2851945 | PRJNA386328 | SRP107966 | 100 | Rumen | Canada |
| SRR7262748 | SRX4166761 | PRJNA448939 | SRP137769 | 99.71 | Bovine milk | Canada |
| SRR7262623 | SRX4166886 | PRJNA448939 | SRP137769 | 100 | Bovine milk | Canada |
| SRR8292068 | SRX5106558 | PRJNA384633 | SRP172888 | 99.2 | Soil | Canada |
| SRR8292113 | SRX5106700 | PRJNA384633 | SRP172888 | 99.2 | Soil | Canada |
| SRR8292106 | SRX5106707 | PRJNA384633 | SRP172888 | 99.2 | Soil | Canada |
| SRR8959479 | SRX5738954 | PRJNA536254 | SRP193899 | 100 | Human milk | Canada |
| SRR8959476 | SRX5738957 | PRJNA536254 | SRP193899 | 100 | Human milk | Canada |
| SRR8959473 | SRX5738960 | PRJNA536254 | SRP193899 | 100 | Human milk | Canada |
| SRR10259515 | SRX6977318 | PRJNA575258 | SRP223914 | 100 | Soil | Canada |
| SRR11230689 | SRX7842685 | PRJNA609777 | SRP251316 | 99.2 | Soil | Canada |
| SRR11595111 | SRX8161882 | PRJNA627407 | SRP258049 | 99.67 | Soil | Canada |
| SRR11595110 | SRX8161883 | PRJNA627407 | SRP258049 | 99.67 | Soil | Canada |
| SRR11595103 | SRX8161890 | PRJNA627407 | SRP258049 | 100 | Soil | Canada |
| SRR11595088 | SRX8161905 | PRJNA627407 | SRP258049 | 100 | Soil | Canada |
| SRR11595083 | SRX8161910 | PRJNA627407 | SRP258049 | 100 | Soil | Canada |
| SRR11595076 | SRX8161917 | PRJNA627407 | SRP258049 | 100 | Soil | Canada |
| SRR11595075 | SRX8161918 | PRJNA627407 | SRP258049 | 100 | Soil | Canada |
| SRR11595074 | SRX8161919 | PRJNA627407 | SRP258049 | 100 | Soil | Canada |
| SRR11595067 | SRX8161926 | PRJNA627407 | SRP258049 | 100 | Soil | Canada |
| SRR11595062 | SRX8161931 | PRJNA627407 | SRP258049 | 100 | Soil | Canada |
| SRR11595061 | SRX8161932 | PRJNA627407 | SRP258049 | 100 | Soil | Canada |
| SRR11595060 | SRX8161933 | PRJNA627407 | SRP258049 | 100 | Soil | Canada |
| SRR11595047 | SRX8161946 | PRJNA627407 | SRP258049 | 99.67 | Soil | Canada |
| SRR11595041 | SRX8161952 | PRJNA627407 | SRP258049 | 100 | Soil | Canada |
| SRR11595040 | SRX8161953 | PRJNA627407 | SRP258049 | 99.67 | Soil | Canada |
| SRR11595035 | SRX8161958 | PRJNA627407 | SRP258049 | 100 | Soil | Canada |
| SRR11595033 | SRX8161960 | PRJNA627407 | SRP258049 | 100 | Soil | Canada |
| SRR11595026 | SRX8161967 | PRJNA627407 | SRP258049 | 100 | Soil | Canada |
| SRR11595025 | SRX8161968 | PRJNA627407 | SRP258049 | 100 | Soil | Canada |
| SRR11595020 | SRX8161973 | PRJNA627407 | SRP258049 | 100 | Soil | Canada |
| SRR11595018 | SRX8161975 | PRJNA627407 | SRP258049 | 100 | Soil | Canada |
| SRR11595012 | SRX8161981 | PRJNA627407 | SRP258049 | 100 | Soil | Canada |
| SRR11595009 | SRX8161984 | PRJNA627407 | SRP258049 | 100 | Soil | Canada |
| SRR11595004 | SRX8161989 | PRJNA627407 | SRP258049 | 100 | Soil | Canada |
| SRR11838315 | SRX8388710 | PRJNA634744 | SRP263161 | 99.2 | Soil | Canada |
| SRR11838313 | SRX8388712 | PRJNA634744 | SRP263161 | 99.2 | Soil | Canada |
| SRR11838311 | SRX8388714 | PRJNA634744 | SRP263161 | 99.2 | Soil | Canada |
| SRR11838293 | SRX8388732 | PRJNA634744 | SRP263161 | 99.2 | Soil | Canada |
| SRR11838288 | SRX8388737 | PRJNA634744 | SRP263161 | 99.6 | Soil | Canada |
| SRR12039723 | SRX8569463 | PRJNA639578 | SRP267699 | 99.64 | Soil | Canada |
| SRR12039717 | SRX8569469 | PRJNA639578 | SRP267699 | 99.64 | Soil | Canada |
| SRR12039716 | SRX8569470 | PRJNA639578 | SRP267699 | 99.29 | Soil | Canada |
| SRR12039715 | SRX8569471 | PRJNA639578 | SRP267699 | 99.29 | Soil | Canada |
| SRR12039714 | SRX8569472 | PRJNA639578 | SRP267699 | 99.64 | Soil | Canada |
| SRR12039712 | SRX8569474 | PRJNA639578 | SRP267699 | 99.64 | Soil | Canada |
| SRR12039704 | SRX8569482 | PRJNA639578 | SRP267699 | 99.64 | Soil | Canada |
| SRR12039697 | SRX8569489 | PRJNA639578 | SRP267699 | 99.64 | Soil | Canada |
| SRR12039688 | SRX8569498 | PRJNA639578 | SRP267699 | 99.29 | Soil | Canada |
| SRR12039682 | SRX8569504 | PRJNA639578 | SRP267699 | 99.64 | Soil | Canada |
| ERR1120304 | ERX1199734 | PRJEB8978 | ERP010027 | 99.18 | Wood | China |
| ERR1292966 | ERX1364572 | PRJEB12829 | ERP014348 | 99.66 | Soil | China |
| ERR1292978 | ERX1364584 | PRJEB12829 | ERP014348 | 99.32 | Soil | China |
| ERR1292980 | ERX1364586 | PRJEB12829 | ERP014348 | 99.66 | Soil | China |
| ERR1292982 | ERX1364588 | PRJEB12829 | ERP014348 | 99.66 | Soil | China |
| ERR1292983 | ERX1364589 | PRJEB12829 | ERP014348 | 99.66 | Soil | China |
| ERR2120070 | ERX2176978 | PRJEB22634 | ERP104329 | 99.63 | Soil | China |
| ERR3021244 | ERX3023427 | PRJEB30523 | ERP112987 | 99.63 | Soil | China |
| ERR3021249 | ERX3023432 | PRJEB30527 | ERP112993 | 99.63 | Soil | China |
| ERR3349256 | ERX3373601 | PRJEB32542 | ERP115241 | 99.18 | Soil | China |
| ERR3349311 | ERX3373656 | PRJEB32542 | ERP115241 | 99.59 | Soil | China |
| ERR3349352 | ERX3373697 | PRJEB32542 | ERP115241 | 99.18 | Soil | China |
| ERR3349375 | ERX3373720 | PRJEB32542 | ERP115241 | 99.59 | Soil | China |
| ERR3349376 | ERX3373721 | PRJEB32542 | ERP115241 | 99.18 | Soil | China |
| ERR3349378 | ERX3373723 | PRJEB32542 | ERP115241 | 99.59 | Soil | China |
| ERR3349379 | ERX3373724 | PRJEB32542 | ERP115241 | 99.59 | Soil | China |
| ERR3349381 | ERX3373726 | PRJEB32542 | ERP115241 | 99.59 | Soil | China |
| ERR3349395 | ERX3373740 | PRJEB32542 | ERP115241 | 99.18 | Soil | China |
| ERR3349423 | ERX3373768 | PRJEB32542 | ERP115241 | 99.59 | Soil | China |
| SRR2189232 | SRX1176940 | PRJNA292998 | SRP062512 | 99.6 | Soil | China |
| SRR2226499 | SRX1177166 | PRJNA294253 | SRP062990 | 99.66 | Soil | China |
| SRR2226503 | SRX1177170 | PRJNA294253 | SRP062990 | 99.66 | Soil | China |
| SRR2226504 | SRX1177171 | PRJNA294253 | SRP062990 | 99.66 | Soil | China |
| SRR3087209 | SRX1518807 | PRJNA276163 | SRP055455 | 100 | Soil | China |
| SRR3344414 | SRX1685515 | PRJNA317607 | SRP072977 | 99.63 | Sewage sludge | China |
| SRR3485673 | SRX1747502 | PRJNA320317 | SRP074542 | 100 | Freshwater | China |
| SRR3509079 | SRX1763196 | PRJNA321647 | SRP075187 | 99.58 | Soil | China |
| SRR3509085 | SRX1763202 | PRJNA321647 | SRP075187 | 99.58 | Soil | China |
| SRR3509086 | SRX1763203 | PRJNA321647 | SRP075187 | 99.58 | Soil | China |
| SRR3509161 | SRX1763205 | PRJNA321647 | SRP075187 | 99.58 | Soil | China |
| SRR3509290 | SRX1763208 | PRJNA321647 | SRP075187 | 99.58 | Soil | China |
| SRR3509291 | SRX1763209 | PRJNA321647 | SRP075187 | 99.58 | Soil | China |
| SRR3674596 | SRX1850186 | PRJNA320663 | SRP076655 | 99.18 | Soil | China |
| SRR4290488 | SRX2185994 | PRJNA343415 | SRP090114 | 99.66 | Soil | China |
| SRR4290489 | SRX2185995 | PRJNA343415 | SRP090114 | 100 | Soil | China |
| SRR4290490 | SRX2185996 | PRJNA343415 | SRP090114 | 100 | Soil | China |
| SRR4290491 | SRX2185997 | PRJNA343415 | SRP090114 | 99.66 | Soil | China |
| SRR4290492 | SRX2185998 | PRJNA343415 | SRP090114 | 99.66 | Soil | China |
| SRR4290495 | SRX2186001 | PRJNA343415 | SRP090114 | 99.66 | Soil | China |
| SRR4290497 | SRX2186003 | PRJNA343415 | SRP090114 | 99.66 | Soil | China |
| SRR4290498 | SRX2186004 | PRJNA343415 | SRP090114 | 99.66 | Soil | China |
| SRR4290499 | SRX2186005 | PRJNA343415 | SRP090114 | 99.66 | Soil | China |
| SRR4290501 | SRX2186007 | PRJNA343415 | SRP090114 | 99.66 | Soil | China |
| SRR4290502 | SRX2186008 | PRJNA343415 | SRP090114 | 99.66 | Soil | China |
| SRR4290503 | SRX2186009 | PRJNA343415 | SRP090114 | 99.66 | Soil | China |
| SRR4420123 | SRX2242238 | PRJNA347605 | SRP091476 | 99.59 | Soil | China |
| SRR4420124 | SRX2242239 | PRJNA347605 | SRP091476 | 99.18 | Soil | China |
| SRR4420126 | SRX2242241 | PRJNA347605 | SRP091476 | 99.59 | Soil | China |
| SRR4420128 | SRX2242243 | PRJNA347605 | SRP091476 | 99.59 | Soil | China |
| SRR4420129 | SRX2242315 | PRJNA347605 | SRP091476 | 99.19 | Soil | China |
| SRR4963381 | SRX2328874 | PRJNA352590 | SRP092634 | 99.61 | Dust | China |
| SRR5025856 | SRX2352145 | PRJNA353565 | SRP093408 | 99.2 | Soil | China |
| SRR5025866 | SRX2352155 | PRJNA353565 | SRP093408 | 99.17 | Soil | China |
| SRR5025867 | SRX2352156 | PRJNA353565 | SRP093408 | 99.2 | Soil | China |
| SRR5131258 | SRX2445081 | PRJNA358801 | SRP095662 | 99.22 | Sediment | China |
| SRR5224516 | SRX2533695 | PRJNA352102 | SRP092656 | 99.19 | Soil | China |
| SRR5224517 | SRX2533696 | PRJNA352102 | SRP092656 | 99.17 | Soil | China |
| SRR5224519 | SRX2533698 | PRJNA352102 | SRP092656 | 99.66 | Soil | China |
| SRR5224521 | SRX2533700 | PRJNA352102 | SRP092656 | 99.59 | Soil | China |
| SRR5224537 | SRX2533716 | PRJNA352102 | SRP092656 | 99.59 | Soil | China |
| SRR5319902 | SRX2619310 | PRJNA378304 | SRP101536 | 99.32 | Sewage sludge | China |
| SRR5319903 | SRX2619311 | PRJNA378304 | SRP101536 | 99.66 | Sewage sludge | China |
| SRR5319907 | SRX2619315 | PRJNA378304 | SRP101536 | 99.32 | Sewage sludge | China |
| SRR5319909 | SRX2619317 | PRJNA378304 | SRP101536 | 99.66 | Sewage sludge | China |
| SRR5319910 | SRX2619318 | PRJNA378304 | SRP101536 | 100 | Sewage sludge | China |
| SRR5319928 | SRX2619336 | PRJNA378304 | SRP101536 | 99.32 | Sewage sludge | China |
| SRR5319929 | SRX2619337 | PRJNA378304 | SRP101536 | 99.31 | Sewage sludge | China |
| SRR5319931 | SRX2619339 | PRJNA378304 | SRP101536 | 99.66 | Sewage sludge | China |
| SRR5328922 | SRX2627942 | PRJNA378603 | SRP101619 | 99.18 | Anaerobic reactor | China |
| SRR5383640 | SRX2678696 | PRJNA380834 | SRP102634 | 99.61 | Soil | China |
| SRR5397262 | SRX2692055 | PRJNA380157 | SRP102761 | 99.59 | Soil | China |
| SRR5397279 | SRX2692072 | PRJNA380157 | SRP102761 | 100 | Soil | China |
| SRR5397290 | SRX2692083 | PRJNA380157 | SRP102761 | 99.2 | Soil | China |
| SRR5404571 | SRX2697291 | PRJNA381253 | SRP102860 | 99.6 | Soil | China |
| SRR5412133 | SRX2704180 | PRJNA381437 | SRP102981 | 99.6 | Soil | China |
| SRR5714592 | SRX2934940 | PRJNA391050 | SRP109775 | 99.58 | Soil | China |
| SRR5714585 | SRX2934947 | PRJNA391050 | SRP109775 | 99.58 | Soil | China |
| SRR5714576 | SRX2934956 | PRJNA391050 | SRP109775 | 99.58 | Soil | China |
| SRR5714568 | SRX2934964 | PRJNA391050 | SRP109775 | 99.58 | Soil | China |
| SRR5714565 | SRX2934967 | PRJNA391050 | SRP109775 | 99.58 | Soil | China |
| SRR5786197 | SRX2972662 | PRJNA392358 | SRP110777 | 100 | Rhizosphere | China |
| SRR5837439 | SRX3013907 | PRJNA393952 | SRP112719 | 100 | Soil | China |
| SRR5837406 | SRX3013940 | PRJNA393952 | SRP112719 | 99.6 | Soil | China |
| SRR5852015 | SRX3021562 | PRJNA390715 | SRP109345 | 99.32 | Soil | China |
| SRR5852002 | SRX3021575 | PRJNA390715 | SRP109345 | 99.65 | Soil | China |
| SRR5860065 | SRX3028501 | PRJNA395263 | SRP113348 | 100 | Soil | China |
| SRR5860022 | SRX3028544 | PRJNA395263 | SRP113348 | 100 | Soil | China |
| SRR5860020 | SRX3028546 | PRJNA395263 | SRP113348 | 100 | Soil | China |
| SRR5860018 | SRX3028548 | PRJNA395263 | SRP113348 | 100 | Soil | China |
| SRR5859987 | SRX3028579 | PRJNA395263 | SRP113348 | 100 | Soil | China |
| SRR5859984 | SRX3028582 | PRJNA395263 | SRP113348 | 100 | Soil | China |
| SRR5859982 | SRX3028584 | PRJNA395263 | SRP113348 | 100 | Soil | China |
| SRR5859944 | SRX3028622 | PRJNA395263 | SRP113348 | 100 | Soil | China |
| SRR5859888 | SRX3028678 | PRJNA395263 | SRP113348 | 100 | Rhizosphere | China |
| SRR5859887 | SRX3028679 | PRJNA395263 | SRP113348 | 100 | Rhizosphere | China |
| SRR5859868 | SRX3028698 | PRJNA395263 | SRP113348 | 100 | Rhizosphere | China |
| SRR5859858 | SRX3028708 | PRJNA395263 | SRP113348 | 100 | Rhizosphere | China |
| SRR5859817 | SRX3028749 | PRJNA395263 | SRP113348 | 100 | Soil | China |
| SRR5859816 | SRX3028750 | PRJNA395263 | SRP113348 | 100 | Rhizosphere | China |
| SRR5859814 | SRX3028752 | PRJNA395263 | SRP113348 | 100 | Rhizosphere | China |
| SRR5859810 | SRX3028756 | PRJNA395263 | SRP113348 | 99.6 | Soil | China |
| SRR5930275 | SRX3090560 | PRJNA398112 | SRP115338 | 99.59 | Soil | China |
| SRR5992394 | SRX3147837 | PRJNA400517 | SRP116613 | 100 | Soil | China |
| SRR5992391 | SRX3147840 | PRJNA400517 | SRP116613 | 100 | Soil | China |
| SRR5992387 | SRX3147844 | PRJNA400517 | SRP116613 | 99.71 | Soil | China |
| SRR5992385 | SRX3147846 | PRJNA400517 | SRP116613 | 100 | Soil | China |
| SRR5992379 | SRX3147852 | PRJNA400517 | SRP116613 | 100 | Soil | China |
| SRR5992373 | SRX3147858 | PRJNA400517 | SRP116613 | 100 | Soil | China |
| SRR5992367 | SRX3147864 | PRJNA400517 | SRP116613 | 100 | Soil | China |
| SRR5992366 | SRX3147865 | PRJNA400517 | SRP116613 | 100 | Soil | China |
| SRR5992365 | SRX3147866 | PRJNA400517 | SRP116613 | 100 | Soil | China |
| SRR5992364 | SRX3147867 | PRJNA400517 | SRP116613 | 100 | Soil | China |
| SRR5992359 | SRX3147872 | PRJNA400517 | SRP116613 | 100 | Soil | China |
| SRR5992358 | SRX3147873 | PRJNA400517 | SRP116613 | 100 | Soil | China |
| SRR5992355 | SRX3147876 | PRJNA400517 | SRP116613 | 99.71 | Soil | China |
| SRR5992354 | SRX3147877 | PRJNA400517 | SRP116613 | 100 | Soil | China |
| SRR5992352 | SRX3147879 | PRJNA400517 | SRP116613 | 100 | Soil | China |
| SRR5992348 | SRX3147883 | PRJNA400517 | SRP116613 | 100 | Soil | China |
| SRR5992344 | SRX3147887 | PRJNA400517 | SRP116613 | 100 | Soil | China |
| SRR5992328 | SRX3147903 | PRJNA400517 | SRP116613 | 100 | Soil | China |
| SRR5992327 | SRX3147904 | PRJNA400517 | SRP116613 | 100 | Soil | China |
| SRR5992326 | SRX3147905 | PRJNA400517 | SRP116613 | 100 | Soil | China |
| SRR5992316 | SRX3147915 | PRJNA400517 | SRP116613 | 100 | Soil | China |
| SRR5992312 | SRX3147919 | PRJNA400517 | SRP116613 | 100 | Soil | China |
| SRR5992307 | SRX3147924 | PRJNA400517 | SRP116613 | 100 | Soil | China |
| SRR5992304 | SRX3147927 | PRJNA400517 | SRP116613 | 100 | Soil | China |
| SRR5992301 | SRX3147930 | PRJNA400517 | SRP116613 | 100 | Soil | China |
| SRR5992281 | SRX3147950 | PRJNA400517 | SRP116613 | 100 | Soil | China |
| SRR5992261 | SRX3147970 | PRJNA400517 | SRP116613 | 99.71 | Soil | China |
| SRR5992254 | SRX3147977 | PRJNA400517 | SRP116613 | 100 | Soil | China |
| SRR5992252 | SRX3147979 | PRJNA400517 | SRP116613 | 100 | Soil | China |
| SRR5992244 | SRX3147987 | PRJNA400517 | SRP116613 | 100 | Soil | China |
| SRR5992241 | SRX3147990 | PRJNA400517 | SRP116613 | 100 | Soil | China |
| SRR5992239 | SRX3147992 | PRJNA400517 | SRP116613 | 100 | Soil | China |
| SRR5992237 | SRX3147994 | PRJNA400517 | SRP116613 | 100 | Soil | China |
| SRR5992234 | SRX3147997 | PRJNA400517 | SRP116613 | 100 | Soil | China |
| SRR5992233 | SRX3147998 | PRJNA400517 | SRP116613 | 99.29 | Soil | China |
| SRR5992229 | SRX3148002 | PRJNA400517 | SRP116613 | 99.71 | Soil | China |
| SRR5992218 | SRX3148013 | PRJNA400517 | SRP116613 | 100 | Soil | China |
| SRR5992201 | SRX3148030 | PRJNA400517 | SRP116613 | 100 | Soil | China |
| SRR5992193 | SRX3148038 | PRJNA400517 | SRP116613 | 100 | Soil | China |
| SRR5992187 | SRX3148044 | PRJNA400517 | SRP116613 | 100 | Soil | China |
| SRR5992185 | SRX3148046 | PRJNA400517 | SRP116613 | 100 | Soil | China |
| SRR5992183 | SRX3148048 | PRJNA400517 | SRP116613 | 100 | Soil | China |
| SRR5992182 | SRX3148049 | PRJNA400517 | SRP116613 | 100 | Soil | China |
| SRR5992181 | SRX3148050 | PRJNA400517 | SRP116613 | 100 | Soil | China |
| SRR5992178 | SRX3148053 | PRJNA400517 | SRP116613 | 100 | Soil | China |
| SRR5992177 | SRX3148054 | PRJNA400517 | SRP116613 | 100 | Soil | China |
| SRR5992176 | SRX3148055 | PRJNA400517 | SRP116613 | 100 | Soil | China |
| SRR5992175 | SRX3148056 | PRJNA400517 | SRP116613 | 100 | Soil | China |
| SRR5992173 | SRX3148058 | PRJNA400517 | SRP116613 | 100 | Soil | China |
| SRR5992172 | SRX3148059 | PRJNA400517 | SRP116613 | 100 | Soil | China |
| SRR5992170 | SRX3148061 | PRJNA400517 | SRP116613 | 100 | Soil | China |
| SRR5992161 | SRX3148070 | PRJNA400517 | SRP116613 | 100 | Soil | China |
| SRR6025165 | SRX3175817 | PRJNA404015 | SRP117238 | 99.66 | Soil | China |
| SRR6025143 | SRX3175839 | PRJNA404015 | SRP117238 | 99.66 | Soil | China |
| SRR6027611 | SRX3177827 | PRJNA406830 | SRP117302 | 100 | Soil | China |
| SRR6027610 | SRX3177828 | PRJNA406830 | SRP117302 | 99.71 | Soil | China |
| SRR6027608 | SRX3177830 | PRJNA406830 | SRP117302 | 100 | Soil | China |
| SRR6027607 | SRX3177831 | PRJNA406830 | SRP117302 | 100 | Soil | China |
| SRR6027605 | SRX3177833 | PRJNA406830 | SRP117302 | 100 | Soil | China |
| SRR6027602 | SRX3177836 | PRJNA406830 | SRP117302 | 99.71 | Soil | China |
| SRR6027600 | SRX3177838 | PRJNA406830 | SRP117302 | 100 | Soil | China |
| SRR6027599 | SRX3177839 | PRJNA406830 | SRP117302 | 100 | Soil | China |
| SRR6027596 | SRX3177842 | PRJNA406830 | SRP117302 | 100 | Soil | China |
| SRR6027595 | SRX3177843 | PRJNA406830 | SRP117302 | 100 | Soil | China |
| SRR6027594 | SRX3177844 | PRJNA406830 | SRP117302 | 100 | Soil | China |
| SRR6027593 | SRX3177845 | PRJNA406830 | SRP117302 | 100 | Soil | China |
| SRR6027536 | SRX3177902 | PRJNA406830 | SRP117302 | 100 | Rhizosphere | China |
| SRR6027532 | SRX3177906 | PRJNA406830 | SRP117302 | 100 | Rhizosphere | China |
| SRR6027378 | SRX3178060 | PRJNA406830 | SRP117302 | 100 | Soil | China |
| SRR6027377 | SRX3178061 | PRJNA406830 | SRP117302 | 100 | Soil | China |
| SRR6027372 | SRX3178066 | PRJNA406830 | SRP117302 | 100 | Soil | China |
| SRR6027371 | SRX3178067 | PRJNA406830 | SRP117302 | 100 | Soil | China |
| SRR6027370 | SRX3178068 | PRJNA406830 | SRP117302 | 100 | Soil | China |
| SRR6027367 | SRX3178071 | PRJNA406830 | SRP117302 | 100 | Soil | China |
| SRR6027366 | SRX3178072 | PRJNA406830 | SRP117302 | 100 | Soil | China |
| SRR6027363 | SRX3178075 | PRJNA406830 | SRP117302 | 100 | Soil | China |
| SRR6058568 | SRX3204574 | PRJNA408324 | SRP118555 | 99.19 | Soil | China |
| SRR6058567 | SRX3204575 | PRJNA408324 | SRP118555 | 99.59 | Soil | China |
| SRR6058566 | SRX3204576 | PRJNA408324 | SRP118555 | 99.59 | Soil | China |
| SRR6058565 | SRX3204577 | PRJNA408324 | SRP118555 | 99.59 | Soil | China |
| SRR6058563 | SRX3204579 | PRJNA408324 | SRP118555 | 99.59 | Soil | China |
| SRR6058562 | SRX3204580 | PRJNA408324 | SRP118555 | 99.59 | Soil | China |
| SRR6058561 | SRX3204581 | PRJNA408324 | SRP118555 | 99.59 | Soil | China |
| SRR6058560 | SRX3204582 | PRJNA408324 | SRP118555 | 99.59 | Soil | China |
| SRR6058559 | SRX3204583 | PRJNA408324 | SRP118555 | 99.59 | Soil | China |
| SRR6058558 | SRX3204584 | PRJNA408324 | SRP118555 | 100 | Soil | China |
| SRR6058829 | SRX3204801 | PRJNA411758 | SRP118560 | 99.64 | Soil | China |
| SRR6058828 | SRX3204802 | PRJNA411758 | SRP118560 | 99.64 | Soil | China |
| SRR6058827 | SRX3204803 | PRJNA411758 | SRP118560 | 99.64 | Soil | China |
| SRR6058812 | SRX3204818 | PRJNA411758 | SRP118560 | 99.64 | Soil | China |
| SRR6058811 | SRX3204819 | PRJNA411758 | SRP118560 | 99.64 | Soil | China |
| SRR6074264 | SRX3214958 | PRJNA408090 | SRP118835 | 99.74 | Soil | China |
| SRR6074262 | SRX3214960 | PRJNA408090 | SRP118835 | 100 | Soil | China |
| SRR6074259 | SRX3214963 | PRJNA408090 | SRP118835 | 100 | Soil | China |
| SRR6074248 | SRX3214974 | PRJNA408090 | SRP118835 | 99.74 | Soil | China |
| SRR6074247 | SRX3214975 | PRJNA408090 | SRP118835 | 100 | Soil | China |
| SRR6074245 | SRX3214977 | PRJNA408090 | SRP118835 | 100 | Soil | China |
| SRR6074244 | SRX3214978 | PRJNA408090 | SRP118835 | 100 | Soil | China |
| SRR6074243 | SRX3214979 | PRJNA408090 | SRP118835 | 100 | Soil | China |
| SRR6074241 | SRX3214981 | PRJNA408090 | SRP118835 | 100 | Soil | China |
| SRR6074240 | SRX3214982 | PRJNA408090 | SRP118835 | 100 | Soil | China |
| SRR6074238 | SRX3214984 | PRJNA408090 | SRP118835 | 100 | Soil | China |
| SRR6074237 | SRX3214985 | PRJNA408090 | SRP118835 | 100 | Soil | China |
| SRR6172318 | SRX3283122 | PRJNA414050 | SRP119945 | 99.59 | Soil | China |
| SRR6172312 | SRX3283128 | PRJNA414050 | SRP119945 | 99.59 | Soil | China |
| SRR6172311 | SRX3283129 | PRJNA414050 | SRP119945 | 99.18 | Soil | China |
| SRR6227921 | SRX3336577 | PRJNA411794 | SRP122476 | 100 | Soil | China |
| SRR6227872 | SRX3336626 | PRJNA411794 | SRP122476 | 100 | Soil | China |
| SRR6227866 | SRX3336632 | PRJNA411794 | SRP122476 | 99.4 | Soil | China |
| SRR6302356 | SRX3403263 | PRJNA418913 | SRP125177 | 99.59 | Soil | China |
| SRR6302354 | SRX3403265 | PRJNA418913 | SRP125177 | 99.59 | Soil | China |
| SRR6339198 | SRX3438175 | PRJNA420428 | SRP126011 | 99.2 | Soil | China |
| SRR6377761 | SRX3472515 | PRJNA422480 | SRP126794 | 99.63 | Rhizosphere | China |
| SRR6377745 | SRX3472531 | PRJNA422480 | SRP126794 | 99.63 | Rhizosphere | China |
| SRR6377703 | SRX3472573 | PRJNA422480 | SRP126794 | 99.63 | Rhizosphere | China |
| SRR6424182 | SRX3516647 | PRJNA427693 | SRP127613 | 99.58 | Soil | China |
| SRR6431987 | SRX3523577 | PRJNA427939 | SRP127749 | 99.32 | Soil | China |
| SRR6431985 | SRX3523579 | PRJNA427939 | SRP127749 | 99.32 | Soil | China |
| SRR6431974 | SRX3523590 | PRJNA427939 | SRP127749 | 99.32 | Soil | China |
| SRR6439308 | SRX3530687 | PRJNA428429 | SRP128010 | 99.67 | Soil | China |
| SRR6439307 | SRX3530688 | PRJNA428429 | SRP128010 | 100 | Soil | China |
| SRR6439306 | SRX3530689 | PRJNA428429 | SRP128010 | 100 | Soil | China |
| SRR6439305 | SRX3530690 | PRJNA428429 | SRP128010 | 100 | Soil | China |
| SRR6474181 | SRX3564039 | PRJNA429689 | SRP129664 | 100 | Sewage sludge | China |
| SRR6474173 | SRX3564047 | PRJNA429689 | SRP129664 | 99.48 | Sewage sludge | China |
| SRR6474160 | SRX3564060 | PRJNA429689 | SRP129664 | 100 | Sewage sludge | China |
| SRR6474156 | SRX3564064 | PRJNA429689 | SRP129664 | 99.74 | Sewage sludge | China |
| SRR6474146 | SRX3564074 | PRJNA429689 | SRP129664 | 99.74 | Sewage sludge | China |
| SRR6474139 | SRX3564081 | PRJNA429689 | SRP129664 | 99.48 | Sewage sludge | China |
| SRR6516142 | SRX3604053 | PRJNA431692 | SRP131542 | 100 | Soil | China |
| SRR6516139 | SRX3604056 | PRJNA431692 | SRP131542 | 99.71 | Soil | China |
| SRR6516129 | SRX3604066 | PRJNA431692 | SRP131542 | 99.6 | Soil | China |
| SRR6516127 | SRX3604068 | PRJNA431692 | SRP131542 | 99.6 | Soil | China |
| SRR6516123 | SRX3604072 | PRJNA431692 | SRP131542 | 99.6 | Soil | China |
| SRR6516119 | SRX3604076 | PRJNA431692 | SRP131542 | 100 | Soil | China |
| SRR6516117 | SRX3604078 | PRJNA431692 | SRP131542 | 100 | Soil | China |
| SRR6516116 | SRX3604079 | PRJNA431692 | SRP131542 | 100 | Soil | China |
| SRR6516114 | SRX3604081 | PRJNA431692 | SRP131542 | 100 | Soil | China |
| SRR6516112 | SRX3604083 | PRJNA431692 | SRP131542 | 100 | Soil | China |
| SRR6516098 | SRX3604097 | PRJNA431692 | SRP131542 | 100 | Soil | China |
| SRR6516097 | SRX3604098 | PRJNA431692 | SRP131542 | 100 | Soil | China |
| SRR6516096 | SRX3604099 | PRJNA431692 | SRP131542 | 100 | Soil | China |
| SRR6516423 | SRX3604414 | PRJNA431842 | SRP131566 | 100 | Soil | China |
| SRR6516416 | SRX3604421 | PRJNA431842 | SRP131566 | 100 | Soil | China |
| SRR6701402 | SRX3675599 | PRJNA432446 | SRP132524 | 99.63 | Soil | China |
| SRR6701400 | SRX3675601 | PRJNA432446 | SRP132524 | 99.63 | Soil | China |
| SRR6701396 | SRX3675605 | PRJNA432446 | SRP132524 | 100 | Soil | China |
| SRR6701371 | SRX3675630 | PRJNA432446 | SRP132524 | 99.63 | Soil | China |
| SRR6702254 | SRX3676431 | PRJNA433313 | SRP132542 | 99.6 | Soil | China |
| SRR6834610 | SRX3790475 | PRJNA438048 | SRP135695 | 99.58 | Rhizosphere | China |
| SRR6874828 | SRX3827457 | PRJNA374472 | SRP099367 | 99.58 | Rhizosphere | China |
| SRR6892641 | SRX3842921 | PRJNA445521 | SRP136385 | 99.18 | Soil | China |
| SRR6921731 | SRX3869012 | PRJNA447943 | SRP136845 | 100 | Soil | China |
| SRR6929252 | SRX3875032 | PRJNA448501 | SRP136980 | 99.6 | Soil | China |
| SRR6960505 | SRX3903220 | PRJNA438855 | SRP138664 | 100 | Soil | China |
| SRR6960504 | SRX3903221 | PRJNA438855 | SRP138664 | 100 | Soil | China |
| SRR6960503 | SRX3903222 | PRJNA438855 | SRP138664 | 100 | Soil | China |
| SRR6960501 | SRX3903224 | PRJNA438855 | SRP138664 | 100 | Soil | China |
| SRR6960500 | SRX3903225 | PRJNA438855 | SRP138664 | 100 | Soil | China |
| SRR6960496 | SRX3903229 | PRJNA438855 | SRP138664 | 100 | Soil | China |
| SRR6960491 | SRX3903234 | PRJNA438855 | SRP138664 | 100 | Soil | China |
| SRR6960487 | SRX3903238 | PRJNA438855 | SRP138664 | 100 | Soil | China |
| SRR6960485 | SRX3903240 | PRJNA438855 | SRP138664 | 100 | Soil | China |
| SRR6960484 | SRX3903241 | PRJNA438855 | SRP138664 | 100 | Soil | China |
| SRR6963441 | SRX3905626 | PRJNA449192 | SRP138945 | 99.6 | Soil | China |
| SRR6963440 | SRX3905627 | PRJNA449192 | SRP138945 | 100 | Soil | China |
| SRR6963437 | SRX3905630 | PRJNA449192 | SRP138945 | 99.6 | Soil | China |
| SRR6963435 | SRX3905632 | PRJNA449192 | SRP138945 | 99.6 | Soil | China |
| SRR6963434 | SRX3905633 | PRJNA449192 | SRP138945 | 100 | Soil | China |
| SRR6963429 | SRX3905638 | PRJNA449192 | SRP138945 | 99.2 | Soil | China |
| SRR6963428 | SRX3905639 | PRJNA449192 | SRP138945 | 99.6 | Soil | China |
| SRR6963427 | SRX3905640 | PRJNA449192 | SRP138945 | 99.2 | Soil | China |
| SRR6963425 | SRX3905642 | PRJNA449192 | SRP138945 | 99.6 | Soil | China |
| SRR6963424 | SRX3905643 | PRJNA449192 | SRP138945 | 99.6 | Soil | China |
| SRR7014232 | SRX3946677 | PRJNA433474 | SRP132621 | 99.18 | Soil | China |
| SRR7058666 | SRX3989624 | PRJNA451472 | SRP142420 | 100 | Soil | China |
| SRR7058662 | SRX3989628 | PRJNA451472 | SRP142420 | 99.6 | Soil | China |
| SRR7086251 | SRX4015970 | PRJNA454180 | SRP144127 | 100 | Soil | China |
| SRR7086250 | SRX4015971 | PRJNA454180 | SRP144127 | 100 | Soil | China |
| SRR7086249 | SRX4015972 | PRJNA454180 | SRP144127 | 100 | Soil | China |
| SRR7086248 | SRX4015973 | PRJNA454180 | SRP144127 | 100 | Soil | China |
| SRR7086247 | SRX4015974 | PRJNA454180 | SRP144127 | 100 | Soil | China |
| SRR7086246 | SRX4015975 | PRJNA454180 | SRP144127 | 100 | Soil | China |
| SRR7086245 | SRX4015976 | PRJNA454180 | SRP144127 | 99.48 | Soil | China |
| SRR7086243 | SRX4015978 | PRJNA454180 | SRP144127 | 100 | Soil | China |
| SRR7086242 | SRX4015979 | PRJNA454180 | SRP144127 | 100 | Soil | China |
| SRR7086241 | SRX4015980 | PRJNA454180 | SRP144127 | 100 | Soil | China |
| SRR7086240 | SRX4015981 | PRJNA454180 | SRP144127 | 100 | Soil | China |
| SRR7086236 | SRX4015985 | PRJNA454180 | SRP144127 | 100 | Soil | China |
| SRR7086235 | SRX4015986 | PRJNA454180 | SRP144127 | 100 | Soil | China |
| SRR7086232 | SRX4015989 | PRJNA454180 | SRP144127 | 100 | Soil | China |
| SRR7086231 | SRX4015990 | PRJNA454180 | SRP144127 | 100 | Soil | China |
| SRR7086230 | SRX4015991 | PRJNA454180 | SRP144127 | 100 | Soil | China |
| SRR7086228 | SRX4015993 | PRJNA454180 | SRP144127 | 100 | Soil | China |
| SRR7223774 | SRX4130286 | PRJNA473265 | SRP149050 | 100 | Soil | China |
| SRR7223770 | SRX4130290 | PRJNA473265 | SRP149050 | 99.71 | Soil | China |
| SRR7276024 | SRX4179604 | PRJNA473900 | SRP149967 | 99.58 | Soil | China |
| SRR7287258 | SRX4190792 | PRJNA475449 | SRP150167 | 99.7 | Compost | China |
| SRR7287257 | SRX4190793 | PRJNA475449 | SRP150167 | 99.7 | Compost | China |
| SRR7287251 | SRX4190799 | PRJNA475449 | SRP150167 | 99.7 | Compost | China |
| SRR7287249 | SRX4190801 | PRJNA475449 | SRP150167 | 99.7 | Compost | China |
| SRR7369788 | SRX4242709 | PRJNA476807 | SRP150889 | 99.6 | Soil | China |
| SRR7369748 | SRX4242749 | PRJNA476807 | SRP150889 | 99.6 | Soil | China |
| SRR7420742 | SRX4291315 | PRJNA475307 | SRP151262 | 99.58 | Soil | China |
| SRR7420741 | SRX4291316 | PRJNA475307 | SRP151262 | 99.58 | Soil | China |
| SRR7420738 | SRX4291319 | PRJNA475307 | SRP151262 | 99.58 | Soil | China |
| SRR7420736 | SRX4291321 | PRJNA475307 | SRP151262 | 99.62 | Soil | China |
| SRR7420623 | SRX4291434 | PRJNA475307 | SRP151262 | 99.58 | Soil | China |
| SRR7420618 | SRX4291439 | PRJNA475307 | SRP151262 | 99.16 | Soil | China |
| SRR7420545 | SRX4291512 | PRJNA475307 | SRP151262 | 100 | Soil | China |
| SRR7420543 | SRX4291514 | PRJNA475307 | SRP151262 | 99.58 | Soil | China |
| SRR7420542 | SRX4291515 | PRJNA475307 | SRP151262 | 99.58 | Soil | China |
| SRR7420539 | SRX4291518 | PRJNA475307 | SRP151262 | 99.16 | Soil | China |
| SRR7420537 | SRX4291520 | PRJNA475307 | SRP151262 | 99.16 | Soil | China |
| SRR7420534 | SRX4291523 | PRJNA475307 | SRP151262 | 99.62 | Soil | China |
| SRR7420494 | SRX4291563 | PRJNA475307 | SRP151262 | 99.58 | Soil | China |
| SRR7420491 | SRX4291566 | PRJNA475307 | SRP151262 | 99.58 | Soil | China |
| SRR7421073 | SRX4291656 | PRJNA475307 | SRP151262 | 99.58 | Soil | China |
| SRR7421072 | SRX4291657 | PRJNA475307 | SRP151262 | 99.58 | Soil | China |
| SRR7420998 | SRX4291731 | PRJNA475307 | SRP151262 | 99.23 | Soil | China |
| SRR7420961 | SRX4291768 | PRJNA475307 | SRP151262 | 99.58 | Soil | China |
| SRR7420948 | SRX4291781 | PRJNA475307 | SRP151262 | 100 | Soil | China |
| SRR7420920 | SRX4291809 | PRJNA475307 | SRP151262 | 99.62 | Soil | China |
| SRR7420862 | SRX4291867 | PRJNA475307 | SRP151262 | 99.16 | Soil | China |
| SRR7420856 | SRX4291873 | PRJNA475307 | SRP151262 | 100 | Soil | China |
| SRR7420819 | SRX4291910 | PRJNA475307 | SRP151262 | 99.58 | Soil | China |
| SRR7420814 | SRX4291915 | PRJNA475307 | SRP151262 | 100 | Soil | China |
| SRR7420813 | SRX4291916 | PRJNA475307 | SRP151262 | 99.58 | Soil | China |
| SRR7420796 | SRX4291933 | PRJNA475307 | SRP151262 | 99.16 | Soil | China |
| SRR7420794 | SRX4291935 | PRJNA475307 | SRP151262 | 99.58 | Soil | China |
| SRR7420793 | SRX4291936 | PRJNA475307 | SRP151262 | 99.16 | Soil | China |
| SRR7420791 | SRX4291938 | PRJNA475307 | SRP151262 | 99.58 | Soil | China |
| SRR7420790 | SRX4291939 | PRJNA475307 | SRP151262 | 99.16 | Soil | China |
| SRR7420789 | SRX4291940 | PRJNA475307 | SRP151262 | 99.58 | Soil | China |
| SRR7420788 | SRX4291941 | PRJNA475307 | SRP151262 | 99.61 | Soil | China |
| SRR7420786 | SRX4291943 | PRJNA475307 | SRP151262 | 99.58 | Soil | China |
| SRR7421303 | SRX4291990 | PRJNA475307 | SRP151262 | 99.58 | Soil | China |
| SRR7421301 | SRX4291992 | PRJNA475307 | SRP151262 | 99.58 | Soil | China |
| SRR7421136 | SRX4292157 | PRJNA475307 | SRP151262 | 99.58 | Soil | China |
| SRR7444682 | SRX4315208 | PRJNA478264 | SRP151508 | 99.59 | Rhizosphere | China |
| SRR7444662 | SRX4315228 | PRJNA478264 | SRP151508 | 99.59 | Rhizosphere | China |
| SRR7469810 | SRX4339561 | PRJNA479548 | SRP151963 | 99.6 | Soil | China |
| SRR7469809 | SRX4339562 | PRJNA479548 | SRP151963 | 99.6 | Soil | China |
| SRR7469807 | SRX4339564 | PRJNA479548 | SRP151963 | 100 | Soil | China |
| SRR7649837 | SRX4512745 | PRJNA484686 | SRP156485 | 100 | Soil | China |
| SRR7649835 | SRX4512747 | PRJNA484686 | SRP156485 | 99.6 | Soil | China |
| SRR7649830 | SRX4512752 | PRJNA484686 | SRP156485 | 99.2 | Soil | China |
| SRR7649829 | SRX4512753 | PRJNA484686 | SRP156485 | 99.2 | Rhizosphere | China |
| SRR7663530 | SRX4524108 | PRJNA485233 | SRP156834 | 99.59 | Soil | China |
| SRR7702862 | SRX4560200 | PRJNA486218 | SRP158134 | 99.18 | Soil | China |
| SRR7702777 | SRX4560285 | PRJNA486218 | SRP158134 | 99.18 | Soil | China |
| SRR7702776 | SRX4560286 | PRJNA486218 | SRP158134 | 99.59 | Soil | China |
| SRR7750155 | SRX4606031 | PRJNA487497 | SRP158731 | 99.58 | Soil | China |
| SRR7750262 | SRX4606165 | PRJNA487669 | SRP158738 | 99.32 | Rhizosphere | China |
| SRR7750247 | SRX4606180 | PRJNA487669 | SRP158738 | 99.32 | Rhizosphere | China |
| SRR7755051 | SRX4610963 | PRJNA477677 | SRP151204 | 99.4 | Soil | China |
| SRR7800956 | SRX4652772 | PRJNA488683 | SRP159420 | 99.19 | Soil | China |
| SRR7800955 | SRX4652773 | PRJNA488683 | SRP159420 | 99.19 | Soil | China |
| SRR7800950 | SRX4652778 | PRJNA488683 | SRP159420 | 99.19 | Soil | China |
| SRR7800949 | SRX4652779 | PRJNA488683 | SRP159420 | 99.19 | Soil | China |
| SRR7800947 | SRX4652781 | PRJNA488683 | SRP159420 | 99.19 | Soil | China |
| SRR7800941 | SRX4652787 | PRJNA488683 | SRP159420 | 99.6 | Soil | China |
| SRR7800940 | SRX4652788 | PRJNA488683 | SRP159420 | 99.19 | Soil | China |
| SRR7800939 | SRX4652789 | PRJNA488683 | SRP159420 | 99.6 | Soil | China |
| SRR7800938 | SRX4652790 | PRJNA488683 | SRP159420 | 99.6 | Soil | China |
| SRR7817787 | SRX4669165 | PRJNA490282 | SRP161464 | 99.31 | Soil | China |
| SRR7817754 | SRX4669198 | PRJNA490282 | SRP161464 | 99.32 | Soil | China |
| SRR7817753 | SRX4669199 | PRJNA490282 | SRP161464 | 99.66 | Soil | China |
| SRR7817745 | SRX4669207 | PRJNA490282 | SRP161464 | 99.32 | Soil | China |
| SRR7817738 | SRX4669214 | PRJNA490282 | SRP161464 | 99.66 | Soil | China |
| SRR7817735 | SRX4669217 | PRJNA490282 | SRP161464 | 99.66 | Soil | China |
| SRR7817733 | SRX4669219 | PRJNA490282 | SRP161464 | 99.66 | Soil | China |
| SRR7829922 | SRX4680878 | PRJNA490841 | SRP161774 | 99.59 | Soil | China |
| SRR7829918 | SRX4680882 | PRJNA490841 | SRP161774 | 99.59 | Soil | China |
| SRR7829911 | SRX4680889 | PRJNA490841 | SRP161774 | 99.59 | Soil | China |
| SRR7944790 | SRX4778957 | PRJNA493986 | SRP162954 | 99.6 | Soil | China |
| SRR7975483 | SRX4808305 | PRJNA494942 | SRP164287 | 99.6 | Soil | China |
| SRR7975480 | SRX4808308 | PRJNA494942 | SRP164287 | 99.6 | Soil | China |
| SRR7975479 | SRX4808309 | PRJNA494942 | SRP164287 | 99.6 | Soil | China |
| SRR8039752 | SRX4870371 | PRJNA495753 | SRP165261 | 100 | Soil | China |
| SRR8039794 | SRX4870381 | PRJNA495753 | SRP165261 | 99.6 | Soil | China |
| SRR8039793 | SRX4870382 | PRJNA495753 | SRP165261 | 99.6 | Soil | China |
| SRR8039792 | SRX4870383 | PRJNA495753 | SRP165261 | 99.6 | Soil | China |
| SRR8039791 | SRX4870384 | PRJNA495753 | SRP165261 | 99.6 | Soil | China |
| SRR8039790 | SRX4870385 | PRJNA495753 | SRP165261 | 99.6 | Soil | China |
| SRR8039789 | SRX4870386 | PRJNA495753 | SRP165261 | 99.6 | Soil | China |
| SRR8039788 | SRX4870387 | PRJNA495753 | SRP165261 | 99.6 | Soil | China |
| SRR8039787 | SRX4870388 | PRJNA495753 | SRP165261 | 99.6 | Soil | China |
| SRR8039786 | SRX4870389 | PRJNA495753 | SRP165261 | 99.6 | Soil | China |
| SRR8039785 | SRX4870390 | PRJNA495753 | SRP165261 | 99.6 | Soil | China |
| SRR8039784 | SRX4870391 | PRJNA495753 | SRP165261 | 99.6 | Soil | China |
| SRR8039783 | SRX4870392 | PRJNA495753 | SRP165261 | 99.6 | Soil | China |
| SRR8039782 | SRX4870393 | PRJNA495753 | SRP165261 | 99.6 | Soil | China |
| SRR8039781 | SRX4870394 | PRJNA495753 | SRP165261 | 99.6 | Soil | China |
| SRR8039780 | SRX4870395 | PRJNA495753 | SRP165261 | 99.6 | Soil | China |
| SRR8039779 | SRX4870396 | PRJNA495753 | SRP165261 | 99.6 | Soil | China |
| SRR8039778 | SRX4870397 | PRJNA495753 | SRP165261 | 99.6 | Soil | China |
| SRR8039777 | SRX4870398 | PRJNA495753 | SRP165261 | 99.6 | Soil | China |
| SRR8039776 | SRX4870399 | PRJNA495753 | SRP165261 | 100 | Soil | China |
| SRR8039775 | SRX4870400 | PRJNA495753 | SRP165261 | 99.6 | Soil | China |
| SRR8040066 | SRX4870638 | PRJNA495669 | SRP165269 | 99.59 | Soil | China |
| SRR8040048 | SRX4870656 | PRJNA495669 | SRP165269 | 99.59 | Soil | China |
| SRR8040014 | SRX4870690 | PRJNA495669 | SRP165269 | 99.59 | Soil | China |
| SRR8040007 | SRX4870697 | PRJNA495669 | SRP165269 | 99.59 | Soil | China |
| SRR8040006 | SRX4870698 | PRJNA495669 | SRP165269 | 100 | Soil | China |
| SRR8056349 | SRX4885946 | PRJNA362455 | SRP097606 | 100 | Rhizosphere | China |
| SRR8056348 | SRX4885947 | PRJNA362455 | SRP097606 | 100 | Rhizosphere | China |
| SRR8128627 | SRX4949833 | PRJNA498757 | SRP167123 | 99.58 | Rhizosphere | China |
| SRR8128626 | SRX4949834 | PRJNA498757 | SRP167123 | 99.58 | Rhizosphere | China |
| SRR8128625 | SRX4949835 | PRJNA498757 | SRP167123 | 99.58 | Rhizosphere | China |
| SRR8133918 | SRX4954925 | PRJNA501803 | SRP167219 | 99.63 | Soil | China |
| SRR8133905 | SRX4954938 | PRJNA501803 | SRP167219 | 99.63 | Soil | China |
| SRR8133904 | SRX4954939 | PRJNA501803 | SRP167219 | 99.63 | Soil | China |
| SRR8133903 | SRX4954940 | PRJNA501803 | SRP167219 | 99.63 | Soil | China |
| SRR8133901 | SRX4954942 | PRJNA501803 | SRP167219 | 99.63 | Soil | China |
| SRR8186569 | SRX5006370 | PRJNA505085 | SRP168578 | 99.59 | Soil | China |
| SRR8186555 | SRX5006384 | PRJNA505085 | SRP168578 | 99.59 | Soil | China |
| SRR8224209 | SRX5042679 | PRJNA506286 | SRP169989 | 99.59 | Soil | China |
| SRR8224191 | SRX5042697 | PRJNA506286 | SRP169989 | 99.59 | Soil | China |
| SRR8247279 | SRX5065336 | PRJNA507126 | SRP170924 | 100 | Soil | China |
| SRR8247263 | SRX5065352 | PRJNA507126 | SRP170924 | 100 | Soil | China |
| SRR8281902 | SRX5096524 | PRJNA508409 | SRP172511 | 100 | Soil | China |
| SRR8281900 | SRX5096526 | PRJNA508409 | SRP172511 | 100 | Soil | China |
| SRR8281899 | SRX5096527 | PRJNA508409 | SRP172511 | 100 | Soil | China |
| SRR8281898 | SRX5096528 | PRJNA508409 | SRP172511 | 99.71 | Soil | China |
| SRR8281896 | SRX5096530 | PRJNA508409 | SRP172511 | 100 | Soil | China |
| SRR8281878 | SRX5096548 | PRJNA508409 | SRP172511 | 100 | Soil | China |
| SRR8281861 | SRX5096565 | PRJNA508409 | SRP172511 | 100 | Soil | China |
| SRR8281860 | SRX5096566 | PRJNA508409 | SRP172511 | 100 | Soil | China |
| SRR8281813 | SRX5096613 | PRJNA508409 | SRP172511 | 100 | Soil | China |
| SRR8281808 | SRX5096618 | PRJNA508409 | SRP172511 | 100 | Soil | China |
| SRR8281807 | SRX5096619 | PRJNA508409 | SRP172511 | 100 | Soil | China |
| SRR8281806 | SRX5096620 | PRJNA508409 | SRP172511 | 100 | Soil | China |
| SRR8281795 | SRX5096631 | PRJNA508409 | SRP172511 | 99.71 | Soil | China |
| SRR8281793 | SRX5096633 | PRJNA508409 | SRP172511 | 99.29 | Soil | China |
| SRR8281785 | SRX5096641 | PRJNA508409 | SRP172511 | 100 | Soil | China |
| SRR8281784 | SRX5096642 | PRJNA508409 | SRP172511 | 100 | Soil | China |
| SRR8281778 | SRX5096648 | PRJNA508409 | SRP172511 | 100 | Soil | China |
| SRR8281776 | SRX5096650 | PRJNA508409 | SRP172511 | 100 | Soil | China |
| SRR8281763 | SRX5096663 | PRJNA508409 | SRP172511 | 100 | Soil | China |
| SRR8281758 | SRX5096668 | PRJNA508409 | SRP172511 | 100 | Soil | China |
| SRR8281745 | SRX5096681 | PRJNA508409 | SRP172511 | 100 | Soil | China |
| SRR8281744 | SRX5096682 | PRJNA508409 | SRP172511 | 100 | Soil | China |
| SRR8281742 | SRX5096684 | PRJNA508409 | SRP172511 | 100 | Soil | China |
| SRR8281739 | SRX5096687 | PRJNA508409 | SRP172511 | 100 | Soil | China |
| SRR8281738 | SRX5096688 | PRJNA508409 | SRP172511 | 100 | Soil | China |
| SRR8281737 | SRX5096689 | PRJNA508409 | SRP172511 | 99.71 | Soil | China |
| SRR8281736 | SRX5096690 | PRJNA508409 | SRP172511 | 100 | Soil | China |
| SRR8375729 | SRX5185683 | PRJNA512054 | SRP174626 | 99.65 | Rhizosphere | China |
| SRR8400283 | SRX5209449 | PRJNA513140 | SRP175610 | 100 | Soil | China |
| SRR8400259 | SRX5209473 | PRJNA513140 | SRP175610 | 100 | Soil | China |
| SRR8400258 | SRX5209474 | PRJNA513140 | SRP175610 | 100 | Soil | China |
| SRR8400257 | SRX5209475 | PRJNA513140 | SRP175610 | 100 | Soil | China |
| SRR8400256 | SRX5209476 | PRJNA513140 | SRP175610 | 100 | Soil | China |
| SRR8400255 | SRX5209477 | PRJNA513140 | SRP175610 | 100 | Soil | China |
| SRR8400254 | SRX5209478 | PRJNA513140 | SRP175610 | 100 | Soil | China |
| SRR8400253 | SRX5209479 | PRJNA513140 | SRP175610 | 100 | Soil | China |
| SRR8400252 | SRX5209480 | PRJNA513140 | SRP175610 | 100 | Soil | China |
| SRR8400251 | SRX5209481 | PRJNA513140 | SRP175610 | 100 | Soil | China |
| SRR8400250 | SRX5209482 | PRJNA513140 | SRP175610 | 100 | Soil | China |
| SRR8400249 | SRX5209483 | PRJNA513140 | SRP175610 | 100 | Soil | China |
| SRR8400248 | SRX5209484 | PRJNA513140 | SRP175610 | 100 | Soil | China |
| SRR8400247 | SRX5209485 | PRJNA513140 | SRP175610 | 100 | Soil | China |
| SRR8400246 | SRX5209486 | PRJNA513140 | SRP175610 | 99.71 | Soil | China |
| SRR8400244 | SRX5209488 | PRJNA513140 | SRP175610 | 100 | Soil | China |
| SRR8400243 | SRX5209489 | PRJNA513140 | SRP175610 | 100 | Soil | China |
| SRR8400239 | SRX5209493 | PRJNA513140 | SRP175610 | 99.71 | Soil | China |
| SRR8400205 | SRX5209527 | PRJNA513140 | SRP175610 | 100 | Soil | China |
| SRR8400204 | SRX5209528 | PRJNA513140 | SRP175610 | 100 | Soil | China |
| SRR8400203 | SRX5209529 | PRJNA513140 | SRP175610 | 100 | Soil | China |
| SRR8400202 | SRX5209530 | PRJNA513140 | SRP175610 | 100 | Soil | China |
| SRR8400201 | SRX5209531 | PRJNA513140 | SRP175610 | 100 | Soil | China |
| SRR8400200 | SRX5209532 | PRJNA513140 | SRP175610 | 100 | Soil | China |
| SRR8400197 | SRX5209535 | PRJNA513140 | SRP175610 | 100 | Soil | China |
| SRR8400181 | SRX5209551 | PRJNA513140 | SRP175610 | 100 | Soil | China |
| SRR8400180 | SRX5209552 | PRJNA513140 | SRP175610 | 100 | Soil | China |
| SRR8400178 | SRX5209554 | PRJNA513140 | SRP175610 | 100 | Soil | China |
| SRR8400176 | SRX5209556 | PRJNA513140 | SRP175610 | 100 | Soil | China |
| SRR8400164 | SRX5209568 | PRJNA513140 | SRP175610 | 100 | Soil | China |
| SRR8400162 | SRX5209570 | PRJNA513140 | SRP175610 | 100 | Soil | China |
| SRR8400161 | SRX5209571 | PRJNA513140 | SRP175610 | 100 | Soil | China |
| SRR8400159 | SRX5209573 | PRJNA513140 | SRP175610 | 100 | Soil | China |
| SRR8400142 | SRX5209590 | PRJNA513140 | SRP175610 | 100 | Soil | China |
| SRR8400138 | SRX5209594 | PRJNA513140 | SRP175610 | 100 | Soil | China |
| SRR8400137 | SRX5209595 | PRJNA513140 | SRP175610 | 100 | Soil | China |
| SRR8400135 | SRX5209597 | PRJNA513140 | SRP175610 | 100 | Soil | China |
| SRR8465851 | SRX5272370 | PRJNA516026 | SRP180582 | 99.63 | Soil | China |
| SRR8465840 | SRX5272381 | PRJNA516026 | SRP180582 | 100 | Soil | China |
| SRR8465837 | SRX5272384 | PRJNA516026 | SRP180582 | 99.6 | Soil | China |
| SRR8465836 | SRX5272385 | PRJNA516026 | SRP180582 | 99.6 | Soil | China |
| SRR8465835 | SRX5272386 | PRJNA516026 | SRP180582 | 100 | Soil | China |
| SRR8465833 | SRX5272388 | PRJNA516026 | SRP180582 | 99.63 | Soil | China |
| SRR8465797 | SRX5272424 | PRJNA516026 | SRP180582 | 99.63 | Soil | China |
| SRR8465791 | SRX5272430 | PRJNA516026 | SRP180582 | 100 | Soil | China |
| SRR8490221 | SRX5294778 | PRJNA517215 | SRP182118 | 99.64 | Soil | China |
| SRR8490211 | SRX5294788 | PRJNA517215 | SRP182118 | 99.64 | Soil | China |
| SRR8490546 | SRX5295125 | PRJNA517215 | SRP182118 | 99.64 | Soil | China |
| SRR8490544 | SRX5295127 | PRJNA517215 | SRP182118 | 99.64 | Soil | China |
| SRR8584839 | SRX5385360 | PRJNA522870 | SRP186099 | 99.59 | Soil | China |
| SRR8584838 | SRX5385361 | PRJNA522870 | SRP186099 | 100 | Soil | China |
| SRR8584836 | SRX5385363 | PRJNA522870 | SRP186099 | 99.59 | Soil | China |
| SRR8584829 | SRX5385370 | PRJNA522870 | SRP186099 | 99.59 | Soil | China |
| SRR8584826 | SRX5385373 | PRJNA522870 | SRP186099 | 99.59 | Soil | China |
| SRR8584825 | SRX5385374 | PRJNA522870 | SRP186099 | 99.18 | Soil | China |
| SRR8584814 | SRX5385385 | PRJNA522870 | SRP186099 | 99.59 | Soil | China |
| SRR8585867 | SRX5386217 | PRJNA522337 | SRP186114 | 99.21 | Soil | China |
| SRR8585715 | SRX5386368 | PRJNA522337 | SRP186114 | 99.21 | Soil | China |
| SRR8585698 | SRX5386385 | PRJNA522337 | SRP186114 | 99.6 | Soil | China |
| SRR8586995 | SRX5387523 | PRJNA522966 | SRP186150 | 100 | Rhizosphere | China |
| SRR8589366 | SRX5389657 | PRJNA523105 | SRP186221 | 100 | Soil | China |
| SRR8589364 | SRX5389659 | PRJNA523105 | SRP186221 | 99.63 | Soil | China |
| SRR8589363 | SRX5389660 | PRJNA523105 | SRP186221 | 99.26 | Soil | China |
| SRR8589362 | SRX5389661 | PRJNA523105 | SRP186221 | 99.63 | Soil | China |
| SRR8589361 | SRX5389662 | PRJNA523105 | SRP186221 | 99.63 | Soil | China |
| SRR8589352 | SRX5389671 | PRJNA523105 | SRP186221 | 100 | Soil | China |
| SRR8589351 | SRX5389672 | PRJNA523105 | SRP186221 | 100 | Soil | China |
| SRR8589349 | SRX5389674 | PRJNA523105 | SRP186221 | 100 | Soil | China |
| SRR8589346 | SRX5389677 | PRJNA523105 | SRP186221 | 100 | Soil | China |
| SRR8589345 | SRX5389678 | PRJNA523105 | SRP186221 | 100 | Soil | China |
| SRR8590723 | SRX5390956 | PRJNA523168 | SRP186249 | 100 | Soil | China |
| SRR8590716 | SRX5390963 | PRJNA523168 | SRP186249 | 100 | Soil | China |
| SRR8590715 | SRX5390964 | PRJNA523168 | SRP186249 | 100 | Soil | China |
| SRR8590714 | SRX5390965 | PRJNA523168 | SRP186249 | 100 | Soil | China |
| SRR8590713 | SRX5390966 | PRJNA523168 | SRP186249 | 100 | Soil | China |
| SRR8590710 | SRX5390969 | PRJNA523168 | SRP186249 | 100 | Soil | China |
| SRR8590696 | SRX5390983 | PRJNA523168 | SRP186249 | 100 | Soil | China |
| SRR8590693 | SRX5390986 | PRJNA523168 | SRP186249 | 100 | Soil | China |
| SRR8590690 | SRX5390989 | PRJNA523168 | SRP186249 | 100 | Soil | China |
| SRR8590687 | SRX5390992 | PRJNA523168 | SRP186249 | 100 | Soil | China |
| SRR8590686 | SRX5390993 | PRJNA523168 | SRP186249 | 100 | Soil | China |
| SRR8590685 | SRX5390994 | PRJNA523168 | SRP186249 | 100 | Soil | China |
| SRR8590679 | SRX5391000 | PRJNA523168 | SRP186249 | 100 | Soil | China |
| SRR8590678 | SRX5391001 | PRJNA523168 | SRP186249 | 100 | Soil | China |
| SRR8590675 | SRX5391004 | PRJNA523168 | SRP186249 | 100 | Soil | China |
| SRR8590661 | SRX5391018 | PRJNA523168 | SRP186249 | 100 | Soil | China |
| SRR8607161 | SRX5406340 | PRJNA523309 | SRP186537 | 99.58 | Soil | China |
| SRR8607160 | SRX5406341 | PRJNA523309 | SRP186537 | 100 | Soil | China |
| SRR8607159 | SRX5406342 | PRJNA523309 | SRP186537 | 100 | Soil | China |
| SRR8607158 | SRX5406343 | PRJNA523309 | SRP186537 | 100 | Soil | China |
| SRR8607157 | SRX5406344 | PRJNA523309 | SRP186537 | 100 | Soil | China |
| SRR8607156 | SRX5406345 | PRJNA523309 | SRP186537 | 100 | Soil | China |
| SRR8607155 | SRX5406346 | PRJNA523309 | SRP186537 | 100 | Soil | China |
| SRR8607154 | SRX5406347 | PRJNA523309 | SRP186537 | 100 | Soil | China |
| SRR8607153 | SRX5406348 | PRJNA523309 | SRP186537 | 100 | Soil | China |
| SRR8607152 | SRX5406349 | PRJNA523309 | SRP186537 | 100 | Soil | China |
| SRR8607151 | SRX5406350 | PRJNA523309 | SRP186537 | 100 | Soil | China |
| SRR8607150 | SRX5406351 | PRJNA523309 | SRP186537 | 100 | Soil | China |
| SRR8607129 | SRX5406372 | PRJNA523309 | SRP186537 | 100 | Soil | China |
| SRR8607128 | SRX5406373 | PRJNA523309 | SRP186537 | 100 | Soil | China |
| SRR8607127 | SRX5406374 | PRJNA523309 | SRP186537 | 100 | Soil | China |
| SRR8607126 | SRX5406375 | PRJNA523309 | SRP186537 | 100 | Soil | China |
| SRR8607125 | SRX5406376 | PRJNA523309 | SRP186537 | 100 | Soil | China |
| SRR8607124 | SRX5406377 | PRJNA523309 | SRP186537 | 100 | Soil | China |
| SRR8607123 | SRX5406378 | PRJNA523309 | SRP186537 | 100 | Soil | China |
| SRR8607121 | SRX5406380 | PRJNA523309 | SRP186537 | 100 | Soil | China |
| SRR8607120 | SRX5406381 | PRJNA523309 | SRP186537 | 100 | Soil | China |
| SRR8607119 | SRX5406382 | PRJNA523309 | SRP186537 | 100 | Soil | China |
| SRR8607118 | SRX5406383 | PRJNA523309 | SRP186537 | 100 | Soil | China |
| SRR8607117 | SRX5406384 | PRJNA523309 | SRP186537 | 99.58 | Soil | China |
| SRR8607116 | SRX5406385 | PRJNA523309 | SRP186537 | 100 | Soil | China |
| SRR8607115 | SRX5406386 | PRJNA523309 | SRP186537 | 99.58 | Soil | China |
| SRR8607090 | SRX5406411 | PRJNA523309 | SRP186537 | 100 | Soil | China |
| SRR8607089 | SRX5406412 | PRJNA523309 | SRP186537 | 100 | Soil | China |
| SRR8607088 | SRX5406413 | PRJNA523309 | SRP186537 | 100 | Soil | China |
| SRR8607087 | SRX5406414 | PRJNA523309 | SRP186537 | 100 | Soil | China |
| SRR8607086 | SRX5406415 | PRJNA523309 | SRP186537 | 99.58 | Soil | China |
| SRR8607085 | SRX5406416 | PRJNA523309 | SRP186537 | 99.58 | Soil | China |
| SRR8607084 | SRX5406417 | PRJNA523309 | SRP186537 | 99.58 | Soil | China |
| SRR8607082 | SRX5406419 | PRJNA523309 | SRP186537 | 100 | Soil | China |
| SRR8607078 | SRX5406423 | PRJNA523309 | SRP186537 | 100 | Soil | China |
| SRR8607065 | SRX5406436 | PRJNA523309 | SRP186537 | 99.58 | Soil | China |
| SRR8607064 | SRX5406437 | PRJNA523309 | SRP186537 | 100 | Soil | China |
| SRR8607061 | SRX5406440 | PRJNA523309 | SRP186537 | 100 | Soil | China |
| SRR8607060 | SRX5406441 | PRJNA523309 | SRP186537 | 100 | Soil | China |
| SRR8607059 | SRX5406442 | PRJNA523309 | SRP186537 | 100 | Soil | China |
| SRR8607057 | SRX5406444 | PRJNA523309 | SRP186537 | 100 | Soil | China |
| SRR8607056 | SRX5406445 | PRJNA523309 | SRP186537 | 100 | Soil | China |
| SRR8607055 | SRX5406446 | PRJNA523309 | SRP186537 | 100 | Soil | China |
| SRR8607043 | SRX5406458 | PRJNA523309 | SRP186537 | 100 | Soil | China |
| SRR8607040 | SRX5406461 | PRJNA523309 | SRP186537 | 100 | Soil | China |
| SRR8607039 | SRX5406462 | PRJNA523309 | SRP186537 | 100 | Soil | China |
| SRR8607038 | SRX5406463 | PRJNA523309 | SRP186537 | 100 | Soil | China |
| SRR8607037 | SRX5406464 | PRJNA523309 | SRP186537 | 99.58 | Soil | China |
| SRR8607033 | SRX5406468 | PRJNA523309 | SRP186537 | 100 | Soil | China |
| SRR8607031 | SRX5406470 | PRJNA523309 | SRP186537 | 100 | Soil | China |
| SRR8607030 | SRX5406471 | PRJNA523309 | SRP186537 | 100 | Soil | China |
| SRR8607028 | SRX5406473 | PRJNA523309 | SRP186537 | 100 | Soil | China |
| SRR8607027 | SRX5406474 | PRJNA523309 | SRP186537 | 100 | Soil | China |
| SRR8607026 | SRX5406475 | PRJNA523309 | SRP186537 | 100 | Soil | China |
| SRR8607025 | SRX5406476 | PRJNA523309 | SRP186537 | 100 | Soil | China |
| SRR8607024 | SRX5406477 | PRJNA523309 | SRP186537 | 99.58 | Soil | China |
| SRR8607023 | SRX5406478 | PRJNA523309 | SRP186537 | 100 | Soil | China |
| SRR8607022 | SRX5406479 | PRJNA523309 | SRP186537 | 100 | Soil | China |
| SRR8607021 | SRX5406480 | PRJNA523309 | SRP186537 | 100 | Soil | China |
| SRR8607020 | SRX5406481 | PRJNA523309 | SRP186537 | 100 | Soil | China |
| SRR8607019 | SRX5406482 | PRJNA523309 | SRP186537 | 100 | Soil | China |
| SRR8607007 | SRX5406494 | PRJNA523309 | SRP186537 | 100 | Soil | China |
| SRR8607004 | SRX5406497 | PRJNA523309 | SRP186537 | 100 | Soil | China |
| SRR8607002 | SRX5406499 | PRJNA523309 | SRP186537 | 100 | Soil | China |
| SRR8607001 | SRX5406500 | PRJNA523309 | SRP186537 | 100 | Soil | China |
| SRR8606999 | SRX5406502 | PRJNA523309 | SRP186537 | 100 | Soil | China |
| SRR8606998 | SRX5406503 | PRJNA523309 | SRP186537 | 100 | Soil | China |
| SRR8606993 | SRX5406508 | PRJNA523309 | SRP186537 | 100 | Soil | China |
| SRR8606992 | SRX5406509 | PRJNA523309 | SRP186537 | 100 | Soil | China |
| SRR8606989 | SRX5406512 | PRJNA523309 | SRP186537 | 100 | Soil | China |
| SRR8606988 | SRX5406513 | PRJNA523309 | SRP186537 | 100 | Soil | China |
| SRR8606985 | SRX5406516 | PRJNA523309 | SRP186537 | 100 | Soil | China |
| SRR8606984 | SRX5406517 | PRJNA523309 | SRP186537 | 100 | Soil | China |
| SRR8606982 | SRX5406519 | PRJNA523309 | SRP186537 | 100 | Soil | China |
| SRR8606977 | SRX5406524 | PRJNA523309 | SRP186537 | 100 | Soil | China |
| SRR8606976 | SRX5406525 | PRJNA523309 | SRP186537 | 100 | Soil | China |
| SRR8606975 | SRX5406526 | PRJNA523309 | SRP186537 | 100 | Soil | China |
| SRR8606973 | SRX5406528 | PRJNA523309 | SRP186537 | 100 | Soil | China |
| SRR8606971 | SRX5406530 | PRJNA523309 | SRP186537 | 100 | Soil | China |
| SRR8606970 | SRX5406531 | PRJNA523309 | SRP186537 | 100 | Soil | China |
| SRR8606969 | SRX5406532 | PRJNA523309 | SRP186537 | 100 | Soil | China |
| SRR8606968 | SRX5406533 | PRJNA523309 | SRP186537 | 100 | Soil | China |
| SRR8606967 | SRX5406534 | PRJNA523309 | SRP186537 | 100 | Soil | China |
| SRR8606966 | SRX5406535 | PRJNA523309 | SRP186537 | 100 | Soil | China |
| SRR8606962 | SRX5406539 | PRJNA523309 | SRP186537 | 100 | Soil | China |
| SRR8606960 | SRX5406541 | PRJNA523309 | SRP186537 | 99.58 | Soil | China |
| SRR8606955 | SRX5406546 | PRJNA523309 | SRP186537 | 100 | Soil | China |
| SRR8606954 | SRX5406547 | PRJNA523309 | SRP186537 | 100 | Soil | China |
| SRR8606953 | SRX5406548 | PRJNA523309 | SRP186537 | 100 | Soil | China |
| SRR8606952 | SRX5406549 | PRJNA523309 | SRP186537 | 100 | Soil | China |
| SRR8606951 | SRX5406550 | PRJNA523309 | SRP186537 | 100 | Soil | China |
| SRR8606950 | SRX5406551 | PRJNA523309 | SRP186537 | 100 | Soil | China |
| SRR8606949 | SRX5406552 | PRJNA523309 | SRP186537 | 100 | Soil | China |
| SRR8606946 | SRX5406555 | PRJNA523309 | SRP186537 | 100 | Soil | China |
| SRR8631575 | SRX5430358 | PRJNA523904 | SRP186832 | 99.66 | Soil | China |
| SRR8631574 | SRX5430359 | PRJNA523904 | SRP186832 | 99.66 | Soil | China |
| SRR8631573 | SRX5430360 | PRJNA523904 | SRP186832 | 99.66 | Soil | China |
| SRR8631572 | SRX5430361 | PRJNA523904 | SRP186832 | 99.66 | Soil | China |
| SRR8631571 | SRX5430362 | PRJNA523904 | SRP186832 | 99.66 | Soil | China |
| SRR8631570 | SRX5430363 | PRJNA523904 | SRP186832 | 99.66 | Soil | China |
| SRR8631569 | SRX5430364 | PRJNA523904 | SRP186832 | 99.66 | Soil | China |
| SRR8631568 | SRX5430365 | PRJNA523904 | SRP186832 | 100 | Soil | China |
| SRR8631567 | SRX5430366 | PRJNA523904 | SRP186832 | 99.66 | Soil | China |
| SRR8631566 | SRX5430367 | PRJNA523904 | SRP186832 | 99.66 | Soil | China |
| SRR8631565 | SRX5430368 | PRJNA523904 | SRP186832 | 99.66 | Soil | China |
| SRR8631564 | SRX5430369 | PRJNA523904 | SRP186832 | 99.66 | Soil | China |
| SRR8668031 | SRX5464046 | PRJNA525518 | SRP187488 | 99.58 | Soil | China |
| SRR8720022 | SRX5513820 | PRJNA526607 | SRP188304 | 99.59 | Rhizosphere | China |
| SRR8720019 | SRX5513823 | PRJNA526607 | SRP188304 | 100 | Rhizosphere | China |
| SRR8720016 | SRX5513826 | PRJNA526607 | SRP188304 | 99.59 | Rhizosphere | China |
| SRR8720015 | SRX5513827 | PRJNA526607 | SRP188304 | 100 | Rhizosphere | China |
| SRR8720014 | SRX5513828 | PRJNA526607 | SRP188304 | 100 | Rhizosphere | China |
| SRR8720012 | SRX5513830 | PRJNA526607 | SRP188304 | 100 | Rhizosphere | China |
| SRR8720011 | SRX5513831 | PRJNA526607 | SRP188304 | 99.59 | Rhizosphere | China |
| SRR8720010 | SRX5513832 | PRJNA526607 | SRP188304 | 100 | Rhizosphere | China |
| SRR8720009 | SRX5513833 | PRJNA526607 | SRP188304 | 100 | Rhizosphere | China |
| SRR8720006 | SRX5513836 | PRJNA526607 | SRP188304 | 99.59 | Rhizosphere | China |
| SRR8728632 | SRX5521684 | PRJNA527014 | SRP188423 | 99.18 | Rhizosphere | China |
| SRR8728629 | SRX5521687 | PRJNA527014 | SRP188423 | 99.18 | Rhizosphere | China |
| SRR8863849 | SRX5651263 | PRJNA531358 | SRP191361 | 99.6 | Soil | China |
| SRR8863848 | SRX5651264 | PRJNA531358 | SRP191361 | 99.2 | Soil | China |
| SRR8886849 | SRX5672565 | PRJNA532517 | SRP192280 | 99.66 | Soil | China |
| SRR8933044 | SRX5713690 | PRJNA533781 | SRP193120 | 99.58 | Soil | China |
| SRR8988998 | SRX5768117 | PRJNA540415 | SRP194227 | 99.66 | Soil | China |
| SRR9056834 | SRX5833061 | PRJNA542990 | SRP198434 | 99.66 | Soil | China |
| SRR9056833 | SRX5833062 | PRJNA542990 | SRP198434 | 99.66 | Soil | China |
| SRR9056830 | SRX5833065 | PRJNA542990 | SRP198434 | 99.66 | Soil | China |
| SRR9153719 | SRX5926484 | PRJNA544819 | SRP199784 | 99.58 | Soil | China |
| SRR9153716 | SRX5926487 | PRJNA544819 | SRP199784 | 99.58 | Soil | China |
| SRR9153702 | SRX5926501 | PRJNA544819 | SRP199784 | 99.58 | Soil | China |
| SRR9153688 | SRX5926515 | PRJNA544819 | SRP199784 | 99.58 | Soil | China |
| SRR9153686 | SRX5926517 | PRJNA544819 | SRP199784 | 100 | Soil | China |
| SRR9153653 | SRX5926550 | PRJNA544819 | SRP199784 | 99.58 | Soil | China |
| SRR9153652 | SRX5926551 | PRJNA544819 | SRP199784 | 99.58 | Soil | China |
| SRR9153593 | SRX5926610 | PRJNA544819 | SRP199784 | 99.58 | Soil | China |
| SRR9153581 | SRX5926622 | PRJNA544819 | SRP199784 | 99.16 | Soil | China |
| SRR9153565 | SRX5926638 | PRJNA544819 | SRP199784 | 99.16 | Soil | China |
| SRR9153563 | SRX5926640 | PRJNA544819 | SRP199784 | 99.58 | Soil | China |
| SRR9153550 | SRX5926653 | PRJNA544819 | SRP199784 | 99.16 | Soil | China |
| SRR9153455 | SRX5926748 | PRJNA544819 | SRP199784 | 99.58 | Soil | China |
| SRR9153454 | SRX5926749 | PRJNA544819 | SRP199784 | 99.58 | Soil | China |
| SRR9153432 | SRX5926771 | PRJNA544819 | SRP199784 | 99.58 | Soil | China |
| SRR9153431 | SRX5926772 | PRJNA544819 | SRP199784 | 99.58 | Soil | China |
| SRR9153414 | SRX5926789 | PRJNA544819 | SRP199784 | 99.58 | Soil | China |
| SRR9153411 | SRX5926792 | PRJNA544819 | SRP199784 | 100 | Soil | China |
| SRR9153779 | SRX5926814 | PRJNA544819 | SRP199784 | 99.61 | Soil | China |
| SRR9153766 | SRX5926827 | PRJNA544819 | SRP199784 | 100 | Soil | China |
| SRR9153759 | SRX5926834 | PRJNA544819 | SRP199784 | 99.58 | Soil | China |
| SRR9154099 | SRX5926884 | PRJNA544819 | SRP199784 | 99.58 | Soil | China |
| SRR9154074 | SRX5926909 | PRJNA544819 | SRP199784 | 99.16 | Soil | China |
| SRR9153972 | SRX5927011 | PRJNA544819 | SRP199784 | 99.58 | Soil | China |
| SRR9153968 | SRX5927015 | PRJNA544819 | SRP199784 | 100 | Soil | China |
| SRR9153953 | SRX5927030 | PRJNA544819 | SRP199784 | 99.58 | Soil | China |
| SRR9153945 | SRX5927038 | PRJNA544819 | SRP199784 | 99.58 | Soil | China |
| SRR9153911 | SRX5927072 | PRJNA544819 | SRP199784 | 99.58 | Soil | China |
| SRR9153857 | SRX5927126 | PRJNA544819 | SRP199784 | 99.16 | Soil | China |
| SRR9153852 | SRX5927131 | PRJNA544819 | SRP199784 | 100 | Soil | China |
| SRR9153848 | SRX5927135 | PRJNA544819 | SRP199784 | 99.58 | Soil | China |
| SRR9153840 | SRX5927143 | PRJNA544819 | SRP199784 | 99.58 | Soil | China |
| SRR9153815 | SRX5927168 | PRJNA544819 | SRP199784 | 99.58 | Soil | China |
| SRR9153806 | SRX5927177 | PRJNA544819 | SRP199784 | 99.58 | Soil | China |
| SRR9153790 | SRX5927193 | PRJNA544819 | SRP199784 | 99.58 | Soil | China |
| SRR9226045 | SRX5996476 | PRJNA547973 | SRP200912 | 99.16 | Soil | China |
| SRR9226031 | SRX5996490 | PRJNA547973 | SRP200912 | 100 | Soil | China |
| SRR9226005 | SRX5996516 | PRJNA547973 | SRP200912 | 100 | Soil | China |
| SRR9225997 | SRX5996524 | PRJNA547973 | SRP200912 | 99.57 | Soil | China |
| SRR9225979 | SRX5996542 | PRJNA547973 | SRP200912 | 99.58 | Soil | China |
| SRR9225976 | SRX5996545 | PRJNA547973 | SRP200912 | 100 | Soil | China |
| SRR9225975 | SRX5996546 | PRJNA547973 | SRP200912 | 99.16 | Soil | China |
| SRR9586961 | SRX6352794 | PRJNA550293 | SRP211774 | 100 | Soil | China |
| SRR9614818 | SRX6377885 | PRJNA551928 | SRP212377 | 99.67 | Soil | China |
| SRR9654217 | SRX6415594 | PRJNA552950 | SRP213681 | 99.2 | Early phase of fermentation | China |
| SRR5179583 | SRX6622645 | PRJNA361542 | SRP096858 | 99.6 | Soil | China |
| SRR5179586 | SRX6622648 | PRJNA361542 | SRP096858 | 99.6 | Soil | China |
| SRR5179587 | SRX6622649 | PRJNA361542 | SRP096858 | 99.6 | Soil | China |
| SRR5179588 | SRX6622650 | PRJNA361542 | SRP096858 | 99.6 | Soil | China |
| SRR9892305 | SRX6644725 | PRJNA558207 | SRP217188 | 99.58 | Rhizosphere | China |
| SRR9951917 | SRX6700264 | PRJNA559786 | SRP218116 | 99.6 | Soil | China |
| SRR9951916 | SRX6700265 | PRJNA559786 | SRP218116 | 100 | Soil | China |
| SRR9951915 | SRX6700266 | PRJNA559786 | SRP218116 | 99.6 | Soil | China |
| SRR9951914 | SRX6700267 | PRJNA559786 | SRP218116 | 99.6 | Soil | China |
| SRR9951913 | SRX6700268 | PRJNA559786 | SRP218116 | 100 | Soil | China |
| SRR9951912 | SRX6700269 | PRJNA559786 | SRP218116 | 100 | Soil | China |
| SRR9951911 | SRX6700270 | PRJNA559786 | SRP218116 | 100 | Soil | China |
| SRR9951910 | SRX6700271 | PRJNA559786 | SRP218116 | 100 | Soil | China |
| SRR9951909 | SRX6700272 | PRJNA559786 | SRP218116 | 100 | Soil | China |
| SRR9951908 | SRX6700273 | PRJNA559786 | SRP218116 | 100 | Soil | China |
| SRR9951905 | SRX6700276 | PRJNA559786 | SRP218116 | 100 | Soil | China |
| SRR9951904 | SRX6700277 | PRJNA559786 | SRP218116 | 100 | Soil | China |
| SRR9951903 | SRX6700278 | PRJNA559786 | SRP218116 | 100 | Soil | China |
| SRR9951902 | SRX6700279 | PRJNA559786 | SRP218116 | 100 | Soil | China |
| SRR9951901 | SRX6700280 | PRJNA559786 | SRP218116 | 99.6 | Soil | China |
| SRR9951900 | SRX6700281 | PRJNA559786 | SRP218116 | 99.6 | Soil | China |
| SRR9951899 | SRX6700282 | PRJNA559786 | SRP218116 | 100 | Soil | China |
| SRR9951898 | SRX6700283 | PRJNA559786 | SRP218116 | 100 | Soil | China |
| SRR9951897 | SRX6700284 | PRJNA559786 | SRP218116 | 99.6 | Soil | China |
| SRR9994441 | SRX6733603 | PRJNA560457 | SRP218526 | 99.6 | Soil | China |
| SRR9994440 | SRX6733604 | PRJNA560457 | SRP218526 | 99.2 | Soil | China |
| SRR10005357 | SRX6743698 | PRJNA560738 | SRP218883 | 99.32 | Rhizosphere | China |
| SRR10237401 | SRX6956599 | PRJNA576133 | SRP224567 | 99.64 | Soil | China |
| SRR10237376 | SRX6956624 | PRJNA576133 | SRP224567 | 100 | Soil | China |
| SRR10237375 | SRX6956625 | PRJNA576133 | SRP224567 | 100 | Soil | China |
| SRR10237374 | SRX6956626 | PRJNA576133 | SRP224567 | 99.64 | Soil | China |
| SRR10269655 | SRX6982966 | PRJNA576976 | SRP225248 | 99.32 | Soil | China |
| SRR10317082 | SRX7028015 | PRJNA578172 | SRP226441 | 99.59 | Soil | China |
| SRR10317080 | SRX7028017 | PRJNA578172 | SRP226441 | 100 | Soil | China |
| SRR10337016 | SRX7047081 | PRJNA578999 | SRP226734 | 99.18 | Soil | China |
| SRR10359364 | SRX7068755 | PRJNA580189 | SRP227328 | 99.6 | Rhizosphere | China |
| SRR10359363 | SRX7068756 | PRJNA580189 | SRP227328 | 99.2 | Rhizosphere | China |
| SRR10359362 | SRX7068757 | PRJNA580189 | SRP227328 | 99.6 | Rhizosphere | China |
| SRR10359356 | SRX7068763 | PRJNA580189 | SRP227328 | 100 | Rhizosphere | China |
| SRR10439167 | SRX7134906 | PRJNA589461 | SRP229707 | 99.6 | Soil | China |
| SRR10439164 | SRX7134909 | PRJNA589461 | SRP229707 | 100 | Soil | China |
| SRR10439161 | SRX7134912 | PRJNA589461 | SRP229707 | 99.6 | Soil | China |
| SRR10439157 | SRX7134916 | PRJNA589461 | SRP229707 | 100 | Soil | China |
| SRR10439154 | SRX7134919 | PRJNA589461 | SRP229707 | 100 | Soil | China |
| SRR10483313 | SRX7173004 | PRJNA590071 | SRP230347 | 100 | Sediment | China |
| SRR10483308 | SRX7173009 | PRJNA590071 | SRP230347 | 99.14 | Sediment | China |
| SRR10483307 | SRX7173010 | PRJNA590071 | SRP230347 | 100 | Sediment | China |
| SRR10483306 | SRX7173011 | PRJNA590071 | SRP230347 | 100 | Sediment | China |
| SRR10483300 | SRX7173017 | PRJNA590071 | SRP230347 | 99.71 | Sediment | China |
| SRR10483296 | SRX7173021 | PRJNA590071 | SRP230347 | 100 | Sediment | China |
| SRR10483293 | SRX7173024 | PRJNA590071 | SRP230347 | 99.71 | Sediment | China |
| SRR10513701 | SRX7201817 | PRJNA591219 | SRP231332 | 99.66 | Soil | China |
| SRR10550140 | SRX7232350 | PRJNA592049 | SRP233581 | 100 | Soil | China |
| SRR10550139 | SRX7232351 | PRJNA592049 | SRP233581 | 100 | Soil | China |
| SRR10550137 | SRX7232353 | PRJNA592049 | SRP233581 | 100 | Soil | China |
| SRR10550135 | SRX7232355 | PRJNA592049 | SRP233581 | 100 | Soil | China |
| SRR10550134 | SRX7232356 | PRJNA592049 | SRP233581 | 100 | Soil | China |
| SRR10561075 | SRX7242694 | PRJNA592717 | SRP234066 | 99.6 | Soil | China |
| SRR10561065 | SRX7242704 | PRJNA592717 | SRP234066 | 99.6 | Soil | China |
| SRR10561063 | SRX7242706 | PRJNA592717 | SRP234066 | 99.6 | Soil | China |
| SRR10561060 | SRX7242709 | PRJNA592717 | SRP234066 | 99.6 | Soil | China |
| SRR10561052 | SRX7242717 | PRJNA592717 | SRP234066 | 99.6 | Soil | China |
| SRR10799547 | SRX7473500 | PRJNA597970 | SRP238957 | 99.6 | Soil | China |
| SRR10820212 | SRX7493703 | PRJNA598558 | SRP239309 | 100 | Rhizosphere | China |
| SRR10820206 | SRX7493709 | PRJNA598558 | SRP239309 | 100 | Rhizosphere | China |
| SRR10820200 | SRX7493715 | PRJNA598558 | SRP239309 | 100 | Rhizosphere | China |
| SRR10820198 | SRX7493717 | PRJNA598558 | SRP239309 | 100 | Rhizosphere | China |
| SRR1661391 | SRX766522 | PRJNA268306 | SRP050184 | 100 | Soil | China |
| SRR1661405 | SRX766525 | PRJNA268306 | SRP050184 | 100 | Soil | China |
| SRR11067060 | SRX7707462 | PRJNA606021 | SRP248190 | 99.58 | Soil | China |
| SRR11146056 | SRX7782197 | PRJNA608072 | SRP250361 | 100 | Soil | China |
| SRR11146054 | SRX7782199 | PRJNA608072 | SRP250361 | 100 | Soil | China |
| SRR11216091 | SRX7828336 | PRJNA609767 | SRP251305 | 99.66 | Soil | China |
| SRR11216089 | SRX7828338 | PRJNA609767 | SRP251305 | 99.66 | Soil | China |
| SRR11286213 | SRX7891905 | PRJNA611692 | SRP252348 | 99.6 | Soil | China |
| SRR11286209 | SRX7891909 | PRJNA611692 | SRP252348 | 99.6 | Soil | China |
| SRR11286205 | SRX7891913 | PRJNA611692 | SRP252348 | 99.6 | Soil | China |
| SRR11310249 | SRX7914783 | PRJNA612659 | SRP252911 | 99.6 | Rhizosphere | China |
| SRR11310245 | SRX7914787 | PRJNA612659 | SRP252911 | 99.6 | Rhizosphere | China |
| SRR11310244 | SRX7914788 | PRJNA612659 | SRP252911 | 99.6 | Rhizosphere | China |
| SRR11310243 | SRX7914789 | PRJNA612659 | SRP252911 | 99.6 | Rhizosphere | China |
| SRR11310242 | SRX7914790 | PRJNA612659 | SRP252911 | 100 | Rhizosphere | China |
| SRR11310241 | SRX7914791 | PRJNA612659 | SRP252911 | 99.6 | Rhizosphere | China |
| SRR11311106 | SRX7915612 | PRJNA612600 | SRP252944 | 100 | Mangrove | China |
| SRR11311104 | SRX7915614 | PRJNA612600 | SRP252944 | 99.6 | Mangrove | China |
| SRR11392206 | SRX7971321 | PRJNA613792 | SRP253708 | 100 | Mangrove | China |
| SRR11508497 | SRX8080636 | PRJNA623514 | SRP255673 | 99.58 | Soil | China |
| SRR11515685 | SRX8087201 | PRJNA623987 | SRP255814 | 100 | Sewage sludge | China |
| SRR11515683 | SRX8087203 | PRJNA623987 | SRP255814 | 100 | Sewage sludge | China |
| SRR11515681 | SRX8087205 | PRJNA623987 | SRP255814 | 100 | Sewage sludge | China |
| SRR11515677 | SRX8087209 | PRJNA623987 | SRP255814 | 99.6 | Sewage sludge | China |
| SRR11515672 | SRX8087214 | PRJNA623987 | SRP255814 | 100 | Sewage sludge | China |
| SRR11515669 | SRX8087217 | PRJNA623987 | SRP255814 | 100 | Sewage sludge | China |
| SRR11515668 | SRX8087218 | PRJNA623987 | SRP255814 | 100 | Sewage sludge | China |
| SRR11515664 | SRX8087222 | PRJNA623987 | SRP255814 | 99.57 | Sewage sludge | China |
| SRR11515659 | SRX8087227 | PRJNA623987 | SRP255814 | 100 | Sewage sludge | China |
| SRR11515657 | SRX8087229 | PRJNA623987 | SRP255814 | 100 | Sewage sludge | China |
| SRR11515648 | SRX8087238 | PRJNA623987 | SRP255814 | 99.15 | Sewage sludge | China |
| SRR11515642 | SRX8087244 | PRJNA623987 | SRP255814 | 99.26 | Sewage sludge | China |
| SRR11515640 | SRX8087246 | PRJNA623987 | SRP255814 | 99.6 | Sewage sludge | China |
| SRR11515632 | SRX8087254 | PRJNA623987 | SRP255814 | 100 | Sewage sludge | China |
| SRR11515631 | SRX8087255 | PRJNA623987 | SRP255814 | 100 | Sewage sludge | China |
| SRR11515627 | SRX8087259 | PRJNA623987 | SRP255814 | 100 | Sewage sludge | China |
| SRR11515626 | SRX8087260 | PRJNA623987 | SRP255814 | 100 | Sewage sludge | China |
| SRR11515625 | SRX8087261 | PRJNA623987 | SRP255814 | 99.6 | Sewage sludge | China |
| SRR11515622 | SRX8087264 | PRJNA623987 | SRP255814 | 100 | Sewage sludge | China |
| SRR11515620 | SRX8087266 | PRJNA623987 | SRP255814 | 100 | Sewage sludge | China |
| SRR11515618 | SRX8087268 | PRJNA623987 | SRP255814 | 100 | Sewage sludge | China |
| SRR11515614 | SRX8087272 | PRJNA623987 | SRP255814 | 100 | Sewage sludge | China |
| SRR11515613 | SRX8087273 | PRJNA623987 | SRP255814 | 99.58 | Sewage sludge | China |
| SRR11515610 | SRX8087276 | PRJNA623987 | SRP255814 | 100 | Sewage sludge | China |
| SRR11515609 | SRX8087277 | PRJNA623987 | SRP255814 | 100 | Sewage sludge | China |
| SRR11515606 | SRX8087280 | PRJNA623987 | SRP255814 | 99.6 | Sewage sludge | China |
| SRR11515604 | SRX8087282 | PRJNA623987 | SRP255814 | 100 | Sewage sludge | China |
| SRR11515602 | SRX8087284 | PRJNA623987 | SRP255814 | 100 | Sewage sludge | China |
| SRR11515600 | SRX8087286 | PRJNA623987 | SRP255814 | 100 | Sewage sludge | China |
| SRR11515598 | SRX8087288 | PRJNA623987 | SRP255814 | 100 | Sewage sludge | China |
| SRR11515593 | SRX8087293 | PRJNA623987 | SRP255814 | 100 | Sewage sludge | China |
| SRR11515591 | SRX8087295 | PRJNA623987 | SRP255814 | 100 | Sewage sludge | China |
| SRR11537001 | SRX8108085 | PRJNA625153 | SRP256169 | 99.32 | Soil | China |
| SRR11536998 | SRX8108088 | PRJNA625153 | SRP256169 | 99.65 | Soil | China |
| SRR11565382 | SRX8134531 | PRJNA625866 | SRP257008 | 99.32 | Soil | China |
| SRR11570200 | SRX8138548 | PRJNA626395 | SRP257427 | 99.66 | Human lung | China |
| SRR11570198 | SRX8138550 | PRJNA626395 | SRP257427 | 99.66 | Human lung | China |
| SRR11570738 | SRX8138961 | PRJNA626504 | SRP257455 | 99.67 | Sediment | China |
| SRR11570710 | SRX8138989 | PRJNA626504 | SRP257455 | 100 | Sediment | China |
| SRR11570633 | SRX8139066 | PRJNA626504 | SRP257455 | 99 | Sediment | China |
| SRR11570621 | SRX8139078 | PRJNA626504 | SRP257455 | 99.33 | Sediment | China |
| SRR11570620 | SRX8139079 | PRJNA626504 | SRP257455 | 100 | Sediment | China |
| SRR11742992 | SRX8296396 | PRJNA631323 | SRP260858 | 99.66 | Soil | China |
| SRR11742984 | SRX8296404 | PRJNA631323 | SRP260858 | 99.66 | Soil | China |
| SRR11742983 | SRX8296405 | PRJNA631323 | SRP260858 | 99.66 | Soil | China |
| SRR11742971 | SRX8296417 | PRJNA631323 | SRP260858 | 99.66 | Soil | China |
| SRR11782045 | SRX8334496 | PRJNA632354 | SRP261363 | 99.2 | Soil | China |
| SRR11782106 | SRX8334534 | PRJNA632431 | SRP261370 | 99.6 | Soil | China |
| SRR11782105 | SRX8334535 | PRJNA632431 | SRP261370 | 99.6 | Soil | China |
| SRR11782104 | SRX8334536 | PRJNA632431 | SRP261370 | 99.6 | Soil | China |
| SRR11782103 | SRX8334537 | PRJNA632431 | SRP261370 | 99.6 | Soil | China |
| SRR11782102 | SRX8334538 | PRJNA632431 | SRP261370 | 99.6 | Soil | China |
| SRR11782101 | SRX8334539 | PRJNA632431 | SRP261370 | 99.6 | Soil | China |
| SRR11782100 | SRX8334540 | PRJNA632431 | SRP261370 | 99.6 | Soil | China |
| SRR11782099 | SRX8334541 | PRJNA632431 | SRP261370 | 99.6 | Soil | China |
| SRR11782098 | SRX8334542 | PRJNA632431 | SRP261370 | 99.6 | Soil | China |
| SRR11782097 | SRX8334543 | PRJNA632431 | SRP261370 | 99.6 | Soil | China |
| SRR11782096 | SRX8334544 | PRJNA632431 | SRP261370 | 99.6 | Soil | China |
| SRR11782094 | SRX8334546 | PRJNA632431 | SRP261370 | 99.6 | Soil | China |
| SRR11782093 | SRX8334547 | PRJNA632431 | SRP261370 | 100 | Soil | China |
| SRR11782091 | SRX8334549 | PRJNA632431 | SRP261370 | 99.6 | Soil | China |
| SRR11782090 | SRX8334550 | PRJNA632431 | SRP261370 | 99.6 | Soil | China |
| SRR11782089 | SRX8334551 | PRJNA632431 | SRP261370 | 99.6 | Soil | China |
| SRR11782088 | SRX8334552 | PRJNA632431 | SRP261370 | 99.6 | Soil | China |
| SRR11782087 | SRX8334553 | PRJNA632431 | SRP261370 | 99.6 | Soil | China |
| SRR11782086 | SRX8334554 | PRJNA632431 | SRP261370 | 99.6 | Soil | China |
| SRR11782085 | SRX8334555 | PRJNA632431 | SRP261370 | 99.6 | Soil | China |
| SRR11782084 | SRX8334556 | PRJNA632431 | SRP261370 | 99.6 | Soil | China |
| SRR11782083 | SRX8334557 | PRJNA632431 | SRP261370 | 99.6 | Soil | China |
| SRR11782082 | SRX8334558 | PRJNA632431 | SRP261370 | 99.6 | Soil | China |
| SRR11782081 | SRX8334559 | PRJNA632431 | SRP261370 | 99.6 | Soil | China |
| SRR11782394 | SRX8334845 | PRJNA632441 | SRP261377 | 99.2 | Soil | China |
| SRR11789206 | SRX8341299 | PRJNA632758 | SRP261578 | 99.6 | Soil | China |
| SRR11789204 | SRX8341301 | PRJNA632758 | SRP261578 | 99.6 | Soil | China |
| SRR11789203 | SRX8341302 | PRJNA632758 | SRP261578 | 99.6 | Soil | China |
| SRR11789202 | SRX8341303 | PRJNA632758 | SRP261578 | 99.6 | Soil | China |
| SRR11789201 | SRX8341304 | PRJNA632758 | SRP261578 | 99.6 | Soil | China |
| SRR11789200 | SRX8341305 | PRJNA632758 | SRP261578 | 99.6 | Soil | China |
| SRR11789199 | SRX8341306 | PRJNA632758 | SRP261578 | 99.6 | Soil | China |
| SRR11789198 | SRX8341307 | PRJNA632758 | SRP261578 | 99.6 | Soil | China |
| SRR11789197 | SRX8341308 | PRJNA632758 | SRP261578 | 99.6 | Soil | China |
| SRR11789196 | SRX8341309 | PRJNA632758 | SRP261578 | 100 | Soil | China |
| SRR11789195 | SRX8341310 | PRJNA632758 | SRP261578 | 99.6 | Soil | China |
| SRR11789194 | SRX8341311 | PRJNA632758 | SRP261578 | 99.21 | Soil | China |
| SRR11789193 | SRX8341312 | PRJNA632758 | SRP261578 | 99.21 | Soil | China |
| SRR11789192 | SRX8341313 | PRJNA632758 | SRP261578 | 99.6 | Soil | China |
| SRR11789191 | SRX8341314 | PRJNA632758 | SRP261578 | 99.6 | Soil | China |
| SRR11789190 | SRX8341315 | PRJNA632758 | SRP261578 | 99.6 | Soil | China |
| SRR11789188 | SRX8341317 | PRJNA632758 | SRP261578 | 99.6 | Soil | China |
| SRR11789187 | SRX8341318 | PRJNA632758 | SRP261578 | 99.6 | Soil | China |
| SRR11789186 | SRX8341319 | PRJNA632758 | SRP261578 | 100 | Soil | China |
| SRR11789185 | SRX8341320 | PRJNA632758 | SRP261578 | 99.6 | Soil | China |
| SRR11789184 | SRX8341321 | PRJNA632758 | SRP261578 | 99.6 | Soil | China |
| SRR11789183 | SRX8341322 | PRJNA632758 | SRP261578 | 99.6 | Soil | China |
| SRR11789182 | SRX8341323 | PRJNA632758 | SRP261578 | 99.6 | Soil | China |
| SRR11789181 | SRX8341324 | PRJNA632758 | SRP261578 | 99.6 | Soil | China |
| SRR11789180 | SRX8341325 | PRJNA632758 | SRP261578 | 100 | Soil | China |
| SRR11833743 | SRX8384074 | PRJNA634574 | SRP263040 | 99.6 | Soil | China |
| SRR11833731 | SRX8384086 | PRJNA634574 | SRP263040 | 99.6 | Soil | China |
| SRR11833711 | SRX8384106 | PRJNA634574 | SRP263040 | 99.59 | Soil | China |
| SRR11833694 | SRX8384123 | PRJNA634574 | SRP263040 | 99.6 | Soil | China |
| SRR11833670 | SRX8384148 | PRJNA634574 | SRP263040 | 99.6 | Soil | China |
| SRR11833661 | SRX8384150 | PRJNA634574 | SRP263040 | 99.6 | Soil | China |
| SRR11833660 | SRX8384157 | PRJNA634574 | SRP263040 | 99.6 | Soil | China |
| SRR11844258 | SRX8394610 | PRJNA634903 | SRP263544 | 100 | Soil | China |
| SRR11846036 | SRX8396379 | PRJNA634967 | SRP264208 | 100 | Soil | China |
| SRR11846035 | SRX8396380 | PRJNA634967 | SRP264208 | 100 | Soil | China |
| SRR11846033 | SRX8396382 | PRJNA634967 | SRP264208 | 100 | Soil | China |
| SRR11846028 | SRX8396387 | PRJNA634967 | SRP264208 | 100 | Soil | China |
| SRR11846025 | SRX8396390 | PRJNA634967 | SRP264208 | 100 | Soil | China |
| SRR11846023 | SRX8396392 | PRJNA634967 | SRP264208 | 100 | Soil | China |
| SRR11846014 | SRX8396401 | PRJNA634967 | SRP264208 | 100 | Soil | China |
| SRR11910037 | SRX8456628 | PRJNA636844 | SRP265680 | 99.15 | Soil | China |
| SRR11943197 | SRX8487649 | PRJNA637863 | SRP266297 | 99.58 | Soil | China |
| SRR11943196 | SRX8487650 | PRJNA637863 | SRP266297 | 99.58 | Soil | China |
| SRR11943154 | SRX8487692 | PRJNA637863 | SRP266297 | 99.58 | Soil | China |
| SRR11972483 | SRX8516252 | PRJNA638514 | SRP266714 | 100 | Soil | China |
| SRR12011178 | SRX8543830 | PRJNA639377 | SRP267257 | 99.63 | Rhizosphere | China |
| SRR12011176 | SRX8543832 | PRJNA639377 | SRP267257 | 99.63 | Rhizosphere | China |
| SRR12011171 | SRX8543837 | PRJNA639377 | SRP267257 | 99.26 | Rhizosphere | China |
| SRR12011167 | SRX8543841 | PRJNA639377 | SRP267257 | 100 | Rhizosphere | China |
| SRR12011166 | SRX8543842 | PRJNA639377 | SRP267257 | 99.63 | Rhizosphere | China |
| SRR12011162 | SRX8543846 | PRJNA639377 | SRP267257 | 99.63 | Rhizosphere | China |
| SRR12011133 | SRX8543875 | PRJNA639377 | SRP267257 | 100 | Rhizosphere | China |
| SRR12011132 | SRX8543876 | PRJNA639377 | SRP267257 | 100 | Rhizosphere | China |
| SRR12011131 | SRX8543877 | PRJNA639377 | SRP267257 | 99.63 | Rhizosphere | China |
| SRR12011130 | SRX8543878 | PRJNA639377 | SRP267257 | 99.63 | Rhizosphere | China |
| SRR12011129 | SRX8543879 | PRJNA639377 | SRP267257 | 100 | Rhizosphere | China |
| SRR12011100 | SRX8543908 | PRJNA639377 | SRP267257 | 99.26 | Rhizosphere | China |
| SRR12013088 | SRX8545742 | PRJNA639362 | SRP267303 | 99.6 | Soil | China |
| SRR12013087 | SRX8545743 | PRJNA639362 | SRP267303 | 99.6 | Soil | China |
| SRR12013023 | SRX8545807 | PRJNA639362 | SRP267303 | 99.6 | Soil | China |
| SRR12013019 | SRX8545811 | PRJNA639362 | SRP267303 | 99.6 | Soil | China |
| SRR12013017 | SRX8545813 | PRJNA639362 | SRP267303 | 100 | Soil | China |
| SRR12031844 | SRX8562849 | PRJNA639921 | SRP267612 | 99.66 | Soil | China |
| SRR12031843 | SRX8562850 | PRJNA639921 | SRP267612 | 99.32 | Soil | China |
| SRR12031833 | SRX8562860 | PRJNA639921 | SRP267612 | 99.66 | Soil | China |
| SRR12031801 | SRX8562892 | PRJNA639921 | SRP267612 | 99.66 | Soil | China |
| SRR12031788 | SRX8562905 | PRJNA639921 | SRP267612 | 99.32 | Soil | China |
| SRR12031785 | SRX8562908 | PRJNA639921 | SRP267612 | 99.32 | Soil | China |
| SRR12031779 | SRX8562914 | PRJNA639921 | SRP267612 | 99.66 | Soil | China |
| SRR12031778 | SRX8562915 | PRJNA639921 | SRP267612 | 99.66 | Soil | China |
| SRR12031754 | SRX8562939 | PRJNA639921 | SRP267612 | 99.66 | Soil | China |
| SRR12031745 | SRX8562948 | PRJNA639921 | SRP267612 | 99.66 | Soil | China |
| SRR12031738 | SRX8562955 | PRJNA639921 | SRP267612 | 99.66 | Soil | China |
| SRR12031736 | SRX8562957 | PRJNA639921 | SRP267612 | 99.66 | Soil | China |
| SRR12031735 | SRX8562958 | PRJNA639921 | SRP267612 | 99.66 | Soil | China |
| SRR12031719 | SRX8562974 | PRJNA639921 | SRP267612 | 99.32 | Soil | China |
| SRR12031702 | SRX8562991 | PRJNA639921 | SRP267612 | 99.66 | Soil | China |
| SRR12031681 | SRX8563012 | PRJNA639921 | SRP267612 | 99.32 | Soil | China |
| SRR12031652 | SRX8563041 | PRJNA639921 | SRP267612 | 99.32 | Soil | China |
| SRR12031645 | SRX8563048 | PRJNA639921 | SRP267612 | 99.32 | Soil | China |
| SRR12031604 | SRX8563089 | PRJNA639921 | SRP267612 | 99.66 | Soil | China |
| SRR12031591 | SRX8563102 | PRJNA639921 | SRP267612 | 99.32 | Soil | China |
| SRR12031542 | SRX8563151 | PRJNA639921 | SRP267612 | 99.66 | Soil | China |
| SRR12061690 | SRX8589584 | PRJNA640836 | SRP268161 | 99.63 | Soil | China |
| SRR12131226 | SRX8652436 | PRJNA643394 | SRP269644 | 99.66 | Soil | China |
| SRR12131223 | SRX8652439 | PRJNA643394 | SRP269644 | 99.32 | Soil | China |
| SRR12163060 | SRX8678534 | PRJNA644592 | SRP271949 | 99.63 | Straw residue | China |
| SRR12175307 | SRX8689976 | PRJNA644643 | SRP270966 | 99.66 | Soil | China |
| SRR12175306 | SRX8689977 | PRJNA644643 | SRP270966 | 99.66 | Soil | China |
| SRR12175287 | SRX8689996 | PRJNA644643 | SRP270966 | 99.66 | Soil | China |
| SRR12175285 | SRX8689998 | PRJNA644643 | SRP270966 | 99.66 | Soil | China |
| SRR12175284 | SRX8689999 | PRJNA644643 | SRP270966 | 99.66 | Soil | China |
| SRR12175267 | SRX8690016 | PRJNA644643 | SRP270966 | 99.66 | Soil | China |
| SRR12175259 | SRX8690024 | PRJNA644643 | SRP270966 | 99.32 | Soil | China |
| SRR12233733 | SRX8742267 | PRJNA646534 | SRP272170 | 99.66 | Soil | China |
| SRR12233732 | SRX8742268 | PRJNA646534 | SRP272170 | 99.66 | Soil | China |
| SRR12233731 | SRX8742269 | PRJNA646534 | SRP272170 | 99.66 | Soil | China |
| SRR3455730 | SRX1731576 | PRJNA317932 | SRP074055 | 100 | Soil | Christmas Island |
| SRR8845991 | SRX5633630 | PRJNA530637 | SRP190635 | 100 | Rhizosphere | Costa Rica |
| SRR10391597 | SRX7091933 | PRJNA587449 | SRP228311 | 99.32 | Soil | Czech Republic |
| SRR10391882 | SRX7092335 | PRJNA587449 | SRP228311 | 99.32 | Soil | Czech Republic |
| SRR12185630 | SRX8700225 | PRJNA645139 | SRP271123 | 100 | Soil | Czech Republic |
| SRR12185629 | SRX8700226 | PRJNA645139 | SRP271123 | 99.66 | Soil | Czech Republic |
| SRR9933834 | SRX6682626 | PRJNA558046 | SRP217857 | 100 | Soil | El Salvador |
| SRR9933806 | SRX6682654 | PRJNA558046 | SRP217857 | 100 | Soil | El Salvador |
| SRR4054372 | SRX2043947 | PRJNA339869 | SRP082695 | 99.2 | Soil | Finland |
| SRR6319604 | SRX3419494 | PRJNA419286 | SRP125392 | 99.32 | Sediment | Finland |
| SRR6319582 | SRX3419516 | PRJNA419286 | SRP125392 | 99.66 | Sediment | Finland |
| SRR6319581 | SRX3419517 | PRJNA419286 | SRP125392 | 100 | Sediment | Finland |
| SRR6319576 | SRX3419522 | PRJNA419286 | SRP125392 | 99.66 | Sediment | Finland |
| SRR9177015 | SRX5949671 | PRJNA545795 | SRP200087 | 99.66 | Sewage sludge | Finland |
| SRR11922728 | SRX8468447 | PRJNA637213 | SRP265901 | 99.34 | Soil | Finland |
| SRR11922719 | SRX8468456 | PRJNA637213 | SRP265901 | 99.66 | Soil | Finland |
| SRR6686004 | SRX3662219 | PRJNA433469 | SRP132446 | 100 | Human lung | France |
| SRR7773229 | SRX4628583 | PRJNA497210 | SRP165978 | 99.67 | Soil | France |
| SRR7773228 | SRX4628584 | PRJNA497210 | SRP165978 | 99.67 | Soil | France |
| ERR1934107 | ERX1991834 | PRJEB20391 | ERP022541 | 100 | Freshwater | Germany |
| ERR1934138 | ERX1991865 | PRJEB20391 | ERP022541 | 100 | Freshwater | Germany |
| ERR2223075 | ERX2276447 | PRJEB23949 | ERP105731 | 99.62 | Soil | Germany |
| ERR2223078 | ERX2276450 | PRJEB23949 | ERP105731 | 99.16 | Soil | Germany |
| ERR2223109 | ERX2276481 | PRJEB23949 | ERP105731 | 99.24 | Rhizosphere | Germany |
| ERR2818267 | ERX2825074 | PRJEB28861 | ERP111119 | 99.32 | Rhizosphere | Germany |
| SRR3440266 | SRX1726695 | PRJNA319487 | SRP073893 | 100 | Soil | Germany |
| SRR5575122 | SRX2833402 | PRJNA387117 | SRP107318 | 100 | Soil | Germany |
| SRR5575121 | SRX2833403 | PRJNA387117 | SRP107318 | 100 | Soil | Germany |
| SRR5575111 | SRX2833413 | PRJNA387117 | SRP107318 | 100 | Soil | Germany |
| SRR5575110 | SRX2833414 | PRJNA387117 | SRP107318 | 99.66 | Soil | Germany |
| SRR5575101 | SRX2833423 | PRJNA387117 | SRP107318 | 99.66 | Soil | Germany |
| SRR5575100 | SRX2833424 | PRJNA387117 | SRP107318 | 100 | Soil | Germany |
| SRR5575094 | SRX2833430 | PRJNA387117 | SRP107318 | 100 | Soil | Germany |
| SRR5575090 | SRX2833434 | PRJNA387117 | SRP107318 | 100 | Soil | Germany |
| SRR5577069 | SRX2835162 | PRJNA386947 | SRP107174 | 100 | Rhizosphere | Germany |
| SRR5576996 | SRX2835235 | PRJNA386947 | SRP107174 | 99.32 | Rhizosphere | Germany |
| SRR5576962 | SRX2835269 | PRJNA386947 | SRP107174 | 99.32 | Rhizosphere | Germany |
| SRR10364050 | SRX7072250 | PRJNA531340 | SRP227472 | 99.21 | Soil | Germany |
| SRR10364038 | SRX7072262 | PRJNA531340 | SRP227472 | 99.6 | Soil | Germany |
| SRR10364037 | SRX7072263 | PRJNA531340 | SRP227472 | 99.6 | Soil | Germany |
| SRR10364024 | SRX7072276 | PRJNA531340 | SRP227472 | 99.21 | Soil | Germany |
| SRR12089582 | SRX8615631 | PRJNA563995 | SRP268781 | 100 | Soil | Germany |
| SRR12089579 | SRX8615634 | PRJNA563995 | SRP268781 | 100 | Soil | Germany |
| SRR12089576 | SRX8615637 | PRJNA563995 | SRP268781 | 100 | Soil | Germany |
| SRR12089444 | SRX8615769 | PRJNA563995 | SRP268781 | 99.67 | Soil | Germany |
| SRR6201751 | SRX3311691 | PRJNA415280 | SRP120762 | 99.32 | Soil | Israel |
| ERR2211507 | ERX2265860 | PRJEB23682 | ERP105451 | 99.6 | Soil | Italy |
| SRR11058352 | SRX7704340 | PRJNA605669 | SRP248007 | 99.34 | Dust | Japan |
| ERR2190581 | ERX2246696 | PRJEB23318 | ERP105062 | 99 | Soil | Netherlands |
| ERR2673466 | ERX2688564 | PRJEB27512 | ERP109599 | 100 | Soil | Netherlands |
| ERR2673522 | ERX2688620 | PRJEB27512 | ERP109599 | 100 | Soil | Netherlands |
| ERR2673527 | ERX2688625 | PRJEB27512 | ERP109599 | 100 | Soil | Netherlands |
| ERR2673617 | ERX2688715 | PRJEB27512 | ERP109599 | 100 | Soil | Netherlands |
| ERR2673646 | ERX2688744 | PRJEB27512 | ERP109599 | 100 | Soil | Netherlands |
| ERR2673651 | ERX2688749 | PRJEB27512 | ERP109599 | 100 | Soil | Netherlands |
| ERR2777459 | ERX2789536 | PRJEB28463 | ERP110670 | 100 | Soil | Netherlands |
| ERR2777463 | ERX2789540 | PRJEB28463 | ERP110670 | 99.67 | Soil | Netherlands |
| ERR2777467 | ERX2789544 | PRJEB28463 | ERP110670 | 100 | Soil | Netherlands |
| ERR2777488 | ERX2789565 | PRJEB28463 | ERP110670 | 100 | Soil | Netherlands |
| ERR3507110 | ERX3528324 | PRJEB34236 | ERP117111 | 99.33 | Soil | Netherlands |
| ERR3507274 | ERX3528488 | PRJEB34236 | ERP117111 | 99.67 | Soil | Netherlands |
| SRR10136856 | SRX6865095 | PRJNA566105 | SRP222309 | 99.31 | Soil | Netherlands |
| SRR11603996 | SRX8170631 | PRJNA627834 | SRP258258 | 99.67 | Soil | Netherlands |
| SRR3724016 | SRX1881359 | PRJNA326983 | SRP077412 | 99.07 | Soil | New Zealand |
| ERR2211392 | ERX2265745 | PRJEB23682 | ERP105451 | 99.6 | Soil | Philippines |
| SRR12082391 | SRX8609487 | PRJNA641340 | SRP268453 | 99.33 | Soil | Portugal |
| SRR12082624 | SRX8609748 | PRJNA641340 | SRP268453 | 99.67 | Soil | Portugal |
| SRR8056368 | SRX4885927 | PRJNA362455 | SRP097606 | 100 | Rhizosphere | Reunion |
| SRR8056352 | SRX4885943 | PRJNA362455 | SRP097606 | 100 | Rhizosphere | Reunion |
| SRR9187851 | SRX5959801 | PRJNA545581 | SRP200241 | 99.61 | Mangrove | Singapore |
| SRR5320101 | SRX2619509 | PRJNA378134 | SRP101537 | 99.66 | Air samples | South Korea |
| SRR5320103 | SRX2619511 | PRJNA378134 | SRP101537 | 99.32 | Air samples | South Korea |
| SRR5685183 | SRX2919852 | PRJNA383959 | SRP109260 | 99.6 | Rhizosphere | South Korea |
| SRR5685179 | SRX2919856 | PRJNA383959 | SRP109260 | 100 | Rhizosphere | South Korea |
| SRR5685157 | SRX2919878 | PRJNA383959 | SRP109260 | 100 | Soil | South Korea |
| SRR5685156 | SRX2919879 | PRJNA383959 | SRP109260 | 100 | Soil | South Korea |
| SRR5685155 | SRX2919880 | PRJNA383959 | SRP109260 | 99.6 | Soil | South Korea |
| SRR5685154 | SRX2919881 | PRJNA383959 | SRP109260 | 99.6 | Soil | South Korea |
| SRR5685093 | SRX2919942 | PRJNA383959 | SRP109260 | 99.6 | Rhizosphere | South Korea |
| SRR5685077 | SRX2919958 | PRJNA383959 | SRP109260 | 100 | Rhizosphere | South Korea |
| SRR5685063 | SRX2919972 | PRJNA383959 | SRP109260 | 100 | Rhizosphere | South Korea |
| SRR5685062 | SRX2919973 | PRJNA383959 | SRP109260 | 100 | Rhizosphere | South Korea |
| SRR6126880 | SRX3239555 | PRJNA408181 | SRP119277 | 99.32 | Air samples | South Korea |
| SRR6488643 | SRX3578428 | PRJNA383959 | SRP109260 | 100 | Soil | South Korea |
| SRR6488642 | SRX3578429 | PRJNA383959 | SRP109260 | 100 | Soil | South Korea |
| SRR6488631 | SRX3578440 | PRJNA383959 | SRP109260 | 100 | Soil | South Korea |
| SRR6488627 | SRX3578444 | PRJNA383959 | SRP109260 | 100 | Soil | South Korea |
| SRR6488625 | SRX3578446 | PRJNA383959 | SRP109260 | 99.6 | Soil | South Korea |
| SRR6488600 | SRX3578471 | PRJNA383959 | SRP109260 | 99.59 | Soil | South Korea |
| SRR6488598 | SRX3578473 | PRJNA383959 | SRP109260 | 99.2 | Soil | South Korea |
| SRR10990839 | SRX7652214 | PRJNA603275 | SRP246172 | 100 | Soil | South Korea |
| SRR10990838 | SRX7652215 | PRJNA603275 | SRP246172 | 100 | Soil | South Korea |
| ERR952691 | ERX1031661 | PRJEB9654 | ERP010785 | 99.6 | Soil | Spain |
| ERR952703 | ERX1031673 | PRJEB9654 | ERP010785 | 99.2 | Soil | Spain |
| ERR952709 | ERX1031679 | PRJEB9654 | ERP010785 | 99.6 | Soil | Spain |
| SRR6293350 | SRX3394461 | PRJNA418454 | SRP125026 | 100 | Sewage sludge | Spain |
| SRR9112094 | SRX5886381 | PRJNA543961 | SRP199262 | 100 | Rhizosphere | Spain |
| SRR9112330 | SRX5886593 | PRJNA543961 | SRP199262 | 100 | Rhizosphere | Spain |
| SRR5519917 | SRX2793270 | PRJNA386024 | SRP106717 | 100 | Soil | Switzerland |
| SRR5519922 | SRX2793275 | PRJNA386024 | SRP106717 | 99.22 | Soil | Switzerland |
| SRR5519963 | SRX2793316 | PRJNA386024 | SRP106717 | 99.33 | Soil | Switzerland |
| SRR5519996 | SRX2793349 | PRJNA386024 | SRP106717 | 99.33 | Soil | Switzerland |
| SRR5520009 | SRX2793362 | PRJNA386024 | SRP106717 | 99.33 | Soil | Switzerland |
| SRR5520030 | SRX2793383 | PRJNA386024 | SRP106717 | 99.33 | Soil | Switzerland |
| SRR5520035 | SRX2793388 | PRJNA386024 | SRP106717 | 99.67 | Soil | Switzerland |
| SRR5520921 | SRX2794167 | PRJNA386024 | SRP106717 | 99.33 | Soil | Switzerland |
| SRR5520932 | SRX2794178 | PRJNA386024 | SRP106717 | 99.33 | Soil | Switzerland |
| SRR5520958 | SRX2794204 | PRJNA386024 | SRP106717 | 99.33 | Soil | Switzerland |
| SRR5521229 | SRX2794475 | PRJNA386024 | SRP106717 | 99.33 | Soil | Switzerland |
| SRR8109376 | SRX4935940 | PRJNA498236 | SRP166746 | 100 | Canine gut | Switzerland |
| SRR8109340 | SRX4935978 | PRJNA498236 | SRP166746 | 99.34 | Canine gut | Switzerland |
| SRR12081172 | SRX8608294 | PRJNA641340 | SRP268453 | 100 | Soil | Switzerland |
| ERR1134746 | ERX1213925 | PRJEB11737 | ERP013147 | 99.07 | Soil | United Kingdom |
| ERR1134762 | ERX1213941 | PRJEB11737 | ERP013147 | 99.07 | Soil | United Kingdom |
| ERR1134785 | ERX1213964 | PRJEB11737 | ERP013147 | 99.07 | Soil | United Kingdom |
| ERR1353991 | ERX1425466 | PRJEB13388 | ERP014945 | 99.65 | Soil | United Kingdom |
| ERR2259284 | ERX2311010 | PRJEB24367 | ERP106185 | 99.2 | Rhizosphere | United Kingdom |
| ERR2259290 | ERX2311016 | PRJEB24367 | ERP106185 | 99.6 | Rhizosphere | United Kingdom |
| ERR2259303 | ERX2311029 | PRJEB24367 | ERP106185 | 100 | Rhizosphere | United Kingdom |
| ERR2259305 | ERX2311031 | PRJEB24367 | ERP106185 | 100 | Rhizosphere | United Kingdom |
| ERR2259310 | ERX2311036 | PRJEB24367 | ERP106185 | 99.2 | Rhizosphere | United Kingdom |
| ERR2259315 | ERX2311041 | PRJEB24367 | ERP106185 | 100 | Rhizosphere | United Kingdom |
| ERR2259334 | ERX2311060 | PRJEB24367 | ERP106185 | 100 | Rhizosphere | United Kingdom |
| ERR2259336 | ERX2311062 | PRJEB24367 | ERP106185 | 100 | Rhizosphere | United Kingdom |
| ERR2259339 | ERX2311065 | PRJEB24367 | ERP106185 | 100 | Rhizosphere | United Kingdom |
| ERR2259341 | ERX2311067 | PRJEB24367 | ERP106185 | 100 | Rhizosphere | United Kingdom |
| ERR2259348 | ERX2311074 | PRJEB24367 | ERP106185 | 99.6 | Rhizosphere | United Kingdom |
| ERR2659983 | ERX2676361 | PRJEB27411 | ERP109485 | 100 | Soil | United Kingdom |
| ERR2659984 | ERX2676362 | PRJEB27411 | ERP109485 | 100 | Soil | United Kingdom |
| ERR2659985 | ERX2676363 | PRJEB27411 | ERP109485 | 100 | Soil | United Kingdom |
| ERR2659987 | ERX2676365 | PRJEB27411 | ERP109485 | 100 | Soil | United Kingdom |
| ERR2659988 | ERX2676366 | PRJEB27411 | ERP109485 | 100 | Soil | United Kingdom |
| ERR2659990 | ERX2676368 | PRJEB27411 | ERP109485 | 100 | Soil | United Kingdom |
| ERR2659992 | ERX2676370 | PRJEB27411 | ERP109485 | 99.33 | Soil | United Kingdom |
| ERR2659993 | ERX2676371 | PRJEB27411 | ERP109485 | 100 | Soil | United Kingdom |
| ERR2659994 | ERX2676372 | PRJEB27411 | ERP109485 | 100 | Soil | United Kingdom |
| ERR2659997 | ERX2676375 | PRJEB27411 | ERP109485 | 100 | Soil | United Kingdom |
| ERR2659998 | ERX2676376 | PRJEB27411 | ERP109485 | 100 | Soil | United Kingdom |
| ERR2659999 | ERX2676377 | PRJEB27411 | ERP109485 | 100 | Soil | United Kingdom |
| ERR2660002 | ERX2676380 | PRJEB27411 | ERP109485 | 100 | Soil | United Kingdom |
| ERR2660006 | ERX2676384 | PRJEB27411 | ERP109485 | 100 | Soil | United Kingdom |
| ERR2660007 | ERX2676385 | PRJEB27411 | ERP109485 | 100 | Soil | United Kingdom |
| ERR2660010 | ERX2676388 | PRJEB27411 | ERP109485 | 100 | Soil | United Kingdom |
| ERR2660014 | ERX2676392 | PRJEB27411 | ERP109485 | 100 | Soil | United Kingdom |
| ERR2660017 | ERX2676395 | PRJEB27411 | ERP109485 | 100 | Soil | United Kingdom |
| ERR2660018 | ERX2676396 | PRJEB27411 | ERP109485 | 100 | Soil | United Kingdom |
| ERR2660021 | ERX2676399 | PRJEB27411 | ERP109485 | 100 | Soil | United Kingdom |
| ERR2660022 | ERX2676400 | PRJEB27411 | ERP109485 | 100 | Soil | United Kingdom |
| ERR2660023 | ERX2676401 | PRJEB27411 | ERP109485 | 100 | Soil | United Kingdom |
| ERR2660025 | ERX2676403 | PRJEB27411 | ERP109485 | 100 | Soil | United Kingdom |
| ERR2660027 | ERX2676405 | PRJEB27411 | ERP109485 | 100 | Soil | United Kingdom |
| ERR2660028 | ERX2676406 | PRJEB27411 | ERP109485 | 100 | Soil | United Kingdom |
| ERR2660029 | ERX2676407 | PRJEB27411 | ERP109485 | 100 | Soil | United Kingdom |
| ERR2660030 | ERX2676408 | PRJEB27411 | ERP109485 | 100 | Soil | United Kingdom |
| ERR2660031 | ERX2676409 | PRJEB27411 | ERP109485 | 99.67 | Soil | United Kingdom |
| ERR2660032 | ERX2676410 | PRJEB27411 | ERP109485 | 100 | Soil | United Kingdom |
| ERR2660033 | ERX2676411 | PRJEB27411 | ERP109485 | 100 | Soil | United Kingdom |
| ERR2660034 | ERX2676412 | PRJEB27411 | ERP109485 | 100 | Soil | United Kingdom |
| ERR2660037 | ERX2676415 | PRJEB27411 | ERP109485 | 100 | Soil | United Kingdom |
| ERR2660038 | ERX2676416 | PRJEB27411 | ERP109485 | 100 | Soil | United Kingdom |
| ERR2660039 | ERX2676417 | PRJEB27411 | ERP109485 | 100 | Soil | United Kingdom |
| ERR2660042 | ERX2676420 | PRJEB27411 | ERP109485 | 100 | Soil | United Kingdom |
| ERR2660044 | ERX2676422 | PRJEB27411 | ERP109485 | 100 | Soil | United Kingdom |
| ERR2660045 | ERX2676423 | PRJEB27411 | ERP109485 | 100 | Soil | United Kingdom |
| ERR2660046 | ERX2676424 | PRJEB27411 | ERP109485 | 100 | Soil | United Kingdom |
| ERR2660049 | ERX2676427 | PRJEB27411 | ERP109485 | 100 | Soil | United Kingdom |
| ERR2660050 | ERX2676428 | PRJEB27411 | ERP109485 | 100 | Soil | United Kingdom |
| ERR2660052 | ERX2676430 | PRJEB27411 | ERP109485 | 100 | Soil | United Kingdom |
| ERR2660053 | ERX2676431 | PRJEB27411 | ERP109485 | 100 | Soil | United Kingdom |
| ERR2660054 | ERX2676432 | PRJEB27411 | ERP109485 | 99.67 | Soil | United Kingdom |
| ERR2660057 | ERX2676435 | PRJEB27411 | ERP109485 | 100 | Soil | United Kingdom |
| ERR2660059 | ERX2676437 | PRJEB27411 | ERP109485 | 100 | Soil | United Kingdom |
| ERR2660061 | ERX2676439 | PRJEB27411 | ERP109485 | 100 | Soil | United Kingdom |
| ERR2660063 | ERX2676441 | PRJEB27411 | ERP109485 | 100 | Soil | United Kingdom |
| ERR2660064 | ERX2676442 | PRJEB27411 | ERP109485 | 100 | Soil | United Kingdom |
| ERR2660065 | ERX2676443 | PRJEB27411 | ERP109485 | 100 | Soil | United Kingdom |
| ERR2660070 | ERX2676448 | PRJEB27411 | ERP109485 | 99.67 | Soil | United Kingdom |
| ERR2660077 | ERX2676455 | PRJEB27411 | ERP109485 | 100 | Soil | United Kingdom |
| ERR2660078 | ERX2676456 | PRJEB27411 | ERP109485 | 100 | Soil | United Kingdom |
| ERR2660079 | ERX2676457 | PRJEB27411 | ERP109485 | 100 | Soil | United Kingdom |
| ERR2660085 | ERX2676463 | PRJEB27411 | ERP109485 | 100 | Soil | United Kingdom |
| ERR2660087 | ERX2676465 | PRJEB27411 | ERP109485 | 100 | Soil | United Kingdom |
| ERR2660088 | ERX2676466 | PRJEB27411 | ERP109485 | 99.67 | Soil | United Kingdom |
| ERR2660089 | ERX2676467 | PRJEB27411 | ERP109485 | 100 | Soil | United Kingdom |
| ERR2660091 | ERX2676469 | PRJEB27411 | ERP109485 | 100 | Soil | United Kingdom |
| ERR2660092 | ERX2676470 | PRJEB27411 | ERP109485 | 100 | Soil | United Kingdom |
| ERR2660094 | ERX2676472 | PRJEB27411 | ERP109485 | 100 | Soil | United Kingdom |
| ERR2660095 | ERX2676473 | PRJEB27411 | ERP109485 | 100 | Soil | United Kingdom |
| ERR2660096 | ERX2676474 | PRJEB27411 | ERP109485 | 100 | Soil | United Kingdom |
| ERR2660097 | ERX2676475 | PRJEB27411 | ERP109485 | 100 | Soil | United Kingdom |
| ERR2660098 | ERX2676476 | PRJEB27411 | ERP109485 | 100 | Soil | United Kingdom |
| ERR2660100 | ERX2676478 | PRJEB27411 | ERP109485 | 100 | Soil | United Kingdom |
| ERR2660101 | ERX2676479 | PRJEB27411 | ERP109485 | 100 | Soil | United Kingdom |
| ERR2660103 | ERX2676481 | PRJEB27411 | ERP109485 | 100 | Soil | United Kingdom |
| ERR2660104 | ERX2676482 | PRJEB27411 | ERP109485 | 100 | Soil | United Kingdom |
| ERR2660108 | ERX2676486 | PRJEB27411 | ERP109485 | 100 | Soil | United Kingdom |
| ERR2660111 | ERX2676489 | PRJEB27411 | ERP109485 | 100 | Soil | United Kingdom |
| ERR2660113 | ERX2676491 | PRJEB27411 | ERP109485 | 100 | Soil | United Kingdom |
| ERR2660114 | ERX2676492 | PRJEB27411 | ERP109485 | 100 | Soil | United Kingdom |
| ERR2660115 | ERX2676493 | PRJEB27411 | ERP109485 | 100 | Soil | United Kingdom |
| ERR2660116 | ERX2676494 | PRJEB27411 | ERP109485 | 100 | Soil | United Kingdom |
| ERR2660117 | ERX2676495 | PRJEB27411 | ERP109485 | 100 | Soil | United Kingdom |
| ERR2660118 | ERX2676496 | PRJEB27411 | ERP109485 | 100 | Soil | United Kingdom |
| ERR2660119 | ERX2676497 | PRJEB27411 | ERP109485 | 100 | Soil | United Kingdom |
| ERR2660121 | ERX2676499 | PRJEB27411 | ERP109485 | 100 | Soil | United Kingdom |
| ERR2660122 | ERX2676500 | PRJEB27411 | ERP109485 | 100 | Soil | United Kingdom |
| ERR2660124 | ERX2676502 | PRJEB27411 | ERP109485 | 100 | Soil | United Kingdom |
| ERR2660125 | ERX2676503 | PRJEB27411 | ERP109485 | 100 | Soil | United Kingdom |
| ERR2660127 | ERX2676505 | PRJEB27411 | ERP109485 | 100 | Soil | United Kingdom |
| ERR2660128 | ERX2676506 | PRJEB27411 | ERP109485 | 100 | Soil | United Kingdom |
| ERR2660129 | ERX2676507 | PRJEB27411 | ERP109485 | 100 | Soil | United Kingdom |
| ERR2660132 | ERX2676510 | PRJEB27411 | ERP109485 | 100 | Soil | United Kingdom |
| ERR2660133 | ERX2676511 | PRJEB27411 | ERP109485 | 100 | Soil | United Kingdom |
| ERR2660136 | ERX2676514 | PRJEB27411 | ERP109485 | 100 | Soil | United Kingdom |
| ERR2660138 | ERX2676516 | PRJEB27411 | ERP109485 | 100 | Soil | United Kingdom |
| ERR2660139 | ERX2676517 | PRJEB27411 | ERP109485 | 100 | Soil | United Kingdom |
| ERR2660142 | ERX2676520 | PRJEB27411 | ERP109485 | 100 | Soil | United Kingdom |
| ERR2660144 | ERX2676522 | PRJEB27411 | ERP109485 | 100 | Soil | United Kingdom |
| ERR2660146 | ERX2676524 | PRJEB27411 | ERP109485 | 100 | Soil | United Kingdom |
| ERR2660147 | ERX2676525 | PRJEB27411 | ERP109485 | 100 | Soil | United Kingdom |
| ERR2660149 | ERX2676527 | PRJEB27411 | ERP109485 | 100 | Soil | United Kingdom |
| ERR2660152 | ERX2676530 | PRJEB27411 | ERP109485 | 100 | Soil | United Kingdom |
| ERR2660155 | ERX2676533 | PRJEB27411 | ERP109485 | 99.66 | Soil | United Kingdom |
| ERR2660158 | ERX2676536 | PRJEB27411 | ERP109485 | 100 | Soil | United Kingdom |
| ERR2660161 | ERX2676539 | PRJEB27411 | ERP109485 | 100 | Soil | United Kingdom |
| ERR2660165 | ERX2676543 | PRJEB27411 | ERP109485 | 100 | Soil | United Kingdom |
| ERR2660169 | ERX2676547 | PRJEB27411 | ERP109485 | 100 | Soil | United Kingdom |
| ERR2660171 | ERX2676549 | PRJEB27411 | ERP109485 | 100 | Soil | United Kingdom |
| ERR2660172 | ERX2676550 | PRJEB27411 | ERP109485 | 100 | Soil | United Kingdom |
| ERR2660173 | ERX2676551 | PRJEB27411 | ERP109485 | 100 | Soil | United Kingdom |
| ERR2660174 | ERX2676552 | PRJEB27411 | ERP109485 | 100 | Soil | United Kingdom |
| ERR2660177 | ERX2676555 | PRJEB27411 | ERP109485 | 100 | Soil | United Kingdom |
| ERR2660181 | ERX2676559 | PRJEB27411 | ERP109485 | 100 | Soil | United Kingdom |
| ERR2660183 | ERX2676561 | PRJEB27411 | ERP109485 | 100 | Soil | United Kingdom |
| ERR2660184 | ERX2676562 | PRJEB27411 | ERP109485 | 100 | Soil | United Kingdom |
| ERR2660186 | ERX2676564 | PRJEB27411 | ERP109485 | 100 | Soil | United Kingdom |
| ERR2660187 | ERX2676565 | PRJEB27411 | ERP109485 | 100 | Soil | United Kingdom |
| ERR2660188 | ERX2676566 | PRJEB27411 | ERP109485 | 100 | Soil | United Kingdom |
| ERR2660189 | ERX2676567 | PRJEB27411 | ERP109485 | 100 | Soil | United Kingdom |
| ERR2660191 | ERX2676569 | PRJEB27411 | ERP109485 | 100 | Soil | United Kingdom |
| ERR2660194 | ERX2676572 | PRJEB27411 | ERP109485 | 100 | Soil | United Kingdom |
| ERR2660196 | ERX2676574 | PRJEB27411 | ERP109485 | 100 | Soil | United Kingdom |
| ERR2660198 | ERX2676576 | PRJEB27411 | ERP109485 | 100 | Soil | United Kingdom |
| ERR2660208 | ERX2676586 | PRJEB27411 | ERP109485 | 100 | Soil | United Kingdom |
| ERR2660211 | ERX2676589 | PRJEB27411 | ERP109485 | 100 | Soil | United Kingdom |
| ERR2660212 | ERX2676590 | PRJEB27411 | ERP109485 | 100 | Soil | United Kingdom |
| ERR2660213 | ERX2676591 | PRJEB27411 | ERP109485 | 100 | Soil | United Kingdom |
| ERR2660215 | ERX2676593 | PRJEB27411 | ERP109485 | 100 | Soil | United Kingdom |
| ERR2660218 | ERX2676596 | PRJEB27411 | ERP109485 | 100 | Soil | United Kingdom |
| ERR2660219 | ERX2676597 | PRJEB27411 | ERP109485 | 100 | Soil | United Kingdom |
| ERR2660229 | ERX2676607 | PRJEB27411 | ERP109485 | 100 | Soil | United Kingdom |
| ERR2660233 | ERX2676611 | PRJEB27411 | ERP109485 | 100 | Soil | United Kingdom |
| ERR2660235 | ERX2676613 | PRJEB27411 | ERP109485 | 100 | Soil | United Kingdom |
| ERR2660238 | ERX2676616 | PRJEB27411 | ERP109485 | 100 | Soil | United Kingdom |
| ERR2660240 | ERX2676618 | PRJEB27411 | ERP109485 | 100 | Soil | United Kingdom |
| ERR2660243 | ERX2676621 | PRJEB27411 | ERP109485 | 100 | Soil | United Kingdom |
| ERR2660247 | ERX2676625 | PRJEB27411 | ERP109485 | 100 | Soil | United Kingdom |
| ERR2660254 | ERX2676632 | PRJEB27411 | ERP109485 | 100 | Soil | United Kingdom |
| ERR2660255 | ERX2676633 | PRJEB27411 | ERP109485 | 100 | Soil | United Kingdom |
| ERR2660260 | ERX2676638 | PRJEB27411 | ERP109485 | 99.67 | Soil | United Kingdom |
| ERR2660261 | ERX2676639 | PRJEB27411 | ERP109485 | 100 | Soil | United Kingdom |
| ERR2757425 | ERX2770418 | PRJEB28028 | ERP110188 | 100 | Soil | United Kingdom |
| ERR2757432 | ERX2770425 | PRJEB28028 | ERP110188 | 99.59 | Soil | United Kingdom |
| ERR2757484 | ERX2770477 | PRJEB28028 | ERP110188 | 100 | Soil | United Kingdom |
| ERR2757486 | ERX2770479 | PRJEB28028 | ERP110188 | 99.18 | Soil | United Kingdom |
| ERR2757491 | ERX2770484 | PRJEB28028 | ERP110188 | 100 | Soil | United Kingdom |
| ERR2757532 | ERX2770525 | PRJEB28028 | ERP110188 | 100 | Soil | United Kingdom |
| ERR2757539 | ERX2770532 | PRJEB28028 | ERP110188 | 100 | Soil | United Kingdom |
| ERR2757601 | ERX2770594 | PRJEB28028 | ERP110188 | 99.6 | Soil | United Kingdom |
| ERR2757619 | ERX2770612 | PRJEB28028 | ERP110188 | 99.6 | Soil | United Kingdom |
| ERR2757622 | ERX2770615 | PRJEB28028 | ERP110188 | 99.6 | Soil | United Kingdom |
| ERR2757625 | ERX2770618 | PRJEB28028 | ERP110188 | 99.6 | Soil | United Kingdom |
| ERR2757630 | ERX2770623 | PRJEB28028 | ERP110188 | 99.6 | Soil | United Kingdom |
| ERR2757633 | ERX2770626 | PRJEB28028 | ERP110188 | 99.6 | Soil | United Kingdom |
| ERR2757639 | ERX2770632 | PRJEB28028 | ERP110188 | 99.6 | Soil | United Kingdom |
| ERR2757655 | ERX2770648 | PRJEB28028 | ERP110188 | 99.6 | Soil | United Kingdom |
| ERR2757701 | ERX2770694 | PRJEB28028 | ERP110188 | 99.6 | Soil | United Kingdom |
| ERR2757702 | ERX2770695 | PRJEB28028 | ERP110188 | 99.6 | Soil | United Kingdom |
| ERR2757724 | ERX2770717 | PRJEB28028 | ERP110188 | 99.6 | Soil | United Kingdom |
| ERR2757741 | ERX2770734 | PRJEB28028 | ERP110188 | 99.6 | Soil | United Kingdom |
| ERR2757761 | ERX2770754 | PRJEB28028 | ERP110188 | 99.6 | Soil | United Kingdom |
| ERR2757773 | ERX2770766 | PRJEB28028 | ERP110188 | 99.6 | Soil | United Kingdom |
| ERR2757780 | ERX2770773 | PRJEB28028 | ERP110188 | 99.6 | Soil | United Kingdom |
| ERR2757795 | ERX2770788 | PRJEB28028 | ERP110188 | 99.6 | Soil | United Kingdom |
| ERR2757813 | ERX2770806 | PRJEB28028 | ERP110188 | 100 | Soil | United Kingdom |
| ERR2757814 | ERX2770807 | PRJEB28028 | ERP110188 | 100 | Soil | United Kingdom |
| ERR2757819 | ERX2770812 | PRJEB28028 | ERP110188 | 99.6 | Soil | United Kingdom |
| ERR2757822 | ERX2770815 | PRJEB28028 | ERP110188 | 99.6 | Soil | United Kingdom |
| ERR2757836 | ERX2770829 | PRJEB28028 | ERP110188 | 99.6 | Soil | United Kingdom |
| ERR2757841 | ERX2770834 | PRJEB28028 | ERP110188 | 99.6 | Soil | United Kingdom |
| ERR3063087 | ERX3135888 | PRJEB29902 | ERP112260 | 100 | Rhizosphere | United Kingdom |
| ERR3063088 | ERX3135889 | PRJEB29902 | ERP112260 | 100 | Rhizosphere | United Kingdom |
| SRR8869564 | SRX5656333 | PRJNA531648 | SRP191530 | 100 | Soil | United Kingdom |
| SRR8869416 | SRX5656481 | PRJNA531648 | SRP191530 | 100 | Soil | United Kingdom |
| SRR8869342 | SRX5656555 | PRJNA531648 | SRP191530 | 100 | Soil | United Kingdom |
| SRR8869339 | SRX5656558 | PRJNA531648 | SRP191530 | 100 | Soil | United Kingdom |
| SRR9276993 | SRX6046521 | PRJNA548438 | SRP201165 | 99.66 | Rhizosphere | United Kingdom |
| SRR9276978 | SRX6046536 | PRJNA548438 | SRP201165 | 99.33 | Rhizosphere | United Kingdom |
| SRR9276961 | SRX6046552 | PRJNA548438 | SRP201165 | 100 | Rhizosphere | United Kingdom |
| SRR9276959 | SRX6046554 | PRJNA548438 | SRP201165 | 99.67 | Rhizosphere | United Kingdom |
| SRR9276910 | SRX6046603 | PRJNA548438 | SRP201165 | 99.33 | Soil | United Kingdom |
| SRR9276908 | SRX6046605 | PRJNA548438 | SRP201165 | 99.33 | Soil | United Kingdom |
| SRR9276904 | SRX6046609 | PRJNA548438 | SRP201165 | 99.67 | Soil | United Kingdom |
| SRR9276901 | SRX6046612 | PRJNA548438 | SRP201165 | 100 | Soil | United Kingdom |
| SRR9276887 | SRX6046626 | PRJNA548438 | SRP201165 | 99.66 | Rhizosphere | United Kingdom |
| SRR9276885 | SRX6046628 | PRJNA548438 | SRP201165 | 99.33 | Rhizosphere | United Kingdom |
| SRR9276862 | SRX6046651 | PRJNA548438 | SRP201165 | 99.33 | Soil | United Kingdom |
| SRR9276851 | SRX6046662 | PRJNA548438 | SRP201165 | 99.67 | Soil | United Kingdom |
| SRR9276819 | SRX6046694 | PRJNA548438 | SRP201165 | 100 | Soil | United Kingdom |
| SRR9276792 | SRX6046721 | PRJNA548438 | SRP201165 | 100 | Rhizosphere | United Kingdom |
| SRR9276791 | SRX6046722 | PRJNA548438 | SRP201165 | 99.67 | Rhizosphere | United Kingdom |
| SRR9276790 | SRX6046723 | PRJNA548438 | SRP201165 | 100 | Rhizosphere | United Kingdom |
| SRR9276783 | SRX6046730 | PRJNA548438 | SRP201165 | 99.67 | Rhizosphere | United Kingdom |
| SRR9276780 | SRX6046733 | PRJNA548438 | SRP201165 | 99.3 | Rhizosphere | United Kingdom |
| SRR9276766 | SRX6046747 | PRJNA548438 | SRP201165 | 100 | Soil | United Kingdom |
| SRR9276762 | SRX6046751 | PRJNA548438 | SRP201165 | 100 | Soil | United Kingdom |
| SRR9276761 | SRX6046752 | PRJNA548438 | SRP201165 | 100 | Soil | United Kingdom |
| SRR9276758 | SRX6046755 | PRJNA548438 | SRP201165 | 99.33 | Soil | United Kingdom |
| SRR9276749 | SRX6046764 | PRJNA548438 | SRP201165 | 100 | Soil | United Kingdom |
| SRR9277246 | SRX6046778 | PRJNA548438 | SRP201165 | 100 | Soil | United Kingdom |
| SRR9277243 | SRX6046781 | PRJNA548438 | SRP201165 | 100 | Soil | United Kingdom |
| SRR9277220 | SRX6046804 | PRJNA548438 | SRP201165 | 100 | Soil | United Kingdom |
| SRR9277208 | SRX6046816 | PRJNA548438 | SRP201165 | 100 | Soil | United Kingdom |
| SRR9277205 | SRX6046819 | PRJNA548438 | SRP201165 | 99.67 | Rhizosphere | United Kingdom |
| SRR9277182 | SRX6046842 | PRJNA548438 | SRP201165 | 99.33 | Soil | United Kingdom |
| SRR9277063 | SRX6046961 | PRJNA548438 | SRP201165 | 100 | Rhizosphere | United Kingdom |
| SRR9277061 | SRX6046963 | PRJNA548438 | SRP201165 | 99.33 | Rhizosphere | United Kingdom |
| SRR9277060 | SRX6046964 | PRJNA548438 | SRP201165 | 99.67 | Rhizosphere | United Kingdom |
| SRR9277031 | SRX6046993 | PRJNA548438 | SRP201165 | 100 | Soil | United Kingdom |
| SRR9277269 | SRX6047052 | PRJNA548438 | SRP201165 | 99.67 | Soil | United Kingdom |
| SRR9277259 | SRX6047062 | PRJNA548438 | SRP201165 | 99.33 | Rhizosphere | United Kingdom |
| SRR10902220 | SRX7570377 | PRJNA594906 | SRP241329 | 100 | Soil | United Kingdom |
| SRR10902219 | SRX7570378 | PRJNA594906 | SRP241329 | 99.65 | Soil | United Kingdom |
| SRR10902215 | SRX7570382 | PRJNA594906 | SRP241329 | 99.66 | Soil | United Kingdom |
| SRR10902214 | SRX7570383 | PRJNA594906 | SRP241329 | 99.66 | Soil | United Kingdom |
| SRR10902213 | SRX7570384 | PRJNA594906 | SRP241329 | 99.31 | Soil | United Kingdom |
| SRR10902212 | SRX7570385 | PRJNA594906 | SRP241329 | 99.65 | Soil | United Kingdom |
| SRR10902211 | SRX7570386 | PRJNA594906 | SRP241329 | 99.66 | Soil | United Kingdom |
| SRR10902210 | SRX7570387 | PRJNA594906 | SRP241329 | 100 | Soil | United Kingdom |
| SRR10902204 | SRX7570393 | PRJNA594906 | SRP241329 | 99.66 | Soil | United Kingdom |
| SRR10902203 | SRX7570394 | PRJNA594906 | SRP241329 | 99.65 | Soil | United Kingdom |
| SRR10902646 | SRX7570803 | PRJNA594906 | SRP241329 | 100 | Soil | United Kingdom |
| SRR10902645 | SRX7570804 | PRJNA594906 | SRP241329 | 100 | Soil | United Kingdom |
| SRR10902642 | SRX7570807 | PRJNA594906 | SRP241329 | 100 | Soil | United Kingdom |
| SRR10902641 | SRX7570808 | PRJNA594906 | SRP241329 | 100 | Soil | United Kingdom |
| SRR10902640 | SRX7570809 | PRJNA594906 | SRP241329 | 100 | Soil | United Kingdom |
| SRR10902638 | SRX7570811 | PRJNA594906 | SRP241329 | 100 | Soil | United Kingdom |
| SRR10902637 | SRX7570812 | PRJNA594906 | SRP241329 | 100 | Soil | United Kingdom |
| SRR10902636 | SRX7570813 | PRJNA594906 | SRP241329 | 100 | Soil | United Kingdom |
| SRR10902635 | SRX7570814 | PRJNA594906 | SRP241329 | 99.65 | Soil | United Kingdom |
| SRR10902633 | SRX7570816 | PRJNA594906 | SRP241329 | 100 | Soil | United Kingdom |
| SRR10902632 | SRX7570817 | PRJNA594906 | SRP241329 | 99.31 | Soil | United Kingdom |
| SRR10902630 | SRX7570819 | PRJNA594906 | SRP241329 | 99.31 | Soil | United Kingdom |
| SRR4054326 | SRX2043901 | PRJNA339869 | SRP082695 | 99.2 | Soil | United States of America |
| SRR4054358 | SRX2043933 | PRJNA339869 | SRP082695 | 99.2 | Soil | United States of America |
| SRR5434090 | SRX2724125 | PRJNA381709 | SRP103512 | 99.15 | Soil | United States of America |
| SRR6039003 | SRX3186916 | PRJNA384978 | SRP117683 | 100 | Soil | United States of America |
| SRR6038888 | SRX3187031 | PRJNA384978 | SRP117683 | 99.74 | Soil | United States of America |
| SRR6038884 | SRX3187035 | PRJNA384978 | SRP117683 | 99.74 | Soil | United States of America |
| SRR6038877 | SRX3187042 | PRJNA384978 | SRP117683 | 99.74 | Soil | United States of America |
| SRR6038873 | SRX3187046 | PRJNA384978 | SRP117683 | 99.48 | Soil | United States of America |
| SRR6038872 | SRX3187047 | PRJNA384978 | SRP117683 | 100 | Soil | United States of America |
| SRR6038871 | SRX3187048 | PRJNA384978 | SRP117683 | 100 | Soil | United States of America |
| SRR6038784 | SRX3187135 | PRJNA384978 | SRP117683 | 100 | Soil | United States of America |
| SRR6038779 | SRX3187140 | PRJNA384978 | SRP117683 | 100 | Soil | United States of America |
| SRR6038778 | SRX3187141 | PRJNA384978 | SRP117683 | 100 | Soil | United States of America |
| SRR6038761 | SRX3187158 | PRJNA384978 | SRP117683 | 99.74 | Soil | United States of America |
| SRR6152084 | SRX3263810 | PRJNA413435 | SRP119643 | 99.6 | Soil | United States of America |
| SRR6152050 | SRX3263844 | PRJNA413435 | SRP119643 | 99.6 | Soil | United States of America |
| SRR6152045 | SRX3263849 | PRJNA413435 | SRP119643 | 99.6 | Soil | United States of America |
| SRR6152043 | SRX3263851 | PRJNA413435 | SRP119643 | 99.6 | Soil | United States of America |
| SRR6152041 | SRX3263853 | PRJNA413435 | SRP119643 | 100 | Soil | United States of America |
| SRR6151932 | SRX3263962 | PRJNA413435 | SRP119643 | 99.21 | Soil | United States of America |
| SRR6151930 | SRX3263964 | PRJNA413435 | SRP119643 | 99.6 | Soil | United States of America |
| SRR6151926 | SRX3263968 | PRJNA413435 | SRP119643 | 99.6 | Soil | United States of America |
| SRR6151893 | SRX3264001 | PRJNA413435 | SRP119643 | 99.6 | Soil | United States of America |
| SRR6151889 | SRX3264005 | PRJNA413435 | SRP119643 | 99.6 | Soil | United States of America |
| SRR6151888 | SRX3264006 | PRJNA413435 | SRP119643 | 99.6 | Soil | United States of America |
| SRR6151862 | SRX3264032 | PRJNA413435 | SRP119643 | 99.2 | Soil | United States of America |
| SRR6151859 | SRX3264035 | PRJNA413435 | SRP119643 | 99.6 | Soil | United States of America |
| SRR6151850 | SRX3264044 | PRJNA413435 | SRP119643 | 99.6 | Soil | United States of America |
| SRR6489079 | SRX3578909 | PRJNA430124 | SRP129682 | 99.6 | Soil | United States of America |
| SRR6807241 | SRX3765559 | PRJNA436977 | SRP133948 | 99.32 | Soil | United States of America |
| SRR7278354 | SRX4181725 | PRJNA475148 | SRP150020 | 99.6 | Soil | United States of America |
| SRR7278283 | SRX4181796 | PRJNA475148 | SRP150020 | 99.2 | Soil | United States of America |
| SRR7278402 | SRX4182058 | PRJNA475148 | SRP150020 | 99.2 | Soil | United States of America |
| SRR7401924 | SRX4274041 | PRJNA477003 | SRP150950 | 100 | Sewage sludge | United States of America |
| SRR7401923 | SRX4274042 | PRJNA477003 | SRP150950 | 100 | Sewage sludge | United States of America |
| SRR7401917 | SRX4274049 | PRJNA477003 | SRP150950 | 100 | Sewage sludge | United States of America |
| SRR7401913 | SRX4274053 | PRJNA477003 | SRP150950 | 100 | Sewage sludge | United States of America |
| SRR7401912 | SRX4274054 | PRJNA477003 | SRP150950 | 100 | Sewage sludge | United States of America |
| SRR7401910 | SRX4274056 | PRJNA477003 | SRP150950 | 100 | Sewage sludge | United States of America |
| SRR7401909 | SRX4274057 | PRJNA477003 | SRP150950 | 99.2 | Sewage sludge | United States of America |
| SRR7401908 | SRX4274058 | PRJNA477003 | SRP150950 | 100 | Sewage sludge | United States of America |
| SRR7401896 | SRX4274070 | PRJNA477003 | SRP150950 | 99.6 | Sewage sludge | United States of America |
| SRR7401888 | SRX4274078 | PRJNA477003 | SRP150950 | 99.6 | Soil | United States of America |
| SRR7401871 | SRX4274095 | PRJNA477003 | SRP150950 | 100 | Sewage sludge | United States of America |
| SRR7401870 | SRX4274096 | PRJNA477003 | SRP150950 | 99.2 | Sewage sludge | United States of America |
| SRR7401864 | SRX4274102 | PRJNA477003 | SRP150950 | 100 | Sewage sludge | United States of America |
| SRR7771718 | SRX4627076 | PRJNA481943 | SRP159198 | 99.32 | Soil | United States of America |
| SRR7771700 | SRX4627094 | PRJNA481943 | SRP159198 | 99.32 | Soil | United States of America |
| SRR7771664 | SRX4627130 | PRJNA481943 | SRP159198 | 99.32 | Soil | United States of America |
| SRR7771658 | SRX4627136 | PRJNA481943 | SRP159198 | 99.32 | Soil | United States of America |
| SRR8056768 | SRX4886342 | PRJNA496343 | SRP165754 | 99.33 | Soil | United States of America |
| SRR8056762 | SRX4886348 | PRJNA496343 | SRP165754 | 99 | Soil | United States of America |
| SRR8056761 | SRX4886349 | PRJNA496343 | SRP165754 | 99.33 | Soil | United States of America |
| SRR8056756 | SRX4886354 | PRJNA496343 | SRP165754 | 99.32 | Soil | United States of America |
| SRR8697215 | SRX5493074 | PRJNA525991 | SRP187873 | 99.32 | Soil | United States of America |
| SRR8697214 | SRX5493075 | PRJNA525991 | SRP187873 | 99.32 | Soil | United States of America |
| SRR8793922 | SRX5583427 | PRJNA522791 | SRP189183 | 99.7 | Soil | United States of America |
| SRR9695324 | SRX6453581 | PRJNA551045 | SRP214977 | 99.6 | Soil | United States of America |
| SRR10532239 | SRX7216270 | PRJNA590039 | SRP230287 | 99.63 | Rhizosphere | United States of America |
| SRR10870231 | SRX7540016 | PRJNA599388 | SRP241200 | 99.66 | Rhizosphere | United States of America |
| SRR10870193 | SRX7540054 | PRJNA599388 | SRP241200 | 99.32 | Rhizosphere | United States of America |
| SRR10870191 | SRX7540056 | PRJNA599388 | SRP241200 | 99.66 | Rhizosphere | United States of America |
| SRR10870182 | SRX7540065 | PRJNA599388 | SRP241200 | 99.66 | Rhizosphere | United States of America |
| SRR10870023 | SRX7540224 | PRJNA599388 | SRP241200 | 99.66 | Rhizosphere | United States of America |
| SRR10909327 | SRX7577069 | PRJNA601975 | SRP242588 | 99.2 | Soil | United States of America |
| SRR10963966 | SRX7629881 | PRJNA603147 | SRP244753 | 99.38 | Rhizosphere | United States of America |
| SRR10965287 | SRX7631101 | PRJNA603199 | SRP245026 | 100 | Rhizosphere | United States of America |
| SRR11101111 | SRX7739586 | PRJNA606949 | SRP249824 | 99.65 | Soil | United States of America |
| SRR11101106 | SRX7739591 | PRJNA606949 | SRP249824 | 99.64 | Soil | United States of America |
| SRR11406304 | SRX7984846 | PRJNA512838 | SRP175094 | 99.28 | Chicken gut | United States of America |
| SRR11406239 | SRX7984911 | PRJNA512838 | SRP175094 | 100 | Chicken gut | United States of America |
| SRR11406232 | SRX7984918 | PRJNA512838 | SRP175094 | 100 | Chicken gut | United States of America |
| SRR11406182 | SRX7984968 | PRJNA512838 | SRP175094 | 100 | Chicken gut | United States of America |
| SRR11406440 | SRX7985128 | PRJNA512838 | SRP175094 | 100 | Chicken gut | United States of America |
| SRR11616687 | SRX8182836 | PRJNA628127 | SRP258749 | 99.32 | Soil | United States of America |
| SRR11616678 | SRX8182845 | PRJNA628127 | SRP258749 | 99 | Soil | United States of America |
| SRR11616665 | SRX8182858 | PRJNA628127 | SRP258749 | 99.31 | Soil | United States of America |
| SRR11880089 | SRX8429376 | PRJNA635685 | SRP265318 | 100 | Soil | United States of America |
| SRR11880086 | SRX8429379 | PRJNA635685 | SRP265318 | 100 | Soil | United States of America |
| SRR11880084 | SRX8429381 | PRJNA635685 | SRP265318 | 100 | Soil | United States of America |
| SRR11880083 | SRX8429382 | PRJNA635685 | SRP265318 | 100 | Soil | United States of America |
| SRR11880081 | SRX8429384 | PRJNA635685 | SRP265318 | 100 | Soil | United States of America |
| SRR11880077 | SRX8429388 | PRJNA635685 | SRP265318 | 100 | Soil | United States of America |
| SRR11880075 | SRX8429390 | PRJNA635685 | SRP265318 | 100 | Soil | United States of America |
| SRR11880072 | SRX8429393 | PRJNA635685 | SRP265318 | 100 | Soil | United States of America |
| SRR11880069 | SRX8429396 | PRJNA635685 | SRP265318 | 100 | Soil | United States of America |
| SRR11880066 | SRX8429399 | PRJNA635685 | SRP265318 | 100 | Soil | United States of America |
| SRR11880065 | SRX8429400 | PRJNA635685 | SRP265318 | 100 | Soil | United States of America |
| SRR11880063 | SRX8429402 | PRJNA635685 | SRP265318 | 100 | Soil | United States of America |
| SRR11880058 | SRX8429407 | PRJNA635685 | SRP265318 | 100 | Soil | United States of America |
| SRR11880053 | SRX8429412 | PRJNA635685 | SRP265318 | 100 | Soil | United States of America |
| SRR11880052 | SRX8429413 | PRJNA635685 | SRP265318 | 100 | Soil | United States of America |
| SRR11880051 | SRX8429414 | PRJNA635685 | SRP265318 | 99.58 | Soil | United States of America |
| SRR11880050 | SRX8429415 | PRJNA635685 | SRP265318 | 99.6 | Soil | United States of America |
| SRR11880047 | SRX8429418 | PRJNA635685 | SRP265318 | 100 | Soil | United States of America |
| SRR11880045 | SRX8429420 | PRJNA635685 | SRP265318 | 100 | Soil | United States of America |
| SRR11880040 | SRX8429425 | PRJNA635685 | SRP265318 | 100 | Soil | United States of America |
| SRR11880038 | SRX8429427 | PRJNA635685 | SRP265318 | 100 | Soil | United States of America |
| SRR11880034 | SRX8429431 | PRJNA635685 | SRP265318 | 100 | Soil | United States of America |
| SRR11880028 | SRX8429437 | PRJNA635685 | SRP265318 | 100 | Soil | United States of America |
| SRR11880027 | SRX8429438 | PRJNA635685 | SRP265318 | 99.6 | Soil | United States of America |
| SRR11880022 | SRX8429443 | PRJNA635685 | SRP265318 | 100 | Soil | United States of America |
| SRR11880014 | SRX8429451 | PRJNA635685 | SRP265318 | 100 | Soil | United States of America |
| SRR11880008 | SRX8429457 | PRJNA635685 | SRP265318 | 100 | Soil | United States of America |
| SRR11879998 | SRX8429467 | PRJNA635685 | SRP265318 | 100 | Soil | United States of America |
| SRR11879997 | SRX8429468 | PRJNA635685 | SRP265318 | 99.58 | Soil | United States of America |
| SRR11879995 | SRX8429470 | PRJNA635685 | SRP265318 | 100 | Soil | United States of America |
| SRR11879994 | SRX8429471 | PRJNA635685 | SRP265318 | 100 | Soil | United States of America |
| SRR11879993 | SRX8429472 | PRJNA635685 | SRP265318 | 100 | Soil | United States of America |
